# Methods for Heart Rate Variability Biofeedback (HRVB): A Systematic Review and Guidelines

**DOI:** 10.1007/s10484-023-09582-6

**Published:** 2023-03-14

**Authors:** Jaume F. Lalanza, Sonia Lorente, Raimon Bullich, Carlos García, Josep-Maria Losilla, Lluis Capdevila

**Affiliations:** 1 Department of Basic Psychology, Universitat Autònoma de Barcelona, Bellaterra, Spain; 2 Department of Psychology, UiT The Arctic University of Norway, Tromsø, Norway; 3 Department of Psychobiology and Methodology of Health Science, Universitat Autònoma de Barcelona, Bellaterra, Spain; 4 Pediatric Area, Hospital de Terrassa, Consorci Sanitari de Terrassa, Terrassa, Spain; 5 Sport Research Institute UAB, Universitat Autònoma de Barcelona, Bellaterra, Spain; 6 Departament of Basic Psychology, Universitat Autònoma de Barcelona, 08193 Bellaterra, Barcelona, Spain

**Keywords:** Heart Rate Variability Biofeedback, RSA, Resonance frequency, Deep breathing, Guidelines, Checklist

## Abstract

Heart Rate Variability Biofeedback (HRVB) has been widely used to improve cardiovascular health and well-being. HRVB is based on breathing at an individual’s resonance frequency, which stimulates respiratory sinus arrhythmia (RSA) and the baroreflex. There is, however, no methodological consensus on how to apply HRVB, while details about the protocol used are often not well reported. Thus, the objectives of this systematic review are to describe the different HRVB protocols and detect methodological concerns. PsycINFO, CINALH, Medline and Web of Science were searched between 2000 and April 2021. Data extraction and quality assessment were based on PRISMA guidelines. A total of 143 studies were finally included from any scientific field and any type of sample. Three protocols for HRVB were found: (i) “Optimal RF” (*n* = 37), each participant breathes at their previously detected RF; (ii) “Individual RF” (*n* = 48), each participant follows a biofeedback device that shows the optimal breathing rate based on cardiovascular data in real time, and (iii) “Preset-pace RF” (*n* = 51), all participants breathe at the same rate rate, usually 6 breaths/minute. In addition, we found several methodological differences for applying HRVB in terms of number of weeks, duration of breathing or combination of laboratory and home sessions. Remarkably, almost 2/3 of the studies did not report enough information to replicate the HRVB protocol in terms of breathing duration, inhalation/exhalation ratio, breathing control or body position. Methodological guidelines and a checklist are proposed to enhance the methodological quality of future HRVB studies and increase the information reported.

**Supplementary Information:**

The online version contains supplementary material available at 10.1007/s10484-023-09582-6.

## Introduction

Based on yoga and meditation from ancient Asiatic cultures, slow, deep, and abdominal breathing has become a popular technique to regulate psychophysiological states (Brown & Gerbarg, 2005a, b) or, in other words, to control our mind-body relationship. Nowadays, slow breathing is considered an efficient technique to improve mental and physical well-being (Russo et al., 2017; Zaccaro et al., 2018). However, there is still a lack of consensus about how to regulate the breathing pace, its interventional protocol, and methodological factors.

In 2000, based on previous cardiovascular research, Lehrer et al. (2000) proposed a standardized protocol for increasing the cardiac variability, with the final aim of obtaining both physical and mental benefits: “resonant frequency biofeedback” or “respiratory sinus arrhythmia (RSA) biofeedback” (later called Heart Rate Variability Biofeedback, HRVB). The technique consisted of training people to breathe at their resonant frequency (RF) with the aim of producing maximal increases in amplitude of RSA, defined as the variation in heart rate due to the breath rhythm (Lehrer, 2013). This synchrony between slow breathing and heart rate improves gas exchange and increases oxygenation (Noble & Hochman, 2019; Yasuma & Hayano, 2004; Zaccaro et al., 2018).

When breathing at RF (or near to ~ 6b/m) some changes may occur that may evolve slowly, such as increased activity of the vagus nerve and the parasympathetic system, or increased body and brain oxygenation. All these physiological benefits of breathing at RF could be responsible for the mental benefits of HRVB. These psychophysiological benefits are based on the neural connection between the cardiorespiratory system and the limbic and prefrontal areas through the brain stem.

Last but not least, HRVB increases Heart Rate Variability (HRV; Lehrer & Gevirtz, 2014), which is defined as the fluctuations in the time interval between consecutive beats, since a healthy heart is not a metronome (for a review (Shaffer & Ginsberg, 2017)). HRV is considered an index of autonomic resilience, because it reflects the ability to recover from exposure to both physical and psychological stressors (Hildebrandt et al., 2016; Lehrer, 2018; Walker et al., 2017). In addition, HRV has been demonstrated as a biomarker of psychophysiological and cardiovascular health, diet habits or well-being (Alvares et al., 2016; Appelhans & Luecken, 2006; Chalmers et al., 2014; Young & Benton, 2018). In fact, Mather and Thayer (2018) proposed that high amplitude oscillations in heart rate have positive effects on the cerebral neural networks related to emotional regulation, because high levels of HRV are associated with higher functional connectivity between the amygdala and the medial prefrontal cortex, which they called the *neurovisceral integration model* (Sakaki et al., 2016; Thayer et al., 2012).

Lehrer et al. (2020) reviewed randomized controlled studies using HRVB as an intervention. They concluded that the efficacy of HRVB was mild to moderate for several emotional, physical and performance outcomes like anxiety, anger, or cardiovascular diseases. The authors suggest that HRVB could be considered an excellent complementary intervention for professionals that work in health, medicine, education, and sport fields. In addition, other reviews also concluded that HRVB is a promising intervention for sport performance (Jimenez Morgan & Molina Mora, 2017), stress and anxiety reduction (Goessl et al., 2017), substance use disorders (Leyro et al., 2019), pain management (Reneau, 2020), and some cardiovascular diseases (Gevirtz, 2013; Pinter et al., 2019) among others. HRVB is therefore a promising technique to improve psychophysiological health and well-being.

However, as we are going to discuss in this review, there are three main types of breathing intervention protocols for HRVB, usually under the same terminology of *Heart Rate Variability Biofeedback.* For this review, we classified these protocols as “Optimal RF” (breathing at the previously detected personal RF); “Individual” (a biofeedback device displays the optimal breathing rate on a screen based on real-time cardiovascular data); and “Preset-Pace” (breathing at a standard breathing rate, usually 6 breaths/minute). Thus, even though a standard protocol was published in 2000 (Lehrer et al., 2000) and the same authors updated it a few years later (Lehrer et al., 2013), there is still a lack of a *gold standard* for HRVB interventions. In addition, most of the published studies applying HRVB did not report enough methodological information to allow replication, mostly related to breathing control like the inhalation and exhalation rate and control variables like body position during the HRVB intervention.

One of the fundamental pillars in science is replication, which consists in re-examining research findings independently to increase their validity (Schmidt, 2009). Replication is important because it generates scientific knowledge, allows unhelpful or even harmful interventions to be avoided, and provides robust evidence (Michie et al., 2009). However, replication is still a major concern for science (Fanelli, 2018). For example, around 1,400 researchers (90%) responded to a survey in *Nature* that there is a replication crisis in science (Baker & Peny, 2016). And, in psychological science, replication is suggested to be between 36 and 77% (*depending on how you assess replication*) (Open Science, 2015; Patil et al., 2016). Whatever the percentage is, these results show that science should improve its replication levels. There are several threats to replication like publication bias, *p*-hacking, low statistical power and so on (Munafo et al., 2017), but also poorly detailed written protocol information, inadequate methodological reporting, and inadequate designs (Glasziou et al., 2014; Ioannidis et al., 2014) that, as we found in this review, are major concerns for HRVB studies.

Consequently, this deficit in methodological quality could explain the lack of robustness in the efficiency of HRVB interventions as found in previous systematic reviews and meta-analyses (*see above*). Improving methodological quality, study designs and intervention protocols is thus an urgent need for future HRVB research studies and clinical interventions. Therefore, the aim of this review is to create a picture of the published HRVB protocols, critically evaluate the quality of the application of HRVB and propose guidelines and a checklist for future HRVB studies as already proposed for HRV (Catai et al., 2020; Laborde et al., 2017; Quintana et al., 2016). For example, in a methodological review, Laborde et al. (2017) provided recommendations for the assessment of HRV in psychological research, from study design and data analysis, like artifact corrections, to the structure of the experiment (*what they called the three Rs: resting, reactivity and recovery*) and confounding variables such as age, medication, or sleeping routines. In detail, we aim to describe the designs and methods of the published HRVB interventions, evaluate the quality of the studies and remark on the aspects that should be improved to increase both the scientific quality and the clinical applicability of future HRVB interventions. Finally, and based on the previous findings, we aim to propose several methodological items, in guideline and checklist format, to improve future HRVB studies and interventions at the methodological level.

This review thus complements the previous ones, which analyzed the efficiency of HRVB (*see above*), by evaluating the quality of the HRVB interventions and proposing *methodological guidelines* to enhance future HRVB interventions and, by extension, the replication of HRVB protocols by proposing a *reporting checklist*.

## Methods

This systematic review was carried out according to the “Preferred Reporting Items for Systematic Reviews and Meta-Analyses Guidelines” (PRISMA) (Moher et al., 2009), and its protocol was registered previously in PROSPERO: CRD42018086748 (Lalanza et al., 2018).

### Search Procedure

**Fig. 1:**
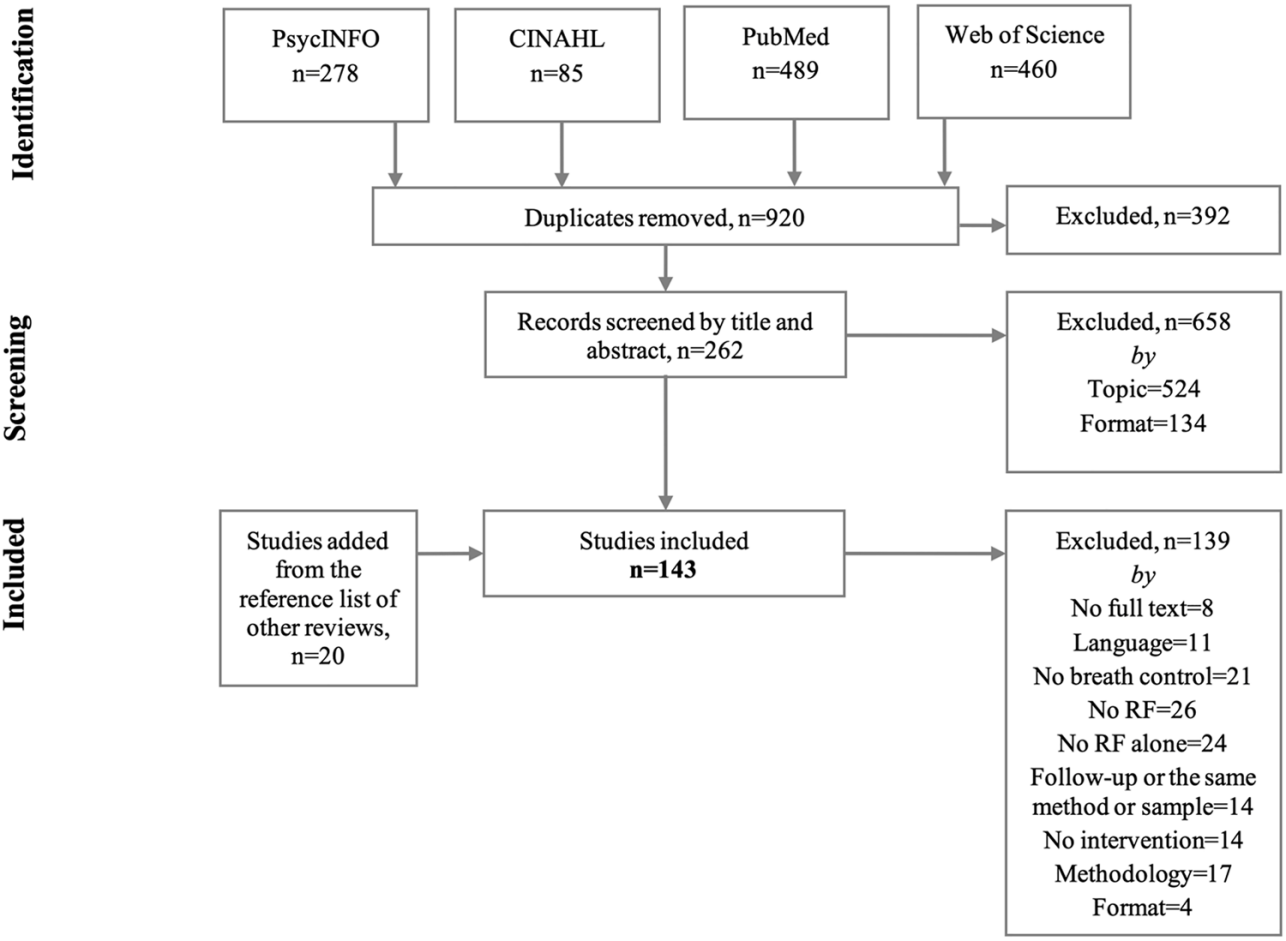
Flowchart of the different phases of the searching and selection of studies, following the guidelines of the Preferred Reporting Items for Systematic Reviews and Meta-Analyses (PRISMA). *RF* resonance frequency. *See table for details on the exclusion criteria*

As the aim of this systematic review was about the different methodologies applied in HRVB interventions, a search was made for all peer reviewed HRVB studies without considering the field of the intervention. The search was performed in April 2021 (Fig. 1). The following electronic bibliographic databases were checked from the following portals: PsycINFO by PsycNET and OVID, CINAHL by EBSCOhost, MEDLINE by PubMed, and Core Collection of Web of Science by Web of Science. The search strategy followed the Peer Review of Electronic Search Strategies (PRESS) guidelines recommendations (McGowan et al., 2016). The general searching syntax was: *(“resonance frequency” and breathing) or (“resonant frequency” and breathing) or (“resonance frequency” and biofeedback) or (“resonant frequency” and biofeedback) or (“resonance frequency” and training) or (“resonant frequency” and training) or (“heart rate variability” and biofeedback) or (HRV and Biofeedback) or “heart rhythm coherence” or HRVB*. In addition, the following filters were applied, if possible: (i) from 2000 (*when the first guidelines of HRVB were published)* to 2021; (ii) human participants; (iii) empirical, experimental and clinical studies, (iv) scientific fields related to psychology, medicine, neuroscience, etc., and (v) language: English, Catalan and Spanish. The specific search syntaxes are provided in supplementary Tables 1–4. When the paper’s full text was not available, the authors of the study were contacted to request the full text.

Studies were stored, and duplicates were deleted by Mendeley. Then, one review author (JFL) selected the studies based on the eligibility criteria of its title and abstract. After that, the same author checked and included, if necessary, those studies being cited in the reference list of previously published reviews of HRVB. Finally, the selected papers (in full text) were checked and included by two pairs of review authors independently (JFL/SL/RB/CG) (Fig. 1). Discrepancies were resolved through discussion (with a third author, LC, when necessary) until reaching consensus.

### Eligibility Criteria

Experimental and quasi-experimental studies were selected if they applied an active HRVB intervention without another simultaneous experimental intervention (Table 1). Thus, studies indicating only deep breathing, combining different techniques at the same time, and other types of methodologies (single-cases, reviews, meeting abstracts, book reviews, protocols, etc.) were discarded. Selected papers had to be written in English, Catalan, or Spanish.

**Table 1:** Exclusion criteria of articles after obtaining the full text

Type of Exclusion	Exclusion definition
No breath control	Breathing was not considered either a factor or a treatment.
No RF	RF was not detected individually before treatment for each participant.
*and*	RF was not pre-set for all participants.
*and*	RF was not detected individually during each treatment session. In this case, RF, Coherence or RSA had to be indicated as the main feature of the breathing treatment in order to be considered HRVB and be included in this systematic review.
No RF alone	HRVB had to be carried out alone, without any other simultaneous treatment; except for clinical samples with a clinical treatment, for example, patients after a cardiac surgery. Intervention with different treatments, including HRVB, administered sequentially has been included.
Follow-up a previous included study or the same method/sample	Follow-up studies of a previously included study have not been included in this systematic review. Studies that unquestionably applied a secondary analysis of a previous published method and/or sample were also excluded.
No intervention	HRVB had not been applied to obtain a benefit to the participants; for example, creating a computerized model of RSA was not an included study.
Methodology	Methodologies that were not: experiments or quasi-experiments; for example, reviews not excluded by title and abstract in the first screening phase.

*HRVB* Heart Rate Variability Biofeedback, *RF*, Resonance (or Resonant) Frequency

**Table 2:** HRVB intervention and reporting quality checklist

HRVB methodological guidelines	Considered	Reported
(0) Pre-Registration or Registration		
(1) Sample		
Power analysis: calculation of the minimum sample size to obtain an appropriate effect size.		
Biological sex: number of women and men per group		
Age: mean age for each group		
Condition: e.g. students, cardiovascular patients or suffering from obesity		
Weight (*optional*): mean weight for each group		
Height (*optional*): mean height for each group		
(2) Allocation		
Recruitment of participants/patients/clients to the study		
Compensation for participating in the study (payment, credits, holidays, etc.)		
Inclusion and exclusion criteria assessed and explained		
Awarenessor suspicion of participants of the experimental group assignation		
Random (*optional*): assignation		
(3) Missing participants and missing data		
Initial sample: once the participants have agreed to participate or come to the first session		
Final sample: participants included for the data analysis		
Causes for losing participants/patients (*optionally use a diagram)*		
Missing data for the statistical analysis		
(4) Breathing protocol		
Type of HRVB intervention based on the category of breathing technique. *The type of intervention determines which items need to be completed*		
Resonance Frequency detection (*optional*): details about the establishment of the individual resonance frequency (RF). This item also includes when and how many times the RF was detected across the entire HRVB intervention		
Mean of the Resonance Frequency (*optional*) for the experimental group		
“Preset or fixed” Resonance Frequency (*optional*): for the “preset-pace” or slow-breathing intervention		
Control of breathing rate (*optional*): for intervention with a collective or individual resonance frequency. E.g. use of a respirometer		
Inhalation, holding and exhalation seconds for each breathing cycle		
(5) Breathing intervention		
Number of laboratory sessions of the HRVB intervention		
Minutes at the laboratory actually breathing (not doing other experimental tasks)		
Number of home-practice sessions (*optional*)		
Minutes per each home-practice session (*optional*)		
Control assessment for the home-practice sessions (*optional*)		
(6) Conditions of the intervention		
Laboratory conditions: ambient light		
Laboratory conditions: temperature of the room		
Laboratory conditions: body position		
Laboratory conditions: time of day		
Laboratory conditions: number of participants at the same time		
Laboratory conditions (*optional*): smell/aroma, humidity and eyes (closed or open)		
Home-practice sessions (*optional*): like time of day, ambient conditions, etc.		
Recommendations for participants: like caffeine or alcohol intake, minimum hours of sleep, exercise before the session, etc.		
Control of the recommendations for participants: how to assess the accomplishment of the previous recommendations and what to do if a participant does not follow them		
(7) Equipment		
HRVB apparatus: brand, type of metronome, type of biofeedback, is it validated? is it invasive? etc.		
HRV apparatus (*optional*): brand, type of metronome, type of biofeedback, is it validated? is it invasive? etc. for the analysis of heart rate variability		
Other apparatus for physiological analysis that can interact with the breathing performance such as a thoracic belt for breathing analysis (e.g. respirometer)		

Mark with and X whether you have considered or think about the item before applying the HRVB intervention. Mark with and X whether you have reported each item in the manuscript giving the necessary details

### Data Extraction

Data were extracted by an ad hoc checklist, which included data about sample, study design, HRVB protocol and environmental conditions, and risk of bias. Similarly to the protocol for search procedures, two independent pairs of review authors (JFL/SL/RB/CG) extracted the data of each final selected study and discrepancies were resolved through discussion.

### Quality Assessment

The risk of bias of the included experimental (*random*) studies was assessed (by JFL/SL/RB/CG) with the “Cochrane Risk of Bias Tool for Randomised Trials” (Higgins et al., 2011), and for the quasi-experiments (*non-randomised studies*) with the “Risk Of Bias In Non-randomised Studies - of Interventions” (ROBINS-I; (Sterne et al., 2016)). Domains regarding the outcomes or reports of the studies were not applied due to the methodological nature of this systematic review. The 5-Score system of the ROBINS-I was reformulated using the terminology of Cochrane, and reduced to 3 categories, merging Moderate and Serious Risk of Bias into a “Medium Risk” category, to simplify its interpretation and assessment.

### Statistical Analysis

Descriptive analyses of the findings from the included studies were structured around the research design, type of breathing intervention, relevant variables measured in the HRVB protocols, number of sessions, minutes of breathing, type of control group, biological sex and so on. Agreement between reviewers during the study selection process was analyzed by Cohen’s Kappa (Cohen, 1960).

## Results and Discussion

### Identified Studies

From the 920 non-duplicate studies found and screened by title and abstract, 262 were selected after excluding for topic (*n* = 524) and methodology (*n* = 134) (Fig. 1). Of these, 19 were excluded owing to non-availability of full text (*n* = 8) and language (*n* = 11), and 20 studies were added from the reference list of other reviews related to HRVB. Thus, 282 studies were assessed for the full text and 143 were finally included in the review (Fig. 1). Percentage of agreement between the reviewers during the study selection at first round was 85.8% (*Kappa* = 0.72; CI95%: 0.63 to 0.80).

Two of the included studies performed two types of HRVB interventions separately (Chalaye et al., 2009; Chen et al., 2016), and one study (Kennedy & Pretorius, 2008) applied the same type of HRVB in two experimental designs. Therefore, depending on the variable analyzed, the sample (n) will range from 143 to 145 interventions from a total of 143 studies.

### A Summary of the HRVB Interventions

As the first objective of this review was to summarize the different protocols and methodologies in HRVB, we summarized the designs, methods and protocols of the studies applying an HRVB intervention. For each intervention (*n* = 145), the sample and experimental design are shown in supplementary Tables 5, the protocol of each HRVB intervention in supplementary Tables 6 and the general laboratory or clinical conditions in supplementary Table 7.

#### Types of HRVB Intervention

**Fig. 2:**
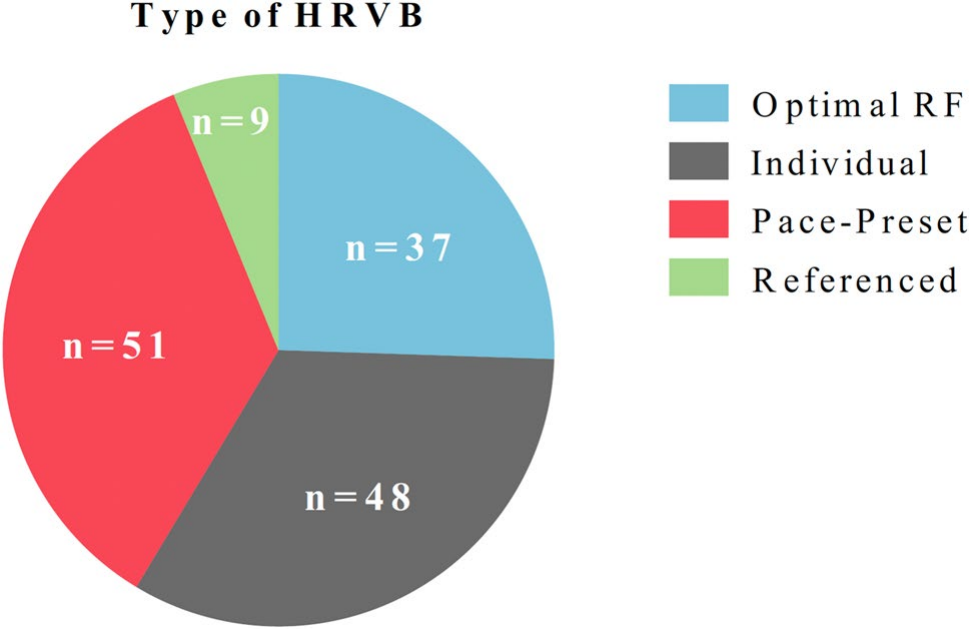
Type of HRVB intervention (*n* = 145). *Optimal RF*: interventions that followed the original protocol by Lehrer et al. (2000) and applied a range of breathing rates before each HRVB session or for the total HRVB intervention in order to detect the optimal resonance frequency (RF) for each participant. *Individual*: those interventions that used a biofeedback system that indicates the actual breathing rhythm and the optimal breathing rhythm in real time for each participant. Thereby, each participant individually adapted the breathing rate to the optimal rhythm or rate indicated by the biofeedback system. *Preset-Pace*: a fixed breathing ratio is set up for all participants and all HRVB sessions, usually 6 b/m. *Referenced*: those studies that did not report enough information to determine the type of HRVB intervention, because the authors referred to an HRVB intervention from a previous study

Based on the type of breathing intervention, we classified the HRVB interventions found in the included studies into four different categories that we named: Optimal RF (*n* = 37), Individual (*n* = 48), Preset-Pace (*n* = 51) and Referenced (*n* = 9) (Fig. 2). We have already commented on the first three categories in the Introduction of this review, but in addition, we have added the special category “Referenced” when the studies did not provide enough information to classify them in one of the three previous categories of HRVB.

A standardized version of HRVB was proposed in 2000 for clinical and professional applications (Lehrer et al., 2000) and an updated and shorter version was published by the same authors thirteen years later (Lehrer et al., 2013). The studies that followed these two protocols were assigned to the “Optimal RF” category, which included almost 1/4 of the studies (e.g., Caldwell & Steffen, 2018; Hallman et al., 2011). The typical “Optimal RF” protocol starts with breathing practice following a pacer or a metronome and then detection of the RF. In the “Optimal RF” protocol a range of breathing rates is applied before each HRVB session or before the total HRVB intervention to detect the optimal resonance frequency (RF) for each participant. During the second session the participant/patient continues to practice the RF and learns the abdominal and pursed lips breathing to achieve the RF without hyperventilating. In the third session, the therapist must continue with the breathing technique training and introduce the home training device. And, finally, sessions 4 to 10 proceed with breathing at the individual RF. The proposed short version kept the original training structure and just reduced the “intervention” sessions. Both versions (short and long) were based on the same theoretical principle, in which the best breathing rhythm is unique for each person, the RF.

To find the RF, participants/patients breathe at different rates, usually from 6.5 to 4.5 b/m for 2 min each. Then, the HRVB device calculates the RF and determines which of the breathing rates is optimal for each participant/patient. Finally, participants/patients usually breathe for 5 to 20 min daily (or twice a day) at their individual RF.

A second type of intervention, named “Individual” for this review, was applied in 33% of the studies (e.g., Narita et al., 2018; Stern et al., 2014). In these studies, the participants/patients receive a special device that monitors and displays their heart rate on a screen. They are instructed to make the heart rate go up as much as possible during inhalation and down as much as possible during exhalation. Instructions may also include relaxed breathing to amplify the magnitude of the heart rate. One of the advantages of this intervention is that it skips the initial procedure of finding the RF, which the participant/patients could eventually find by matching the heart rate curve during training. However, its major disadvantage is that the users may not know how to optimally pace their breathing without the device. As happens with “Optimal RF”, the “Individual” intervention requires specialized software and hardware. Today, mHealth has democratized this technology, making it accessible to different types of users, from medical personnel to sports trainers. For instance, the increasing popularity of apps and wearables has made it possible for a wide range of users to access their cardiovascular data at a relatively low cost. However, further research is needed to confirm the reliability of these mobile apps in certain conditions, such as abnormal sinus arrhythmia or atrial fibrillation (Li et al., 2019).

The faster, easier and more economical alternative for applying HRVB is establishing a preset breathing rate for all the participants that is usually set at 6b/m or 5.5b/m (Lehrer & Gevirtz, 2014; Lin et al., 2014). We named this type of HRVB intervention “Preset-Pace” and it was applied in approximately 1/3 of the included studies (e.g., Bartur et al., 2014; Nolan et al., 2005). Although a preset breath ratio is not individually calculated in the same way as RF, we included this type of intervention because it is based on the theoretical principle of HRVB.

Finally, 4.4% of the studies (e.g., Meyer et al., 2018; Ozier & Linden, 2018) did not provide enough information to classify them into one of the three previous categories of HRVB. Thus, we created a special category named “Referenced”, because in these studies the authors just mentioned the previous scientific work on which the HRVB was based (*in all cases, the work of Lehrer and colleagues was cited*). We strongly recommend providing the basic information about the HRVB protocol and avoiding just citing a previous study. For example, as an alternative to just citing a previous study, we recommend adding the methodological details of the HRVB intervention in the “supplementary information” section in order to keep the study short, direct and easily readable without losing relevant methodological information.

#### Characteristics of HRVB Interventions

**Fig. 3:**
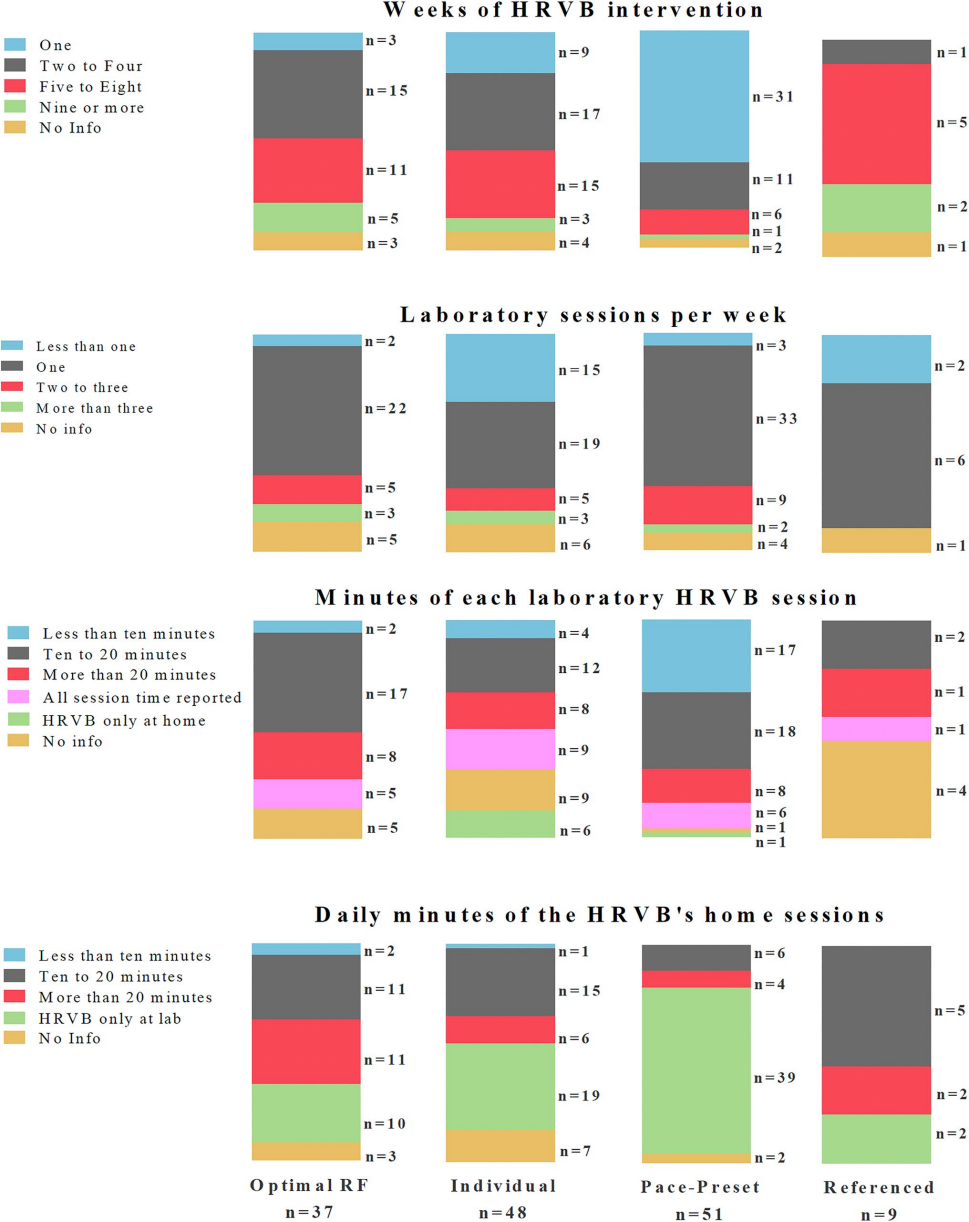
Characteristics of the HRVB intervention by type of HRVB intervention. Based on the four categories of HRVB interventions (columns for: Optimal RF, Individual, Preset-Pace, and Referenced), we indicated in rows: (1) the total duration of the HRVB intervention in weeks; (2) the number of laboratory sessions per week; (3) minutes of exclusively breathing during laboratory HRVB sessions (in green those studies that only applied home sessions, in purple those studies that only reported the duration of the entire session, but not the breathing time specifically); (4) daily minutes of exclusively breathing during the home HRVB sessions (in green those studies that only applied laboratory sessions). The numbers (n) in the bars indicate the number of studies/interventions per each category

Once we had ascertained that most of the HRVB studies applied “Optimal RF”, “Individual” or “Preset-Pace” interventions, the next step for a current and accurate picture of HRVB applicability was to detect protocol differences like differences in the number of sessions. These variables are shown in Fig. 3 and commented below.

The total number of weeks of HRVB intervention is shown in the first row of Fig. 3. Surprisingly, whereas most “Optimal RF” and “Individual” interventions lasted from two to eight weeks (e.g., Hasuo et al., 2018; Yucha et al., 2005), most of the “Preset-Pace” interventions lasted only one week (e.g., Stromberg et al., 2015; Wells et al., 2012). This is an unexpected result, because the advantage of not relying on technology provided by the “Preset-Pace” is not applied in long interventions combined with home sessions. On the other hand, those studies that applied a more complex biofeedback technology like “Optimal RF” and “Individual” designed longer interventions that included home sessions (*see below*). For “Referenced” studies, most of them designed interventions from five to eight weeks. As most of the “Referenced” studies cited the standardized HRVB protocols, this is an expected result. Finally, ten out of 145 interventions did not provide enough information in the paper to know the duration of the HRVB intervention, which makes it impossible to replicate them.

The amount of laboratory sessions per week is shown in the second row of the Fig. 3. This variable is relevant, because laboratory sessions are the only type of session that allows scientists/therapists to control the breathing performance of the participants directly. Nevertheless, as shown in the next sections, the control of laboratory variables was also inadequate in several studies. Among “Optimal RF” studies, most applied only one laboratory session per week (e.g., Hassett et al., 2007; Sutarto et al., 2012) and among “Individual,” most applied one or less than one laboratory session (e.g., Wu et al., 2012; Zucker et al., 2009). Therefore, these studies were usually designed with one laboratory session per week to teach and control the adequate breathing technique and most of them included daily home sessions (*data do not shown*) using the same breathing rate detected during the laboratory session (e.g., Brinkmann et al., 2020; Tan et al., 2011). “Preset-Pace” studies mainly applied one laboratory session per week. The majority of “Preset-Pace” studies lasted one week which means that most of the “Preset-Pace” studies were acute interventions with only one breathing session (e.g., Francis et al., 2016; MacKinnon et al., 2013). Again, a significant number of studies did not report enough information to know the amount of laboratory sessions per week with certainty, because 16 out of 145 interventions did not explain it in the published paper.

The minutes of laboratory and home breathing are shown in the third and fourth rows of Fig. 3. In general, participants/patients breathed for 10–20 min in each HRVB session. In detail, for “Optimal RF” and “Preset-Pace”, laboratory sessions usually lasted ten to 20 min (e.g., Eddie et al., 2018; Schmidt et al., 2012). For “Individual” interventions however there was not a clear pattern. Among the interventions that applied laboratory sessions (*n* = 138), 19 of them did not report the minutes of breathing, and 21 only reported the duration of the entire laboratory session but not the detailed minutes of breathing. Regarding the minutes of home sessions, ten to 20 min of breathing were again the preferred option (e.g., Reiner, 2008; van der Zwan et al., 2015), even though for “Optimal RF,” 1/3 of interventions applied more than 20 min (e.g., Lehrer et al., 2003; Sutarto et al., 2013). For “Preset-Pace”, it is confirmed that most of the studies only applied laboratory sessions, because only twelve interventions applied home sessions from ten to 20 min or more (e.g., Chen et al., 2016; Wang et al., 2010). Finally, twelve out of 75 interventions (that applied home sessions) did not report this information appropriately.

Therefore, the general protocol for HRVB interventions (“Optimal RF” and “Individual”) lasts from two to eight weeks, has one laboratory session per week while participants practice HRVB daily at home for 20 min. On the other hand, “Preset-Pace” studies were mostly planned as acute interventions of only one session in the laboratory.

### Design

For studies and interventions applying HRVB, the category of breathing technique is the central methodological aspect. However, the study design, the sample and the control group are other relevant methodological information that cannot be neglected. In this section, the information is not divided by the category of breathing, because the study design is not a determinant factor for core HRVB variables such the calculation of the RF. The purpose of this section is therefore aimed to achieve the first objective of this review, describe the methodological characteristics of the HRVB studies.

#### Study Design

**Fig. 4:**
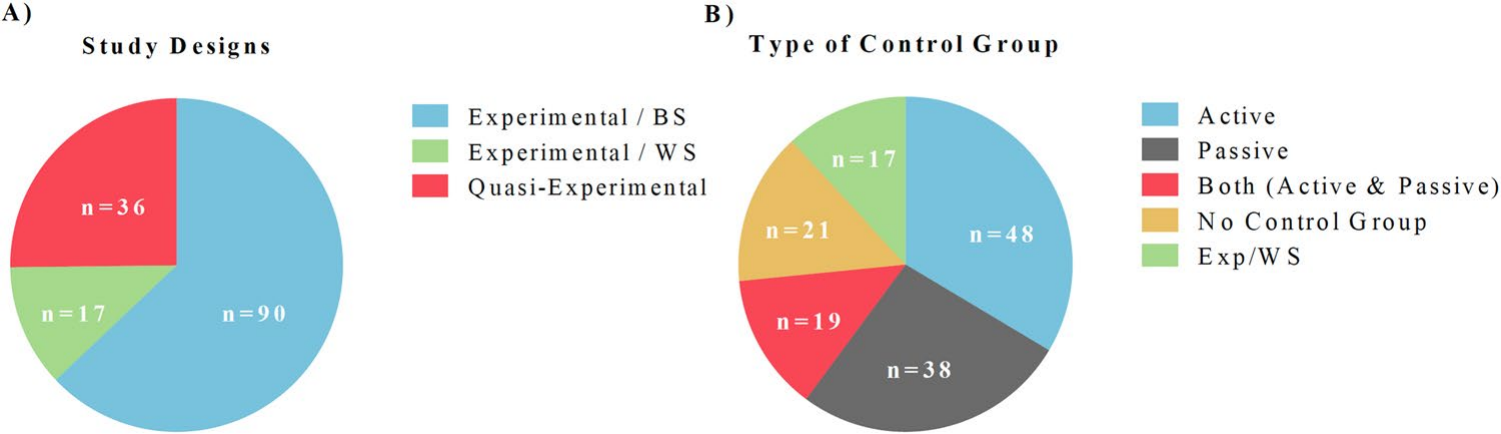
Design and control group of the included studies and interventions (*n* = 143). **A** Study designs: *Experimental/BS (between-subjects)*: studies that applied an experimental (randomized and/or equivalent control group) between-subjects design (each participant was assigned to one experimental group). *Experimental/WS (within-subjects)*: those studies that applied an experimental within-subjects design (each participant was assigned to one order of the experimental groups). *Quasi-Experimental*: those studies that applied a quasi-experimental design (non-randomized and/or non-equivalent control group). **B** The type of control group/s or condition/s. *Active*: participants did something active such as breathing without a biofeedback screen, writing in a diary or running. *Passive*: participants did not do anything active, they were on a waiting list or sat in the laboratory. Studies that only reported a control group were included in this category. *Both*: studies that included two or more control groups/conditions and at least one was active, and one was passive. *No control group*: this category included quasi-experimental studies that did not have a control group. *Exp/WS (within-subjects)*: studies in which all participants received both the control and the experimental conditions. The numbers (n) in the graphs indicate the number of studies per each category

A basic classification of interventional studies is based on the type of study design or experimental approach (Fig. 4A). Among the included studies and interventions (*n* = 145), randomized experiments were most common (75%). They were divided into between-subjects designs (84%), where each participant was randomly assigned to each experimental condition, and within-subjects designs (16%), where each participant was randomly assigned to each order of presentation of the experimental conditions. The second-most-common type of study design found in this review was the quasi-experimental design (25%), which was defined as a study aimed to demonstrate causality between the intervention and the final outcome, but that did not use randomization or did not include an equivalent control group.

#### Type of Control Group

The type of study design was partially determined by the type of control group. Control groups are a key element of science, because they allow researchers to adequately compare and evaluate the experimental intervention or treatment and obtain valid conclusions. Ideally, an experimental study that uses HRVB as a treatment should include two control groups. The first one must be an “active” control group that for example breathes freely, reads a book about stress management or practices sport. Thereby, one can compare the HRVB directly to another treatment. The second control group must be a “passive” one that for example goes to the laboratory room and sits quietly or is part of a waiting list. One can then control the effects of going to the laboratory and contextual events such as meteorological changes that could affect the mood of the participants. We only found 19 studies that included both passive and active control groups (e.g., Lee et al., 2015; Vagedes et al., 2019) (Fig. 4B). Thus, we recommend including both types of control groups in future HRVB studies, even though the passive group was just a “waiting list”. Among those studies that only included one control group, 48 included an active control (e.g., Li et al., 2015; Munafo et al., 2016), whereas 38 included a passive control (e.g., Cullins et al., 2013; Meyer et al., 2018). We did not include the within-subjects studies in this category, because the same participants received the experimental and control conditions. Finally, 21 studies did not include a control group. Due to the nature of these studies, it can be difficult to include a control group. All 21 studies were quasi-experimental designs, of which 19 included clinical patients or participants related to elite sport (e.g., Ozier & Linden, 2018; Reneau, 2020; Shaw et al., 2012). Including a non-treated condition, an equivalent healthy group or a within-factor design could be impractical or even unethical in these sorts of studies. On the other hand, we encourage the inclusion of a control group and the design of a randomized experiment if the characteristics of the sample allow it, for example in studies with students or with healthy adults.

#### Sample and Allocation

**Fig. 5:**
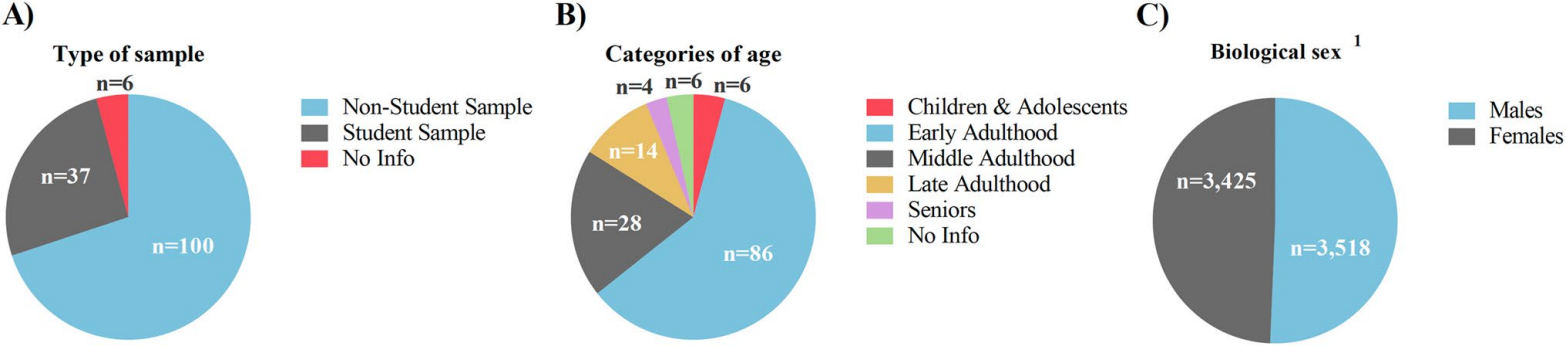
Characteristics of the samples used in the included studies and experiments (*n *= 143). **A** Type of sample divided between those studies that included students and those that did not include students. **B** Age of the samples based on the reported “mean age” or “age range”. *Children & Adolescents*: less than 18 years, *Early Adulthood*: between 18 and 39 years, *Middle Adulthood*: between 40 and 55 years, *Late Adulthood*: between 56 and 65 years, *Seniors*: more than 65 years. **C** Total males and females considering the final sample of each study. The numbers (n) in the graphs indicate the number of studies per each category. ^1^Four studies did not report the biological sex of the participants (*n* = 139)

The type of sample is also a relevant factor, not only for interpretation and extrapolation but also for replication of scientific studies. The use of university student participants is a common research practice, but could hamper the generalization of the results to the general public (Hanel & Vione, 2016). Considering the translational character of HRVB, we wanted to assess the number of studies using university students. Fortunately, we did not find that most studies used university students, rather the contrary (Fig. 5A). In fact, samples composed by university students were found in only 26% of the studies (e.g., Tavares et al., 2017; Zunhammer et al., 2013), whereas non-student samples were used in 70% (*n* = 100) (e.g., Narita et al., 2018; Yu et al., 2018). Unfortunately, six studies did not adequately report the type of sample, which is a major methodological concern.

“Age” is another factor that interacts with cardiovascular parameters, being inversely related to HRV (Voss et al., 2012). However, most of the studies applying an HRVB intervention recruited young adults (Fig. 5B). In addition, we found several ways to report the age of the participants, from a mean per each experimental group to a simple range. We recommend reporting the mean and standard deviation of the age for each experimental condition and for the total sample as it is the most complete option. In this review, for studies indicating a range, we took the middle value, and for studies indicating the mean of age per group, we calculated the mean of the groups (as usually the number of participants is very similar among experimental and control groups, even though it should be considered as an approximate value). After that, due to the high variability of reporting formats and dispersion, we classified participants into five age categories: childhood and adolescence (< 18 years), early adulthood (18–39 years), middle adulthood (40–55 years), late adulthood (56–65 years) and seniors (> 65 years). As shown in Fig. 5B, most of the studies (60%) included participants in early adulthood (e.g., Lehrer et al., 2018; Russell et al., 2017). In the second position, we found middle adulthood (20%) (e.g., Deschodt-Arsac et al., 2020; Windthorst et al., 2017) and with less than 10% of the studies for each category, we found participants in late adulthood (e.g., Weeks et al., 2015; Zauszniewski et al., 2013), children and adolescents (e.g., Sierra Murguía et al., 2017; Sowder et al., 2010) and seniors (e.g., Del Pozo et al., 2004; Jester et al., 2019). Therefore, and always depending on the aim of the study, we encourage researchers to include other age groups rather than young adults. Sadly, six studies did not report the age of the participants, which should be avoided in future studies.

Finally, biological sex is still a major issue in science, mainly in basic and human or animal studies (Beery & Zucker, 2011; Mamlouk et al., 2020). Based on the false myth that the oestrus cycle adds too much variability, most human or animal studies include only males (Becker et al., 2016). Luckily, this sex bias is disappearing in biological science and there is a significant increase in the last 10 years in the inclusion of both sexes (Woitowich et al., 2020), but women are still a minority in randomized controlled trials (Geller et al., 2018). Regarding HRVB, we found that the total sample of the included studies was almost 50% women and 50% men (Fig. 5C). Unfortunately, four studies did not report the biological sex of the participants.

### Lack of Methodological Information

**Fig. 6:**
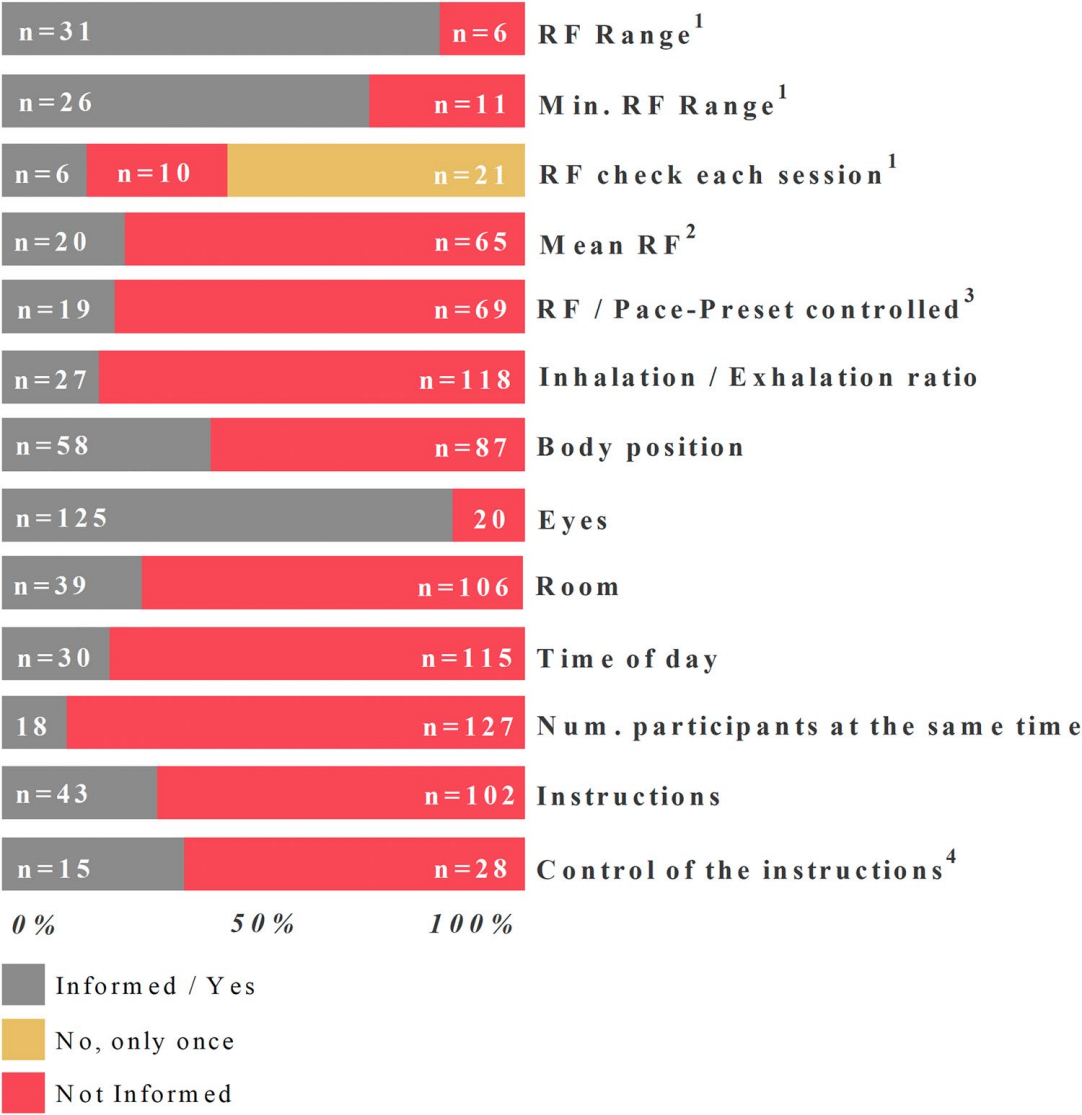
Methodological features and co-variables reported in the selected papers. *RF Range*: the breathing rates or range applied to detect the optimal resonance frequency (RF). *Minutes RF range*: the minutes of each breathing rate from the “RF Range”. *Check RF each Ses.*: the optimal RF was assessed before each HRVB session; “*yes” instead of “reported”*. *Mean RF*: the mean of the RF in the total sample. *RF/Preset-Pace controlled*: any attempt to ensure that participants/patients breathed at the indicated RF or preset breathing rate. *Inhalation/Exhalation Ratio*: the ratio of inhalation, hold and exhalation during breathing. *Body Position*: the body position during the HRVB sessions. *Eyes*: open or closed eyes during the HRVB intervention. In studies that used a screen or a display, “open eyes” are inferred. *Room*: room conditions including: light, noise, smell and temperature; it was considered “reported” if just one condition was reported. *Time of day*: the time of day when the HRVB sessions were performed. *Num. participants at the same time*: how many participants, clients or patients were together during an HRVB session. *Instructions*: recommendations given to for participants before each HRVB session, like no smoking 3 h before the session. *Control of the instructions*: it was considered “reported” if just one of the instructions were reported in the study. *n* = 145, except for: ^1^only for “Optimal RF”, *n* = 37; ^2^only for “Optimal RF” and “Individual”, *n* = 85; ^3^only for “Optimal RF” and “Preset-Pace”, *n* = 88; ^4^only for those studies that reported any pre-HRVB instruction, *n* = 43. The numbers (n) in the graphs indicate the number of studies per each category

The second aim of the review is to critically evaluate the quality of the studies and highlight methodological aspects that could be improved in future HRVB studies. Hence, Fig. 6 shows the number of studies that reported essential information about the HRVB intervention and the contextual factors.

In general, we found that many central variables and methodological factors were not adequately reported in the included studies, therefore one should be careful interpreting and comparing some results. A previous systematic review and meta-analysis, for example, also found that it is important to consider methodological quality when interpreting HRV results (Alvares et al., 2016). It could indeed be hypothesized that this lack of methodological information could exemplify the low levels of replication in psychology (Open Science, 2015) and biomedical disciplines (Begley & Ellis, 2012; Ioannidis et al., 2009). These authors (Open Science, 2015) did not conclude that the original evidence was a false positive but suggested that a confluence of factors like poor research designs or publication bias could explain the difficulty in replicating the results. In fact, the National Institutions of Health (NIH) together with the Nature Publishing Group emphasized the need to increase transparent reporting of key methodological information (among other principles), like clearly stating the inclusion/exclusion criteria, in order to improve rigor and replication (NIH, 2017). Munafò et al. (2017) also recently published a manifesto that encourages scientists to improve the transparency, replication and efficiency of scientific research. One of the proposals is indeed improving the quality of the reporting by, for example, reporting experimental conditions and following guidelines (e.g., the Consolidated Standards of Reporting Trials –CONSORT–) may increase the quality of reports of randomized controlled trials, (Plint et al., 2006). Another review also reported the need to improve the reporting of protocols and documentation about research, suggesting that making the full protocols available or even publishing them in advance, e.g. pre-registration, was necessary (Ioannidis et al., 2014). Finally, Quintana et al. (2016) proposed guidelines for reporting articles on psychiatry and heart rate variability (GRAPH). Although the GRAPH is geared towards HRV and psychiatric disorders, these authors also focus on the relevance of increasing methodological details. They elegantly argue that the lack of methodological details: (i) delays the peer-review process, (ii) hinders replication, and (iii) blocks future meta-analysis. We totally agree with them and also defend the need for increasing methodological details, as is shown in the guidelines and checklist we proposed for HRVB.

#### Breathing Information

The first six rows of Fig. 6 include methodological information related to the HRVB intervention. Unlike the previous section, herein we are going deep into the breathing process with detailed information about each of the HRVB interventions.

Beginning with “Optimal RF” interventions, a large majority of studies (84%) (e.g., Hallman et al., 2011; Schuman & Killian, 2019) reported the range of breathing rates (RF range) applied before starting the main HRVB. This initial battery of breathing rates allows detection of the optimal RF for each individual and consequently attainment of the maximal benefits of the HRVB intervention. This initial battery of breathing rates is the key aspect of “Optimal RF” HRVB, so it is surprising that 16% (*n* = 6) did not report this information.

To go into more detail with the *RF range*, the standard duration of each breathing rate trial period is two minutes (according to Lehrer et al., 2000). As this review did not aim to evaluate the efficacy of each HRVB protocol, but rather its quality, we are not going to assess the number of minutes of each study, only whether this information is reported. Most studies reported the minutes of *RF range* (70%) (e.g., Hasuo et al., 2019; Steffen et al., 2017). However, 1/3 of the “Optimal RF” studies did not contain information about the number of minutes of each breathing rate during the *RF range*.

Finally, one can apply the detection of the optimal RF (*RF range*) before each HRVB session, once per week or only once at the beginning of the HRVB intervention. Whereas more than half of the “Optimal RF” studies only applied the *RF range* during the first HRVB session (*n* = 21) (e.g., Rusciano et al., 2017), only six studies checked the optimal RF before each HRVB session or more than once (e.g., Gross et al., 2016; Perez-Gaido et al., 2021). Lin et al. (2012) found that the optimal RF (assessed by the RF mean of participants) changed from session to session (from 6 to 5 b/m) in students (~ 22 year) with prehypertension, whereas Hallman et al. (2011) found that the optimal RF was stable during ten HRVB sessions in older participants (~ 40 year) without cardiovascular disorders. In an experimental laboratory study, Capdevila et al. (2021) have also found that the optimal RF showed some variation in a test-retest protocol. In addition, cardiovascular improvement was also detected during the optimal RF. Similarly, an included study also compared the optimal RF with the optimal RF + 1 breathing rate and corroborated that the non-optimal RF had less cardiovascular and psychological benefits (Steffen et al., 2017). However, the differences in breathing protocols, experimental designs and contextual variables among these studies make it difficult to obtain a clear conclusion about the instability of RF. On the other hand, there is also evidence for the stability of RF. For example, Fisher and Lehrer (2022) found that RF showed a high level of stability, comparing a new “sliding protocol” to the traditional “stepped method”. This new protocol is characterized by a constant change in pace (a fixed rate change of 67.04ms per breath) at each of 78 breathing cycles ranging from 4.25 to 6.75 breaths per minute. Therefore, the stability of RF during the HRVB intervention is still a topic of debate. For future “optimal RF” interventions, we recommend following a published protocol, either a “stepped method” (e.g., Lehrer et al., 2013) or a “sliding protocol” (Fisher & Lehrer, 2022). However, for interventions where available time is not a constraining factor, we suggest checking the optimal RF more often, to both: (i) be sure that participants/patients breathe at a comfortable rate, and (ii) increase the evidence about the stability of RF.

Having detected the optimal RF for each participant/patient, the *mean* RF of the sample is a useful data point for future HRVB studies. For example, “Preset-Pace” studies could choose a more accurate breathing rate depending on the type of sample or “Optimal RF” studies could adjust the *RF range*. For instance, previous studies failed to find participants with an optimal RF of 4.5b/m (Steffen et al., 2017) and in a sample of late-adults the mean of RF was between 6 and 6.5 b/m (Hasuo et al., 2018). The *mean* RF can also be calculated in “Individual” studies, so for this parameter we pooled “Optimal RF” and “Individual” studies (*n* = 85, Fig. 6, 4th row). In total, only 20 studies reported the *mean* RF (e.g., Paul & Garg, 2012; Prinsloo et al., 2011).

The optimal RF for each participant/patient and the established Preset-Pace breathing rate for all samples should bemonitored to ensure that all participants breathe at the correct rate. In other words, the interpretation of HRVB findings may not be accurate if the participants’ breathing rates are not properly monitore (for example, using a respirometer). Only 19 out of 88 studies (e.g., Clamor et al., 2016; Sutarto et al., 2012) reported any sort of monitoring of the breathing rate. Among the “Optimal RF” studies, one reported only the first two sessions with an expected result, because participants breathed close to the RF (~ 5.5–6 b/m) (Gross et al., 2016). Another reported that the breathing rate was higher at the beginning but correct at the end of the HRVB intervention (from ~ 8.5 to 6.3 b/m) (Sutarto et al., 2012). Regarding the “Preset-Pace” studies that monitored the RF, most of them reported correct breathing rates (e.g., Chalaye et al., 2009; Reyes del Paso et al., 2006). However, some studies reported breathing rates that differed from the established protocols (e.g., Bartur et al., 2014; Francis et al., 2016), which highlights the need to ensure that the HRVB intervention is adequately implemented.

For all four categories of HRVB protocols, the *inhalation/exhalation ratio* is an important parameter for breathing-based interventions. In general, longer exhalations than inhalations are recommended (e.g., 4:6) to increase RSA (Lehrer, 2013; Strauss-Blasche et al., 2000), even though the effects of the inhalation-to-exhalation (I/E) ratio on HRV remain unclear (Shaffer & Ginsberg, 2017). From the included studies/interventions, only 27 reported the *inhalation/exhalation ratio*, with the exhalation usually being longer (e.g., Allen & Friedman, 2012; Brabant et al., 2017). Interestingly, two of the *included* studies compared different inhalation/exhalation ratios with contradictory results. While Lin et al. (2014) discovered that an equal inhalation/exhalation ratio was the best option for achieving greater HRV (even though with longer exhalations, HRV was also increased compared to spontaneous breathing), van Diest et al. (2014) discovered that longer exhalations increased the high-frequency parameter of HRV and increased perceived relaxation compared to longer inhalations. Therefore, we recommend reporting the *inhalation/exhalation ratio* as well as the instructions provided to participants. In addition, owing to different outcomes about the best inhalation/exhalation ratio, this facet of the breathing intervention deserves further investigation.

Although these breathing parameters must be selected for any HRVB intervention, it is noteworthy that approximately 2/3 of the included studies did not provide complete information about these breathing parameters. Furthermore, this lack of methodological information increases the risk of bias (*see below*) of both experimental and quasi-experimental studies and makes the replication of the interventional studies very difficult (Michie et al., 2009).

Last but not least, we encourage measurement of the breathing rate as well as the inhalation/exhalation ratio to be sure that the prescribed intervention is followed by the participants/patients. Respiratory monitoring is also recommended to detect dysfunctional breathing behaviors like apnea or over breathing during the practice of HRVB (Shaffer & Meehan, 2020). There are several devices that can be used for such a purpose. The respirometer is an optimal solution to assess the breathing rate of the user. It consists of a flexible sensor band that detects the thoracic or abdominal expansion during breathing. As an alternative, HRV software can also extract breathing data; this information is obtained after the practice, however (e.g., Capdevila et al., 2021).

Interbeat interval (IBI) is the basic element of HRVB and it can be measured using different technologies. Briefly, an electrocardiogram (ECG) detects the IBI with high accuracy, but it is more invasive and difficult to use than other methods. On the other hand, a photoplethysmograph (PPG), which optically detects pulse waves by assessing changes in light absorption caused by blood flow, is easier to use, more comfortable for the user and can be used daily (e.g., you can use an app on your own smartphone). However, PPG does not detect the beat-to-beat interval as accurately as the ECG and it is influenced by the breathing pace, the recording place, and basal sympathetic activation (Allen, 2007; Dagher et al., 2020; Lu et al., 2009; Shabaan et al., 2020; Shaffer & Combatalade, 2013; Shaffer & Meehan, 2020). Therefore, PPG is less accurate during a paced breathing protocol with the RF range. Interestingly, Jan et al. (2019) compared ECG and PPG on healthy subjects with/without controlled breathing. They found that ECG signals were more precise than PPG wave signals during respiratory fluctuations like during HRV detection.

#### Controlling Contextual Factors

Despite the fact that contextual factors may also impact the experimental results, they were not often included in the selected studies. These factors, for example, from taking a certain medication to just the need to use the toilet (*bladder distension*) (Heathers, 2014), have the potential to alter HRV parameters (for a review, Laborde et al., 2017). From rows seven to 13 in Fig. 6, we assessed the number of studies that just reported the main contextual factors that could affect breathing and cardiovascular parameters such as HRV indices.

One of the basic factors for cardiovascular assessments is body position (Watanabe et al., 2007). Significant differences in the main parameters of HRV have been found between supine, sitting or standing positions. HRV parameters increased in supine positions and decreased in standing positions (Young & Leicht, 2011). *Body position* during the HRVB sessions, whether in the laboratory or home, was reported in less than half of the studies (e.g., Patron et al., 2013; Wang et al., 2010).

The only contextual variable that was reported by most of the studies was *Eyes* (open or closed). However, this is a masked outcome, because we considered studies that used a display or screen as a biofeedback interface as “eyes open” (e.g., Eddie et al., 2018; Kudo et al., 2014). Although, to the best of our knowledge, there is no evidence reporting that open or closed eyes influence cardiovascular parameters, it could affect the levels of concentration, relaxation, or distraction.

We united all factors related to the physical environment under the same variable, *Room*: light, noise, smell and temperature; and we considered studies reporting at least one of these variables as “reported” for this variable. Even so, only 27% of the studies reported even one *Room* variable (e.g., Chen et al., 2016; Schumann et al., 2019). Physical and environmental conditions could affect HRV parameters (Laborde et al., 2017), so it is relevant not only to control them, but also explain them in the paper. For instance, noise could increase sympathetic activity (Lee et al., 2010) and smell might also affect cardiovascular parameters, as aromatherapy has been observed to increase parasympathetic activity (Chang & Shen, 2011; Huang & Capdevila, 2017).

Circadian rhythms also affect our metabolism, including cardiovascular parameters (Mistry et al., 2017). The time of day when HRVB was applied, and its effects assessed, could thus impact the final outcomes depending on the experimental design. In a within-subject design, each participant should be measured consistently at the same time of day, while in a between-subject design, all participants in the same group should be measured simultaneously. Again, a low percentage of studies reported time of day (21%) (e.g., Deschodt-Arsac et al., 2018; Henriques et al., 2011).

Even lower is the number of studies that reported how many participants/patients were together on the HRVB sessions (12%) (e.g., Climov et al., 2014; Patron et al., 2020). Again, there is no evidence that proves the number of participants in a room affects cardiovascular parameters, but because it is a methodological parameter it should be reported as well. For example, being in a social context could both increase or decrease social discomfort, which would affect the sympathetic/parasympathetic activity.

Finally, not even 1/3 of the studies reported that instructions like ‘do not smoke for 2 hours before’ or ‘sleep at least 6 hours the previous night’ were given to the participants before the HRVB intervention or cardiovascular assessment (e.g., Bartur et al., 2014; Caldwell & Steffen, 2018). What’s more, only 35% of them reported measurement of at least one of these instructions or conditions (e.g., Meule et al., 2012; Tsai et al., 2015). In other words, several studies required some conditions to participate in the HRVB sessions or cardiovascular assessment, but then these conditions were neither measured nor enforced. It is well known that some drugs of abuse (Quintana et al., 2013; Sjoberg & Saint, 2011), antidepressant medications (Kemp et al., 2010), physical activity (Melanson, 2000; Stanley et al., 2013) or sleep quality (Meerlo et al., 2008) could alter the cardiovascular response and autonomic functions (like HRV). Thus, it is crucial to first indicate to the participants that they must follow certain rules, second check them and finally act in case these conditions are not followed.

### Risk of Bias

**Fig. 7:**
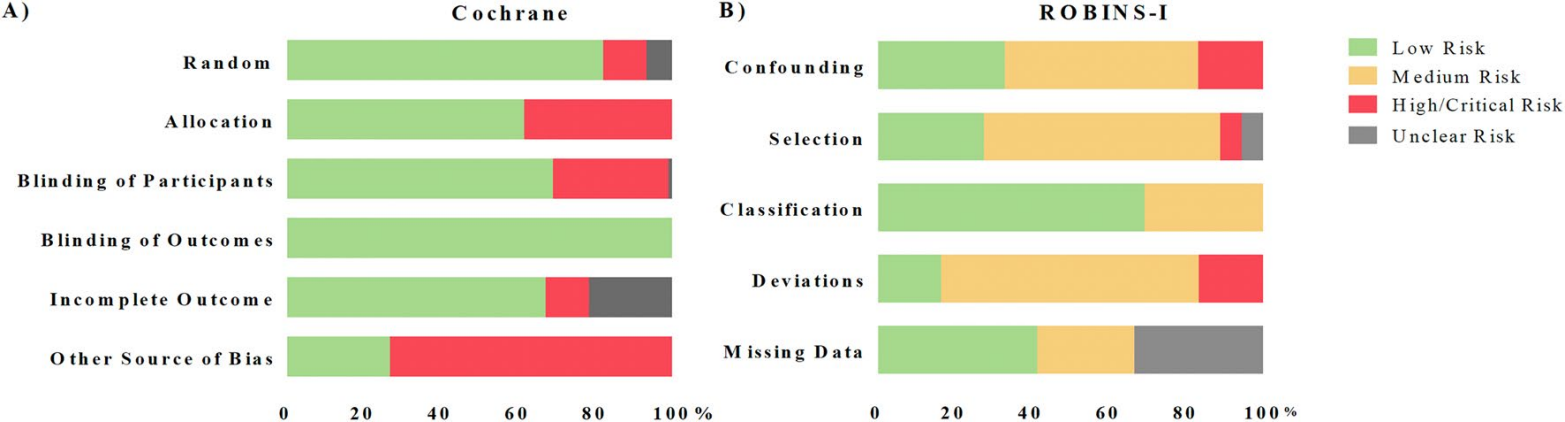
Risk of Bias for the experimental and quasi-experimental studies/interventions. **A** Risk of Bias for Experimental (*n* = 107) studies; **B** Risk of Bias for Quasi-Experimental studies (*n* = 36). Values are expressed in terms of percentage of studies with a certain score. The “Cochrane Risk of Bias Tool for Randomised Trials” tool was used for experimental studies. The 5-Score system of the ROBINS-I was reformulated using the terminology of Cochrane, and reduced to 3 categories, merging Moderate and Serious Risk of Bias into a “Medium Risk” category, in order to simplify its interpretation and assessment. Those items related to “reporting outcomes” were not included, because it was not the objective of this review

In general, experimental studies showed lower levels of risk of bias (*methodological quality*) than quasi-experimental (Fig. 7). Experimental studies showed risk scores in *Other sources of bias*, mainly due to a lack of methodological information and protocol details. This domain was assessed as “low risk” in less than 30% of the studies (Fig. 7A). The other domains were assessed as “low risk” in more than 60% of the studies. Surprisingly, 17% of the studies did not properly address (or report) the randomized designation of participants to the experimental/control groups. As experimental designs are based on randomization, this issue should be clarified in future studies. Finally, due to the character of HRVB interventions, it is not surprising to find that all studies correctly addressed the domain *Blinding of outcome assessment*, which is about the need to blind outcome assessors from knowledge of which intervention a participant received.

Quasi-experimental studies (Fig. 7B), on the other hand, showed medium methodological quality. *Deviation (Bias due to deviations from intended interventions)* was the domain with the highest risk of bias, since it presented lower levels of “low risk” (only 17% of the studies). It refers to the homogeneity of the intervention between participants. It is important tomonitor and/or guarantee that all participants follow the same intervention, because without monitoring it, one cannot ensure that the participants were comparable. Because most of the interventions were home-based in HRVB interventions, some extra tools are needed to guarantee the homogeneity of the intervention, for example the use of an electronic diary to check the HRVB sessions at home using mHealth technology. *Selection (Bias in selection of participants into the study)* and *Confounding (Bias due to confounding)* are related to enrolment of participants, and to the characteristics of participants, respectively. Both criteria could increase the variability of the data and mask the effect of HRVB interventions.If the baseline characteristics of participants and correct enrolment are not adequately controlled, the sample could be biased and could skew the effects attributable to the HRVB interventions. These two domains presented “low risk” levels in only 28–33% of the selected studies. The domain of *Missing data (Bias due to missing data)* was well addressed in 42% of studies. It is also relevant for HRVB interventions, because a high number of missing participants could indicate that the HRVB intervention is not adequate for all participants. Finally, the domain *Classification (Bias in classification of interventions)* showed adequate levels of methodological quality, as 69% of the studies presented “low risk”.

### Methodological Guidelines and Reporting Checklist

The third and most important objective of this systematic review was to provide guidelines to improve both the application of HRVB and the reporting of the methodological information regarding the HRVB protocol, which is crucial to allow replication between research groups and clinical practitioners. Previous publications have already proposed guidelines for HRV (Catai et al., 2020; Laborde et al., 2017; Quintana et al., 2016), but to the best of our knowledge there are not any specific guidelines for HRVB yet. Thereby, we propose a eight-point guideline/checklist to consider before the HRVB intervention and while writing the scientific manuscript. In addition, we present a checklist table to facilitate the implementation of the guidelines (Table 2), because checklists are excellent tools to improve the quality of research reports (Han et al., 2017). We advise future users to adapt the checklist for each experiment or intervention. For example, in a single case study there are no experimental groups, so one just needs to indicate the age of the patient, not the average.

(0) *Pre-Registration or Registration Report*: Preregistration (a short document answering basic questions about the study design and the analysis plan before starting the study) has already become a norm in clinical trials, because it is an efficient way to reduce questionable research practices like *p*-hacking or HARKing and consequently to increase the possibility of replication. Alternatively, a “registration report” answers the basic questions of the study and explains the study design in a more rigorous format and is submitted to a peer-reviewed journal. When the study is completed, the reviewers need only check the registration report and if the researchers followed the original plan, the final paper is published (Nosek et al., 2018; Chamber et al., 2014). We therefore encourage researchers to preregister or publish a registration report before conducting an HRVB study or clinical trial.
(1) *Sample*: Regarding the type of sample, it is relevant to provide information for each experimental group regarding biological sex and age. Optionally, we also recommend indicating the weight and the height if it is possible to obtain this information without disturbing the intervention. One of the threats to reproducible science is low statistical power (small sample size, small effects or both), which is a risk factor for obtaining false-positive and false-negative results as well as an inflated effect size (Button et al., 2013). Thus, we encourage calculation of the statistical power in advance. Another good practice for estimating the magnitude of the intervention is to include the effect size of the intervention in the final report (Schäfer & Schwarz, 2019).
(2) *Allocation*: Directly related to the sample, it is relevant to indicate how participants/patients were recruited and contacted, if they were paid or compensated in any way and if they were aware of all conditions of the experiment (e.g. being part of the control group). As *allocation* was not well covered in the risk of bias, we strongly recommend reviewing this point. Allocation also refers to the inclusion and exclusion criteria, which must be clear in the manuscript. Even though it was not a critical item in the risk of bias, the assignment of each participant to the experimental or control groups is also a relevant issue.
(3) *Missing participants and data*: Working with humans implies more experimental death and loss of participants as the experiment involves more time and effort. High levels of missing participants could indicate that the intervention was too demanding and involved a self-selection bias, as only highly motivated participants finished it. Missing data points could imply that the assessment and/or equipment are not reliable or even valid. Therefore, detailed information about missing participants and missing data, including the reasons, should be provided. We recommend presenting this information in a diagram format.
(4) *Breathing protocol*: This is the core of the intervention and should be explained in detail. We suggest avoiding referring to the breathing protocol of a previous study. Not all studies are accessible for everyone and usually the referred protocol is adapted. The level of “adaptation” is often unknown to the scientific community and a lot of crucial information is lost.
  - *Type*: The type of HRVB intervention needs to be indicated clearly as the terminology HRVB is used for three similar but distinct breathing techniques.
  - *RF detection*: For interventions applying a previous RF detection, information about the ranges, the minutes of each range and the pause between ranges should be indicated. Information about when the RF was assessed is also relevant.
  - *Mean RF*: Except for the “Preset-Pace” or slow-breathing intervention, knowing the mean RF is relevant to connect the sample type with the average RF and, for example, apply a more accurate “Preset-Pace” or fixed breathing ratio in future studies.
  - *Preset RF*: Obviously, the breathing rate used in studies applying a “Preset-Pace” or slow-breathing intervention must be indicated.
  - Monitoring *of the breathing rate*: Once the RF or the preset pace has been established (except for in the “individual” studies), monitoring whether participants/patients breathe at the correct ratio is crucial to assure that the intervention was accomplished correctly. This assessment, however, requires specialized equipment. Respirometers are non-invasive, easy-to-use tools to measure the respiratory rate and the inhalation/exhalation ratio.
  - *Inhalation, holding and exhalation*: As the inhalation/exhalation ratio could affect the cardiovascular system and consequently the efficacy of the HRVB intervention, this information should be reported.
(5) *Breathing Intervention*: All the information regarding laboratory and home practices, number of sessions, minutes per sessions as well as the control mechanisms to guarantee that the intervention was followed correctly are extremely relevant because it is, together with the “breathing protocol,” the core of the HRVB intervention. We strongly advise reporting this information.
(6) *Conditions of the intervention*: The physical and contextual conditions of the breathing intervention during the laboratory or clinical sessions are easy to control. Thus, we encourage researchers to control and report them. The contextual conditions during home practices are more difficult to control, however, as each participant/patient has their own life, for example, different light environments, noise backgrounds, working routines, etc. On the other hand, the current mHealth technology allows us to record practice frequency and duration data from the home breathing sessions, including whether participants/patients are following the prescribed breathing rate, number of sessions and duration.
  - *Laboratory Conditions*: As a minimum, the light, temperature, body position, time of day and the number of participants at the same time ought to be reported as these factors can alter the cardiovascular system. Additionally, we also recommend indicating alterations in smell or ambient aroma, humidity, and eyes (open or closed).
  - *Recommendations for home-practice sessions*: The time of day, the type of room, ambient conditions, and so on, should be noted.
  - *Recommendation for participants*: All the indications that participants received regarding their homeostatic state such as the amount of sleep or caffeine intake must be written as well as the control measures to guarantee the recommendations are fulfilled.
  - *Strategies to control home practices*: Indicate the strategies and tools to ensure that the prescribed breathing regime is followed.
(7) *Equipment*: As there are several technologies and brands available to assess breathing, apply HRVB and measure HRV, precisely indicating the different apparatus used is recommended. Equipment to measure the breathing rate, like respirometers, is highly recommended.

### Strengths and Limitations

The main limitation of the present review is the lack of any efficiency measurement to determine which category and protocol of HRVB is the most recommended. However, the comparison between the different categories of HRVB interventions in terms of efficacy was not within the scope of this review. Hence, we suggest checking other published reviews and meta-analyses that assessed the efficacy of HRVB (Gevirtz, 2013; Goessl et al., 2017; Herbell & Zauszniewski, 2019; Jimenez Morgan & Molina Mora, 2017; Lehrer et al., 2020; Leyro et al., 2019; Pinter et al., 2019; Reneau, 2020). Another limitation of this review is that we did not delve into the RF measurements and HRV analysis. Accurate detection of RR intervals can be impaired by such factors as presence of ectopic beats or other abnormal heart rhythms, line, movement, or EMG artifacts, etc. Thus, we strongly recommend including a plan for artifact prevention and data analysis before starting the HRV study.

On the other hand, this systematic review pioneers the analysis of different HRVB interventions in terms of methodological quality, reporting information and risk of bias. In addition, even though we could not find full-text versions of eight studies and we only included studies in English, Catalan or Spanish, we searched four of the major databases following the PRESS guidelines, thus we expect to have located the main studies applying HRVB interventions.

## Conclusion

There are three main approaches to implement an HRVB intervention, which we categorized as: “Optimal RF”, “Individual” and “Preset-Pace”. All three are based on breathing at the resonance frequency (RF) to enhance the activation of the vagus nerve and parasympathetic system. While the “Optimal RF” and “Individual” categories are actual biofeedback techniques, “Preset-Pace” applies the most common RF breathing rate, 6b/m. Beyond this general categorization, there are almost as many HRVB protocols as studies in terms of total duration of the intervention, minutes of breathing, number of sessions per day and week, laboratory sessions combined with home sessions and so on. This variety of methodological protocols, even though they are based on the protocols proposed by Lehrer and colleagues (Lehrer et al., 2000, 2013), hinders comparison between studies. Thereby, future systematic reviews and meta-analyses ought to analyze the efficiency of the different methodological approaches to HRVB.

This methodological variety, however, was not well reported in most of the studies and we detected several relevant deficiencies in the published studies that applied an HVRB intervention. This systematic review does not intend to criticize these studies, but rather to offer an opportunity to improve future research. Therefore, we propose methodological guidelines for designing the HRVB intervention and writing the manuscript to firstly rethink the most relevant factors and contextual variables related to HRVB and secondly report all relevant methodological details. Finally, we also propose a checklist for better implementation of the guidelines.

## Supplementary Information

Below is the link to the electronic supplementary material.

Supplementary material 1 (DOCX 146.9 kb)

## References

[CR1] Allen B, Friedman BH (2012). Positive emotion reduces dyspnea during slow paced breathing. Psychophysiology.

[CR2] Allen J (2007). Photoplethysmography and its application in clinical physiological measurement. Physiological Measurement.

[CR3] Alvares GA, Quintana DS, Hickie IB, Guastella AJ (2016). Autonomic nervous system dysfunction in psychiatric disorders and the impact of psychotropic medications: A systematic review and meta-analysis. Journal Of Psychiatry And Neuroscience.

[CR4] Appelhans BM, Luecken LJ (2006). Heart Rate Variability as an index of regulated emotional responding. Review of General Psychology.

[CR5] Baker M, Peny D (2016). Is there a reproducibility crisis?. Nature.

[CR6] Bartur G, Vatine JJ, Raphaely-Beer N, Peleg S, Katz-Leurer M (2014). Heart rate autonomic regulation system at rest and during paced breathing among patients with CRPS as compared to age-matched healthy controls. Pain Medicine (Malden, Mass.).

[CR7] Becker JB, Prendergast BJ, Liang JW (2016). Female rats are not more variable than male rats: A meta-analysis of neuroscience studies. Biol Sex Differ.

[CR8] Beery AK, Zucker I (2011). Sex bias in neuroscience and biomedical research. Neuroscience And Biobehavioral Reviews.

[CR9] Begley CG, Ellis LM (2012). Drug development: Raise standards for preclinical cancer research. Nature.

[CR10] Brabant O, van de Ree M, Erkkilä J (2017). The effect of resonance frequency breathing when used as a preparatory exercise in music psychotherapy: A single-case experimental study of a client with anxiety disorder. The Arts in Psychotherapy.

[CR11] Brinkmann AE, Press SA, Helmert E, Hautzinger M, Khazan I, Vagedes J (2020). Comparing effectiveness of HRV-biofeedback and mindfulness for workplace stress reduction: A randomized controlled trial. Applied Psychophysiology And Biofeedback.

[CR12] Brown RP, Gerbarg PL (2005). Sudarshan Kriya Yogic breathing in the treatment of stress, anxiety, and depression. Part II–clinical applications and guidelines. Journal Of Alternative And Complementary Medicine.

[CR13] Brown RP, Gerbarg PL (2005). Sudarshan Kriya yogic breathing in the treatment of stress, anxiety, and depression: part I-neurophysiologic model. Journal Of Alternative And Complementary Medicine.

[CR14] Button KS, Ioannidis JPA, Mokrysz C, Nosek BA, Flint J, Robinson ESJ (2013). Power failure: Why small sample size undermines the reliability of neuroscience. Nature Reviews Neuroscience.

[CR15] Caldwell YT, Steffen PR (2018). Adding HRV biofeedback to psychotherapy increases heart rate variability and improves the treatment of major depressive disorder. International Journal Of Psychophysiology.

[CR16] Capdevila L, Parrado E, Ramos-Castro J, Zapata-Lamana R, Lalanza JF (2021). Resonance frequency is not always stable over time and could be related to the inter-beat interval. Scientific Reports.

[CR17] Catai AM, Pastre CM, Godoy MF, Silva ED, Takahashi ACM, Vanderlei LCM (2020). Heart rate variability: Are you using it properly? Standardisation checklist of procedures. Brazilian Journal Of Physical Therapy.

[CR18] Chalaye P, Goffaux P, Lafrenaye S, Marchand S (2009). Respiratory effects on experimental heat pain and cardiac activity. Pain Medicine (Malden, Mass.).

[CR19] Chalmers JA, Quintana DS, Abbott MJ, Kemp AH (2014). Anxiety disorders are associated with reduced heart rate variability: A meta-analysis. Frontiers In Psychiatry.

[CR20] Chambers CD, Feredoes E, Muthukumaraswamy SD, Etchells PJ (2014). Instead of “playing the game” it is time to change the rules: registered reports at AIMS Neuroscience and beyond. AIMS Neurosci.

[CR21] Chang KM, Shen CW (2011). Aromatherapy benefits autonomic nervous systemregulation for elementary school faculty in taiwan. Evidence-Based Complementary and Alternative Medicine.

[CR22] Chen S, Sun P, Wang S, Lin G, Wang T (2016). Effects of heart rate variability biofeedback on cardiovascular responses and autonomic sympathovagal modulation following stressor tasks in prehypertensives. Journal Of Human Hypertension.

[CR23] Clamor A, Koenig J, Thayer JF, Lincoln TM (2016). A randomized-controlled trial of heart rate variability biofeedback for psychotic symptoms. Behaviour Research And Therapy.

[CR24] Climov D, Lysy C, Berteau S, Dutrannois J, Dereppe H, Brohet C, Melin J (2014). Biofeedback on heart rate variability in cardiac rehabilitation: practical feasibility and psycho-physiological effects. Acta Cardiologica.

[CR25] Cohen J (1960). A coefficient of agreement for nominal scales. Educational and Psychological Measurement.

[CR26] Cullins SW, Gevirtz RN, Poeltler DM, Cousins LM, Harpin E, Muench F (2013). An exploratory analysis of the utility of adding cardiorespiratory biofeedback in the standard care of pregnancy-induced hypertension. Applied Psychophysiology And Biofeedback.

[CR27] Dagher L, Hanyuan S, Zhao Y, Marrouche NF (2020). Wearables in cardiology: Here to stay. Heart Rhythm : The Official Journal Of The Heart Rhythm Society.

[CR28] Del Pozo JM, Gevirtz RN, Scher B, Guarneri E (2004). Biofeedback treatment increases heart rate variability in patients with known coronary artery disease. American Heart Journal.

[CR111] del Reyes GA, Cea JI, Gonzalez-Pinto A, Cabo OM, Caso R, Brazal J, Gonzalez MI (2006). Short-term effects of a brief respiratory training on baroreceptor cardiac reflex function in normotensive and mild hypertensive subjects. Applied Psychophysiology And Biofeedback.

[CR29] Deschodt-Arsac V, Blons E, Gilfriche P, Spiluttini B, Arsac LM (2020). Entropy in heart rate dynamics reflects how HRV-biofeedback training improves neurovisceral complexity during stress-cognition interactions. Entropy (Basel).

[CR30] Deschodt-Arsac V, Lalanne R, Spiluttini B, Bertin C, Arsac LM (2018). Effects of heart rate variability biofeedback training in athletes exposed to stress of university examinations. PLoS One.

[CR31] Eddie D, Conway FN, Alayan N, Buckman J, Bates ME (2018). Assessing heart rate variability biofeedback as an adjunct to college recovery housing programs. Journal Of Substance Abuse Treatment.

[CR32] Fanelli D (2018). Opinion: Is science really facing a reproducibility crisis, and do we need it to?. Proceedings of the National Academy of Sciences of the United States of America.

[CR33] Fisher LR, Lehrer PM (2022). A method for more accurate determination of resonance frequency of the cardiovascular system, and evaluation of a program to perform it. Applied Psychophysiology And Biofeedback.

[CR34] Francis HM, Fisher A, Rushby JA, McDonald S (2016). Reduced heart rate variability in chronic severe traumatic brain injury: Association with impaired emotional and social functioning, and potential for treatment using biofeedback. Neuropsychol Rehabil.

[CR35] Geller SE, Koch AR, Roesch P, Filut A, Hallgren E, Carnes M (2018). The more things change, the more they stay the same: A study to evaluate compliance with inclusion and assessment of women and minorities in randomized controlled trials. Academic Medicine.

[CR36] Gevirtz R (2013). The promise of heart rate variability biofeedback: Evidence-based applications. Biofeedback.

[CR37] Glasziou P, Altman DG, Bossuyt P, Boutron I, Clarke M, Julious S, Wager E (2014). Reducing waste from incomplete or unusable reports of biomedical research. The Lancet.

[CR38] Goessl VC, Curtiss JE, Hofmann SG (2017). The effect of heart rate variability biofeedback training on stress and anxiety: A meta-analysis. Psychological Medicine.

[CR39] Gross MJ, Shearer DA, Bringer JD, Hall R, Cook CJ, Kilduff LP (2016). Abbreviated resonant frequency training to augment heart rate variability and enhance on-demand emotional regulation in elite sport support staff. Applied Psychophysiology And Biofeedback.

[CR40] Hallman DM, Olsson EM, von Scheele B, Melin L, Lyskov E (2011). Effects of heart rate variability biofeedback in subjects with stress-related chronic neck pain: A pilot study. Applied Psychophysiology And Biofeedback.

[CR41] Han S, Olonisakin TF, Pribis JP, Zupetic J, Yoon JH, Holleran KM, Lee JS (2017). A checklist is associated with increased quality of reporting preclinical biomedical research: A systematic review. PLoS One.

[CR42] Hanel PH, Vione KC (2016). Do student samples provide an accurate estimate of the general public?. PLoS One.

[CR43] Hassett AL, Radvanski DC, Vaschillo EG, Vaschillo B, Sigal LH, Karavidas MK, Lehrer PM (2007). A pilot study of the efficacy of heart rate variability (HRV) biofeedback in patients with fibromyalgia. Applied Psychophysiology And Biofeedback.

[CR44] Hasuo H, Kanbara K, Sakuma H, Fukunaga M (2018). Awareness of comfort immediately after a relaxation therapy session affects future quality of life and autonomic function: A prospective cohort study on the expectations of therapy. Biopsychosocial Medicine.

[CR45] Hasuo H, Kanbara K, Sakuma H, Yoshida K, Uchitani K, Fukunaga M (2019). Self-Care system for family caregivers of cancer patients using resonant breathing with a portable home device: A randomized open-label study. Journal Of Palliative Medicine.

[CR46] Heathers JA (2014). Everything Hertz: Methodological issues in short-term frequency-domain HRV. Frontiers In Physiology.

[CR47] Henriques G, Keffer S, Abrahamson C, Horst SJ (2011). Exploring the effectiveness of a computer-based heart rate variability biofeedback program in reducing anxiety in college students. Applied Psychophysiology And Biofeedback.

[CR48] Herbell K, Zauszniewski JA (2019). Reducing psychological stress in peripartum women with heart rate variability biofeedback: A systematic review. J Holist Nurs.

[CR49] Higgins JP, Altman DG, Gøtzsche PC, Jüni P, Moher D, Oxman AD, Sterne JA (2011). The Cochrane collaboration’s tool for assessing risk of bias in randomised trials. Bmj.

[CR50] Hildebrandt LK, McCall C, Engen HG, Singer T (2016). Cognitive flexibility, heart rate variability, and resilience predict fine-grained regulation of arousal during prolonged threat. Psychophysiology.

[CR51] Huang L, Capdevila L (2017). Aromatherapy improves work performance through balancing the autonomic nervous system. Journal Of Alternative And Complementary Medicine.

[CR52] Ioannidis JP, Allison DB, Ball CA, Coulibaly I, Cui X, Culhane AC, van Noort V (2009). Repeatability of published microarray gene expression analyses. Nature Genetics.

[CR53] Ioannidis JPA, Greenland S, Hlatky MA, Khoury MJ, Macleod MR, Moher D, Tibshirani R (2014). Increasing value and reducing waste in research design, conduct, and analysis. The Lancet.

[CR54] Jan HY, Chen MF, Fu TC, Lin WC, Tsai CL, Lin KP (2019). Evaluation of coherence between ECG and PPG derived parameters on heart rate variability and respiration in healthy volunteers With/Without controlled breathing. Journal of Medical and Biological & Engineering.

[CR55] Jester DJ, Rozek EK, McKelley RA (2019). Heart rate variability biofeedback: implications for cognitive and psychiatric effects in older adults. Aging & Mental Health.

[CR56] Jimenez Morgan S, Molina Mora JA (2017). Effect of heart rate variability biofeedback on sport performance, a systematic review. Applied Psychophysiology And Biofeedback.

[CR57] Kemp AH, Quintana DS, Gray MA, Felmingham KL, Brown K, Gatt JM (2010). Impact of depression and antidepressant treatment on heart rate variability: a review and meta-analysis. Biological Psychiatry.

[CR58] Kennedy JJ, Pretorius M (2008). Integrating a portable biofeedback device into call centre environments to reduce employee stress: results from two pilot studies. Journal of Workplace Behavioral Health.

[CR59] Kudo N, Shinohara H, Kodama H (2014). Heart rate variability biofeedback intervention for reduction of psychological stress during the early postpartum period. Applied Psychophysiology And Biofeedback.

[CR60] Laborde S, Mosley E, Thayer JF (2017). Heart rate variability and cardiac vagal tone in psychophysiological research - recommendations for experiment planning, data analysis, and data reporting. Frontiers In Psychology.

[CR61] Lalanza, J. F., Zapata, R., Losilla, J. M., & Capdevila, L. (2018). A systematic review on the methodology of heart rate variability biofeedback in clinical and experimental interventions. PROSPERO, CRD42018086748. Available from: http://www.crd.york.ac.uk/PROSPERO/display_record.php?ID=CRD42018086748

[CR63] Lee GS, Chen ML, Wang GY (2010). Evoked response of heart rate variability using short-duration white noise. Autonomic Neuroscience : Basic & Clinical.

[CR64] Lee J, Kim JK, Wachholtz A (2015). The benefit of heart rate variability biofeedback and relaxation training in reducing trait anxiety. Hanguk Simni Hakhoe Chi Kongang.

[CR65] Lehrer P (2013). How does heart rate variability biofeedback work? Resonance, the baroreflex, and other mechanisms. Biofeedback.

[CR66] Lehrer P, Kaur K, Sharma A, Shah K, Huseby R, Bhavsar J, Zhang Y (2020). Heart rate variability biofeedback improves emotional and physical health and performance: A systematic review and meta analysis. Applied Psychophysiology And Biofeedback.

[CR67] Lehrer P, Vaschillo B, Zucker T, Graves J, Katsamanis M, Aviles M, Wamboldt F (2013). Protocol for heart rate variability biofeedback training. Biofeedback.

[CR68] Lehrer P, Vaschillo E (2008). The future of heart rate variability biofeedback. Biofeedback.

[CR69] Lehrer PM (2018). Heart rate variability biofeedback and other psychophysiological procedures as important elements in psychotherapy. International Journal Of Psychophysiology.

[CR70] Lehrer PM, Gevirtz R (2014). Heart rate variability biofeedback: How and why does it work?. Frontiers In Psychology.

[CR71] Lehrer PM, Irvin CG, Lu SE, Scardella A, Roehmheld-Hamm B, Aviles-Velez M, Wamboldt FS (2018). Heart rate variability biofeedback does not substitute for asthma steroid controller medication. Applied Psychophysiology And Biofeedback.

[CR72] Lehrer PM, Vaschillo E, Vaschillo B (2000). Resonant frequency biofeedback training to increase cardiac variability: Rationale and manual for training. Applied Psychophysiology And Biofeedback.

[CR73] Lehrer PM, Vaschillo E, Vaschillo B, Lu SE, Eckberg DL, Edelberg R, Hamer RM (2003). Heart rate variability biofeedback increases baroreflex gain and peak expiratory flow. Psychosomatic Medicine.

[CR74] Leyro TM, Buckman JF, Bates ME (2019). Theoretical implications and clinical support for heart rate variability biofeedback for substance use disorders. Curr Opin Psychol.

[CR62] Li KHC, White FA, Tipoe T, Liu T, Wong MC, Jesuthasan A (2019). The current state of mobile phone apps for monitoring heart rate, heart rate variability, and atrial fibrillation: Narrative review. JMIR mHealth and uHealth.

[CR75] Li X, Zhang T, Song LP, Zhang GG, Xing CX, Chen H (2015). Effects of heart rate variability biofeedback therapy on patients with poststroke depression: a case study. Chinese Medical Journal (Engl).

[CR76] Lin G, Xiang Q, Fu X, Wang S, Wang S, Chen S, Wang T (2012). Heart rate variability biofeedback decreases blood pressure in prehypertensive subjects by improving autonomic function and baroreflex. Journal Of Alternative And Complementary Medicine.

[CR77] Lin IM, Tai LY, Fan SY (2014). Breathing at a rate of 5.5 breaths per minute with equal inhalation-to-exhalation ratio increases heart rate variability. International Journal Of Psychophysiology.

[CR78] Lu G, Yang F, Taylor JA, Stein JF (2009). A comparison of photoplethysmography and ECG recording to analyse heart rate variability in healthy subjects. Journal Of Medical Engineering & Technology.

[CR79] MacKinnon S, Gevirtz R, McCraty R, Brown M (2013). Utilizing heartbeat evoked potentials to identify cardiac regulation of vagal afferents during emotion and resonant breathing. Applied Psychophysiology And Biofeedback.

[CR80] Mamlouk GM, Dorris DM, Barrett LR, Meitzen J (2020). Sex bias and omission in neuroscience research is influenced by research model and journal, but not reported NIH funding. Frontier in Neuroendocrinology.

[CR81] Mather M, Thayer J (2018). How heart rate variability affects emotion regulation brain networks. Current Opinion in Behavioal Sciences.

[CR82] McGowan J, Sampson M, Salzwedel DM, Cogo E, Foerster V, Lefebvre C (2016). PRESS peer review of electronic search strategies: 2015 Guideline Statement. Journal Of Clinical Epidemiology.

[CR83] Meerlo P, Sgoifo A, Suchecki D (2008). Restricted and disrupted sleep: Effects on autonomic function, neuroendocrine stress systems and stress responsivity. Sleep Medicine Reviews.

[CR84] Melanson EL (2000). Resting heart rate variability in men varying in habitual physical activity. Medicine And Science In Sports And Exercise.

[CR85] Meule A, Freund R, Skirde AK, Vogele C, Kubler A (2012). Heart rate variability biofeedback reduces food cravings in high food cravers. Applied Psychophysiology And Biofeedback.

[CR86] Meyer PW, Friederich HC, Zastrow A (2018). Breathe to ease - respiratory biofeedback to improve heart rate variability and coping with stress in obese patients: A pilot study. Mental Health & Prevention.

[CR87] Michie S, Fixsen D, Grimshaw JM, Eccles MP (2009). Specifying and reporting complex behaviour change interventions: The need for a scientific method. Implementation Science.

[CR88] Mistry P, Duong A, Kirshenbaum L, Martino TA (2017). Cardiac clocks and preclinical translation. Heart Failure Clinics.

[CR89] Moher D, Liberati A, Tetzlaff J, Altman DG (2009). Preferred reporting items for systematic reviews and meta-analyses: The PRISMA statement. Plos Medicine.

[CR90] Munafo M, Patron E, Palomba D (2016). Improving managers’ psychophysical well-being: Effectiveness of respiratory sinus Arrhythmia Biofeedback. Applied Psychophysiology And Biofeedback.

[CR91] Munafo MR, Nosek BA, Bishop DVM, Button KS, Chambers CD, du Sert NP, Ioannidis JPA (2017). A manifesto for reproducible science. Nature Human Behaviour.

[CR92] Narita Y, Shinohara H, Kodama H (2018). Resting heart rate variability and the effects of biofeedback intervention in women with low-risk pregnancy and prenatal childbirth fear. Applied Psychophysiology And Biofeedback.

[CR93] NIH (2017). Principles and Guidelines for Reporting Preclinical Research. Retrieved from https://www.nih.gov/research-training/rigor-reproducibility/principles-guidelines-reporting-preclinical-research

[CR94] Noble DJ, Hochman S (2019). Hypothesis: Pulmonary afferent activity patterns during slow, deep breathing contribute to the neural induction of physiological relaxation. Frontiers in Physiology.

[CR95] Nolan RP, Kamath MV, Floras JS, Stanley J, Pang C, Picton P, Young QR (2005). Heart rate variability biofeedback as a behavioral neurocardiac intervention to enhance vagal heart rate control. American Heart Journal.

[CR96] Nosek BA, Ebersole CR, DeHaven AC, Mellor DT (2018). The preregistration revolution. Pnas.

[CR97] Open Science C (2015). Psychology. Estimating the reproducibility of psychological science. Science.

[CR98] Ozier D, Linden W (2018). Heart variability biofeedback as supplementary care for Brain Cancer: A feasibility study. Journal Of Alternative And Complementary Medicine.

[CR99] Patil P, Peng RD, Leek JT (2016). What should researchers expect when they replicate studies? A statistical view of replicability in Psychological Science. Perspective on Psycholgical Science.

[CR100] Patron E, Messerotti Benvenuti S, Favretto G, Valfrè C, Bonfà C, Gasparotto R, Palomba D (2013). Biofeedback assisted control of respiratory sinus arrhythmia as a biobehavioral intervention for depressive symptoms in patients after cardiac surgery: A preliminary study. Applied Psychophysiology And Biofeedback.

[CR101] Patron E, Munafo M, Messerotti Benvenuti S, Stegagno L, Palomba D (2020). Not all Competitions come to harm! Competitive biofeedback to increase respiratory sinus arrhythmia in managers. Front Neurosci.

[CR102] Paul M, Garg K (2012). The effect of heart rate variability biofeedback on performance psychology of basketball players. Applied Psychophysiology And Biofeedback.

[CR103] Perez-Gaido M, Lalanza JF, Parrado E, Capdevila L (2021). Can HRV Biofeedback improve short-term effort recovery? Implications for intermittent load Sports. Applied Psychophysiology And Biofeedback.

[CR104] Pinter A, Szatmari S, Horvath T, Penzlin AI, Barlinn K, Siepmann M, Siepmann T (2019). Cardiac dysautonomia in depression - heart rate variability biofeedback as a potential add-on therapy. Neuropsychiatric Disease And Treatment.

[CR105] Plint AC, Moher D, Morrison A, Schulz K, Altman DG, Hill C, Gaboury I (2006). Does the CONSORT checklist improve the quality of reports of randomised controlled trials? A systematic review. Medical Journal Of Australia.

[CR106] Prinsloo GE, Rauch HGL, Lambert MI, Muench F, Noakes TD, Derman WE (2011). The effect of short duration heart rate variability (HRV) biofeedback on cognitive performance during laboratory induced cognitive stress. Applied Cognitive Psychology.

[CR107] Quintana DS, Alvares GA, Heathers JA (2016). Guidelines for reporting articles on Psychiatry and Heart rate variability (GRAPH): Recommendations to advance research communication. Transl Psychiatry.

[CR108] Quintana DS, Guastella AJ, McGregor IS, Hickie IB, Kemp AH (2013). Heart rate variability predicts alcohol craving in alcohol dependent outpatients: Further evidence for HRV as a psychophysiological marker of self-regulation. Drug And Alcohol Dependence.

[CR109] Reiner R (2008). Integrating a portable biofeedback device into clinical practice for patients with anxiety disorders: Results of a pilot study. Applied Psychophysiology And Biofeedback.

[CR110] Reneau M (2020). Feasibility and acceptability of Heart Rate Variability Biofeedback in a Group of Veterans with Fibromyalgia. Journal Of Alternative And Complementary Medicine.

[CR112] Rusciano A, Corradini G, Stoianov I (2017). Neuroplus biofeedback improves attention, resilience, and injury prevention in elite soccer players. Psychophysiology.

[CR113] Russell ME, Scott AB, Boggero IA, Carlson CR (2017). Inclusion of a rest period in diaphragmatic breathing increases high frequency heart rate variability: Implications for behavioral therapy. Psychophysiology.

[CR114] Russo MA, Santarelli DM, O’Rourke D (2017). The physiological effects of slow breathing in the healthy human. Breathe (Sheff).

[CR115] Sakaki M, Yoo HJ, Nga L, Lee TH, Thayer JF, Mather M (2016). Heart rate variability is associated with amygdala functional connectivity with MPFC across younger and older adults. Neuroimage.

[CR116] Schäfer T, Schwarz MA (2019). The meaningfulness of effect sizes in psychological research differences between sub-disciplines and the impact of potential biases. Frontiers in Psychology.

[CR117] Schmidt JE, Joyner MJ, Tonyan HM, Reid KI, Hooten WM (2012). Psychological and physiological correlates of a brief intervention to Enhance Self-Regulation in patients with Fibromyalgia. Journal of Musculoskeletal Pain.

[CR118] Schmidt S (2009). Shall we really do it again? The powerful concept of replication is neglected in the social sciences. Review of General Psychology.

[CR119] Schuman DL, Killian MO (2019). Pilot study of a single session heart rate variability biofeedback intervention on veterans’ posttraumatic stress symptoms. Applied Psychophysiology And Biofeedback.

[CR120] Schumann A, Kohler S, Brotte L, Bar KJ (2019). Effect of an eight-week smartphone-guided HRV-biofeedback intervention on autonomic function and impulsivity in healthy controls. Physiological Measurement.

[CR121] Shabaan M, Arshid K, Yaqub M, Jinchao F, Zia S (2020). Survey: Smartphone-based assessment of cardiovascular diseases using ECG and PPG analysis. BMC Medical Informatics and Decision Making.

[CR122] Shaffer F, Combatalade DC (2013). Don’t add or miss a beat: A guide to cleaner heart rate variability recordings. Biofeedback.

[CR123] Shaffer F, Ginsberg JP (2017). An overview of heart rate variability metrics and norms. Frontiers in Public Health.

[CR124] Shaffer F, Meehan Z (2020). A practical guide to resonance frequency assessment for heart rate variability biofeedback. Frontier in Neuroscience.

[CR125] Shaw L, Zaichkowsky L, Wilson V (2012). Setting the balance: Using biofeedback and neurofeedback with gymnasts. Journal of Clinical Sport Psychology.

[CR126] Sierra Murguía MA, Rico P, Fraga Sastrías JM (2017). Uso de biofeedback de variabilidad de la frecuencia cardiaca durante la radioterapia como método de distracción cognitiva y autorregulación en un paciente pediátrico: Informe de caso. Psicooncología.

[CR127] Sjoberg N, Saint DA (2011). A single 4 mg dose of nicotine decreases heart rate variability in healthy nonsmokers: Implications for smoking cessation programs. Nicotine & Tobacco Research.

[CR128] Sowder E, Gevirtz R, Shapiro W, Ebert C (2010). Restoration of vagal tone: A possible mechanism for functional abdominal pain. Applied Psychophysiology And Biofeedback.

[CR129] Stanley J, Peake JM, Buchheit M (2013). Cardiac parasympathetic reactivation following exercise: Implications for training prescription. Sports Medicine (Auckland, N. Z.).

[CR130] Steffen PR, Austin T, DeBarros A, Brown T (2017). The impact of resonance frequency breathing on measures of heart rate variability, blood pressure, and mood. Front Public Health.

[CR131] Stern MJ, Guiles RA, Gevirtz R (2014). HRV biofeedback for pediatric irritable bowel syndrome and functional abdominal pain: A clinical replication series. Applied Psychophysiology And Biofeedback.

[CR132] Sterne JA, Hernán MA, Reeves BC, Savović J, Berkman ND, Viswanathan M, Higgins JP (2016). ROBINS-I:A tool for assessing risk of bias in non-randomised studies of interventions. Bmj.

[CR133] Strauss-Blasche G, Moser M, Voica M, McLeod DR, Klammer N, Marktl W (2000). Relative timing of inspiration and expiration affects respiratory sinus arrhythmia. Clinical And Experimental Pharmacology And Physiology.

[CR134] Stromberg SE, Russell ME, Carlson CR (2015). Diaphragmatic breathing and its effectiveness for the management of motion sickness. Aerospace Medicine and Human Performance.

[CR135] Sutarto AP, Wahab MN, Zin NM (2012). Resonant breathing biofeedback training for stress reduction among manufacturing operators. International Journal Of Occupational Safety And Ergonomics : Jose.

[CR136] Sutarto AP, Wahab MN, Zin NM (2013). Effect of biofeedback training on operator’s cognitive performance. Work (Reading, Mass.).

[CR137] Tan G, Dao TK, Farmer L, Sutherland RJ, Gevirtz R (2011). Heart rate variability (HRV) and posttraumatic stress disorder (PTSD): A pilot study. Applied Psychophysiology And Biofeedback.

[CR138] Tavares BS, de Paula Vidigal G, Garner DM, Raimundo RD, de Abreu LC, Valenti VE (2017). Effects of guided breath exercise on complex behaviour of heart rate dynamics. Clinical Physiology And Functional Imaging.

[CR139] Thayer JF, Ahs F, Fredrikson M, Sollers JJ, Wager TD (2012). A meta-analysis of heart rate variability and neuroimaging studies: Implications for heart rate variability as a marker of stress and health. Neuroscience And Biobehavioral Reviews.

[CR140] Tsai HJ, Kuo TB, Lee GS, Yang CC (2015). Efficacy of paced breathing for insomnia: Anhances vagal activity and improves sleep quality. Psychophysiology.

[CR141] Vagedes J, Fazeli A, Boening A, Helmert E, Berger B, Martin D (2019). Efficacy of rhythmical massage in comparison to heart rate variability biofeedback in patients with dysmenorrhea-A randomized, controlled trial. Complementary Therapies In Medicine.

[CR142] van der Zwan JE, de Vente W, Huizink AC, Bogels SM, de Bruin EI (2015). Physical activity, mindfulness meditation, or heart rate variability biofeedback for stress reduction: a randomized controlled trial. Applied Psychophysiology And Biofeedback.

[CR143] Van Diest I, Verstappen K, Aubert AE, Widjaja D, Vansteenwegen D, Vlemincx E (2014). Inhalation/Exhalation ratio modulates the effect of slow breathing on heart rate variability and relaxation. Applied Psychophysiology And Biofeedback.

[CR144] Voss A, Heitmann A, Schroeder R, Peters A, Perz S (2012). Short-term heart rate variability–age dependence in healthy subjects. Physiological Measurement.

[CR145] Walker FR, Pfingst K, Carnevali L, Sgoifo A, Nalivaiko E (2017). In the search for integrative biomarker of resilience to psychological stress. Neuroscience And Biobehavioral Reviews.

[CR146] Wang SZ, Li S, Xu XY, Lin GP, Shao L, Zhao Y, Wang TH (2010). Effect of slow abdominal breathing combined with biofeedback on blood pressure and heart rate variability in prehypertension. Journal Of Alternative And Complementary Medicine.

[CR147] Watanabe N, Reece J, Polus BI (2007). Effects of body position on autonomic regulation of cardiovascular function in young, healthy adults. Chiropr Osteopat.

[CR148] Weeks DL, Whitney AA, Tindall AG, Carter GT (2015). Pilot randomized trial comparing intersession scheduling of biofeedback results to individuals with chronic pain: Influence on psychologic function and pain intensity. American Journal Of Physical Medicine And Rehabilitation.

[CR149] Wells R, Outhred T, Heathers JA, Quintana DS, Kemp AH (2012). Matter over mind: a randomised-controlled trial of single-session biofeedback training on performance anxiety and heart rate variability in musicians. PLoS One.

[CR150] Windthorst P, Mazurak N, Kuske M, Hipp A, Giel KE, Enck P, Teufel M (2017). Heart rate variability biofeedback therapy and graded exercise training in management of chronic fatigue syndrome: An exploratory pilot study. Journal Of Psychosomatic Research.

[CR151] Woitowich NC, Beery A, Woodruff T (2020). A 10-year follow-up study of sex inclusion in the biological sciences. Elife.

[CR152] Wu W, Gil Y, Lee J (2012). Combination of wearable multi-biosensor platform and resonance frequency training for stress management of the unemployed population. Sensors (Basel).

[CR153] Yasuma F, Hayano J (2004). Respiratory sinus arrhythmia: Why does the heartbeat synchronize with respiratory rhythm?. Chest.

[CR154] Young FL, Leicht AS (2011). Short-term stability of resting heart rate variability: Influence of position and gender. Applied Physiology, Nutrition And Metabolism.

[CR155] Young HA, Benton D (2018). Heart-rate variability: A biomarker to study the influence of nutrition on physiological and psychological health. Behavioural Pharmacology.

[CR156] Yu LC, Lin IM, Fan SY, Chien CL, Lin TH (2018). One-Year Cardiovascular prognosis of the randomized, controlled, short-term heart rate variability biofeedback among patients with coronary artery disease. International Journal Of Behavioral Medicine.

[CR157] Yucha CB, Tsai PS, Calderon KS, Tian L (2005). Biofeedback-assisted relaxation training for essential hypertension who is most likely to benefit?. Journal of Cardiovascular Nursing.

[CR158] Zaccaro A, Piarulli A, Laurino M, Garbella E, Menicucci D, Neri B, Gemignani A (2018). How breath-control can change your life: A systematic review on psycho-physiological correlates of slow breathing. Frontiers In Human Neuroscience.

[CR159] Zauszniewski JA, Au TY, Musil CM (2013). Heart rate variability biofeedback in grandmothers raising grandchildren: Effects on stress, emotions, and cognitions. Biofeedback.

[CR160] Zucker TL, Samuelson KW, Muench F, Greenberg MA, Gevirtz RN (2009). The effects of respiratory sinus arrhythmia biofeedback on heart rate variability and posttraumatic stress disorder symptoms: A pilot study. Applied Psychophysiology And Biofeedback.

[CR161] Zunhammer M, Eichhammer P, Busch V (2013). Do cardiorespiratory variables predict the antinociceptive effects of deep and slow breathing?. Pain Medicine.

