# Supplementary material for: Methods for Heart Rate Variability Biofeedback (HRVB): A Systematic Review and Guidelines
Source: Appl Psychophysiol Biofeedback. 2023 Mar 14;48(3):275–97. doi: 10.1007/s10484-023-09582-6 (PMC10412682; doi:10.1007/s10484-023-09582-6)
Supplement: Supplementary file 1 — Supplementary material 1 (DOCX 146.9 kb) [file 10484_2023_9582_MOESM1_ESM.docx]

**Supplementary Table 1. Search strings for PsycInfo (by PsycNET and OVID)**

| # | Search strings |
| --- | --- |
| 1 | “resonance frequency”* and breathing |
| 2 | “resonant frequency” and breathing |
| 3 | “resonance frequency” and biofeedback |
| 4 | “resonant frequency” and biofeedback |
| 5 | “resonance frequency” and training |
| 6 | “resonant frequency” and training |
| 7 | “heart rate variability” and biofeedback |
| 8 | HRV and Biofeedback |
| 9 | “heart rhythm coherence” |
| 10 | HRVB |
| 11 | #1 OR #2 OR #3 OR #4 OR #5 OR #6 OR #7 OR #8 OR #9 OR #10 |
| 12 | FILTER: Methodology (clinical case study, clinical trial, empirical study, experimental replication, follow up study, longitudinal study, prospective study, retrospective study, field study, focus group, nonclinical case study, treatment outcome, twin study) |
| 13 | FILTER: Period (2000-2021) |

- Without “” in Ovid and additional filter: “Human”

**Supplementary Table 2. Search strings for CINAHL (by EBSCOhost)**

| # | Search strings |
| --- | --- |
| 1 | “resonance frequency” and breathing |
| 2 | “resonant frequency” and breathing |
| 3 | “resonance frequency” and biofeedback |
| 4 | “resonant frequency” and biofeedback |
| 5 | “resonance frequency” and training |
| 6 | “resonant frequency” and training |
| 7 | “heart rate variability” and biofeedback |
| 8 | HRV and Biofeedback |
| 9 | “heart rhythm coherence” |
| 10 | HRVB |
| 11 | #1 OR #2 OR #3 OR #4 OR #5 OR #6 OR #7 OR #8 OR #9 OR #10 |
| 12 | FILTER: Search type (boolean/sentence) |
| 13 | FILTER: Specie (Human) |
| 14 | FILTER: Period (2000-2021) |

**Supplementary Table 3. Search strings for PubMed (by Medline)**

| # | Search strings |
| --- | --- |
| 1 | “resonance frequency” and breathing |
| 2 | “resonant frequency” and breathing |
| 3 | “resonance frequency” and biofeedback |
| 4 | “resonant frequency” and biofeedback |
| 5 | “resonance frequency” and training |
| 6 | “resonant frequency” and training |
| 7 | “heart rate variability” and biofeedback |
| 8 | HRV and Biofeedback |
| 9 | “heart rhythm coherence” |
| 10 | HRVB |
| 11 | #1 OR #2 OR #3 OR #4 OR #5 OR #6 OR #7 OR #8 OR #9 OR #10 |
| 12 | FILTER: Articles type (case reports, classical article, clinical study, clinical trial, clinical trial phase I, clinical trial phase II, clinical trial phase III, clinical trial phase IV, comparative study*, controlled clinical trial, journal article, twin study) |
| 13 | FILTER: Specie (Human) |
| 14 | FILTER: Period (2000-2021) |

**Supplementary Table 4. Search strings for Core Collection of Web of Science (by Web of Science)**

| # | Search strings |
| --- | --- |
| 1 | “resonance frequency” and breathing |
| 2 | “resonant frequency” and breathing |
| 3 | “resonance frequency” and biofeedback |
| 4 | “resonant frequency” and biofeedback |
| 5 | “resonance frequency” and training |
| 6 | “resonant frequency” and training |
| 7 | “heart rate variability” and biofeedback |
| 8 | HRV and Biofeedback |
| 9 | “heart rhythm coherence” |
| 10 | HRVB |
| 11 | #1 OR #2 OR #3 OR #4 OR #5 OR #6 OR #7 OR #8 OR #9 OR #10 |
| 12 | FILTER: Type (article, proceedings paper) |
| 13 | FILTER: Category (psychology clinical or physiology or public environmental occupational health or neurosciences or multidisciplinary sciences or psychology or psychiatry or psychology biological or psychology experimental or cardiac cardiovascular systems or clinical neurology or psychology applied or ergonomics or sport sciences or psychology multidisciplinary or engineering biomedical) |
| 14 | FILTER: Period (2000-2021) |

**Supplementary Table 5:** Sample and experimental design of the included studies (n=143, interventions n=145)

| **References** | **Sample**  **Initial-Final** | **Condition** | **Biological sex**  **(male-female)** | **Age (SD)** | **Design**  **Type** | **Control Intervention/Group** |
| --- | --- | --- | --- | --- | --- | --- |
| **Allen and Friedman (2012)(1)** | (80-71) | University Students | (36-35) | 20.08 (0.22)^3^ | Exp/WS | Breathing + Positive Pictures (IAPS) |
| **Amichai et al. (2019)(2)** | (40-40) | Patients (cerebral palsy) | (29-11) | 9 | Q-Exp | Healthy Controls |
| **Amjadian et al. (2020)(3)** | (60-60) | Patients (CABG) | (41-19) | 56 | Exp/BS | Religious-based therapy / TAU |
| **Bartur et al. (2014)(4)** | (20-20) | Patients (CRPS) | (12-8) | 32.75 | Q-Exp | Healthy Controls |
| **Bates et al. (2019)(5)** | (49-41) | Alcohol use and AUD | NI | 21.4 (1.9) | Exp/BS | Vanilla task |
| **Beckham et al. (2013)(6)** | (15-15) | Patients (depression) | (0-15) | 30.8 | Q-Exp | No control Group |
| **Brinkmann et al. (2020)(7)** | (61-52) | Healthy Adults | (15-37) | 43.27 (10.45) | Exp/BS | Mindfulness / Waiting list |
| **Burch et al. (2020)(8)** | (38-34) | Patients (cancer) | (5-29) | 60 | Exp/BS | TAU |
| **Caldwell et al. (2018)(9)** | (32-30) | University Students (depression) | (0-30) | 20.31 | Exp/BS | TAU / Health Controls |
| **Chalaye et al. (2009/1)(10)** | (20-20) | Healthy Adults | (11-9) | 25.1 (5.6) | Exp/WS | Preset-Pace, Natural, Fast (breathing) videogame |
| **Chalaye et al. (2009/2)(10)** | (20-20) | Healthy Adults | (11-9) | 25.1 (5.6) | Exp/WS | HRVB, Natural, Fast (breathing), videogame |
| **Chang et al. (2020)(11)** | (40-35) | Patients (AIS) | (18-17) | 67.4 (9.7) | Exp/BS | CON |
| **Chelidoni et al. (2020)(12)** | (75-75) | Healthy Adults | (27-48) | 32.32 (10.46) | Exp/BS | Mindfulness / CON |
| **Chen et al. (2016/1)(13)** | (32-32) | University Students (prehypertension) | (24-8) | 21.5 (0.18) | Exp/BS | SB, CON |
| **Chen et al. (2016/2)(13)** | (32-32) | University Students (prehypertension) | (24-8) | 21.5 (0.18) | Exp/BS | CON |
| **Cheng et al. (2019)(14)** | (50-50) | University Students | NI | 22 | Exp/BS | Different breathing duration, CON |
| **Clamor et al. (2016)(15)** | (90-84) | University Students (psychotic)^2^ | (26-58) | 26.93 | Exp/BS | Muscle relaxation / Reading |
| **Climov et al (2014)(16)** | (31-24) | Patients (CAD) | (31-0) | 56.5 | Exp/BS | TAU |
| **Cullins et al (2013)(17)** | (47-47) | Pregnant Women (hypertension) | (0-47) | 31.6 | Q-Exp | TAU |
| **De Couck et al. (2019)(s2)(18)** | (56-56) | University Students | (30-26) | 24.2 | Exp/BS | CON |
| **de Zambotti et al. (2019)(19)** | (16-16) | Healthy Adults (insomnia) | (0-16) | 43.44 (13.31) | Exp/WS | CON |
| **del Paso et al. (2006)(20)** | (22-22) | University Staff (hypertension) | (6-16) | 48.3 | Q-Exp | Healthy Controls |
| **del Pozo et al. (2004)(21)** | (69-61) | Patients (CAD) | (42-21) | 67 | Exp/BS | TAU |
| **Deschodt-Arsac et al. (2018)(22)** | (18-18) | University Students | (13-5) | 20.5 (1.5) | Exp/BS | Healthy Controls |
| **Deschodt-Arsac et al. (2020)(23)** | (19-16) | Healthy Adults (with mild stress) | (11-5) | 42.5 (15.1) | Q-Exp | CON |
| **Dessy et al. (2020)(24)** | (26-26) | University Students | (26-0) | 23 | Exp/BS | HRVB + Neurofeedback |
| **Dziembowska et al. (2016)(25)** | (48-41) | Football and Basketball Players | (41-0) | 18.34 (1.36) | Exp/BS | CON |
| **References** | **Sample**  **Initial-Final** | **Condition** | **Biological sex**  **(male-female)** | **Age (SD)** | **Design**  **Type** | **Control Intervention/Group** |
| **Ebben et al. (2009)(26)** | (10-9) | Healthy Adults | (3-7) | 20 | Exp/BS | CON |
| **Eddie et al. (2018)(27)** | (46-38) | University Students (SUD) | (33-13) | 23.6 (5.0) | Q-Exp | Waiting List |
| **Eddie et al. (2014)(28)** | (48-41) | Patients (SUD) | (41-0) | 21.85 | Exp/BS | TAU + 6b/m (5min only) |
| **Francis et al. (2016)(29)** | (60-57) | Patients (TBI) | (46-14) | 46.3 | Q-Exp | Healthy Controls |
| **Francis et al. (2016)(30)** | (60-58) | University Students and Healthy adults | (15-43) | 23.84 (10) | Exp/BS | Breathing without biofeedback information |
| **Giardino et al. (2004)(31)** | (20-20) | Patients (COPD) | (10-10) | 63 (9.6) | Q-Exp | No control Group |
| **Gray et al. (2019)(32)** | (8-8) | Sexual offenders (ID diagnosed) | (8-0) | 31.3 (7.5) | Q-Exp | No control Group |
| **Gross et al. (2016)(33)** | (9-7) | Healthy Adults | (3-6) | 45.88 (14.48) | Q-Exp | No control Group |
| **Gruzelier et al. (2014)(34)** | (64-64) | Dance Students | (22-42) | Not Informed | Exp/BS | Neurofeedback, Choreology, CON |
| **Hallman et al. (2011)(35)** | (24-23) | Patients (neck pain) | (2-21) | 40.5 | Exp/BS | Only 2 HRVB sessions |
| **Hassett et al. (2007)(36)** | (12-12) | Patients (fibromyalgia) | (0-12) | 38.5 (12.5) | Q-Exp | No control Group |
| **Hasuo et al. (2020)(37)** | (50-50) | Patients (cancer) with insomnia | (30-20) | 66.4 (12.5) | Exp/BS | With or without home practice (HRVB) |
| **Hasuo et al. (2019)(38)** | (54-54) | Healthy Adults (caregivers) | (20-34) | 63 (11) | Exp/BS | TAU |
| **Hasuo et al. (2020)(39)** | (69-69) | Healthy Adults (caregivers) | (23-46) | 63.6 (11) | Exp/BS | With or without home practice (HRVB) |
| **Hasuo et al. (2018)(40)** | (44-42) | Healthy Adults (caregivers) | (16-28) | 63.35 | Q-Exp | No control Group |
| **Henriques et al. (2011 /s2)(41)** | (51-35) | University Students | (8-27) | Not Informed | Exp/BS | Delayed time |
| **Hsieh et al. (2020)(42)** | (159-135) | Nurses (with history of being abused) | (16-119) | 35.61 (8.18) | Exp/BS | Two HRVB interventions, CON |
| **Huang et al. (2019)(43)** | (161-150) | Patients (overactive bladder syndrome) | (0-150) | 60.41 (11.4) | Exp/BS | Breathing without biofeedback information |
| **Hunter et al. (2019)(44)** | (140-140) | University Students | (32-108) | 20.28 (2.68) | Exp/BS | Access (or not) to smartphone |
| **Jester et al. (2019)(45)** | (20-18) | Patients (depression) | (6-14) | 78.5 (9.18) | Q-Exp | No control Group |
| **Karavidas et al. (2007)(46)** | (11-8) | Patients (depression) | (4-7) | 45 (10.8) | Q-Exp | No control Group |
| **Kohlenberg et al. (2020)(47)** | (20-20) | Healthy Adults | (8-12) | 26-62 | Exp/BS | HRVB + extra biofeedback (oximeter) |
| **Kennedy and Pretorious (2008)(48)^1^** | (338(23)-338(19)) | Healthy Adults | (8 (4)-12 (15)) | Not Informed | Exp/BS (Q-Exp) | CON (No control group) |
| **Kudo et al. (2014)(49)** | (55-55) | Patients (postpartum stress) | (0-55) | 31.95 | Exp/BS | CON |
| **Laborde et al. (2017)(50)** | (16-14) | Adolescents (developmental disability) | (14-2) | 17.39 | Exp/WS | Audiobook |
| **Laborde et al. (2019)(51)** | (120-107) | University Students | (73-47) | 25 | Exp/BS | Watch TV |
| **Lee and Finkelstein (2015)(52)** | (14-14) | NI | (10-4) | 36.64 (6.85) | Exp/WS | Resting (passive) |
|  |  |  |  |  |  |  |
| **References** | **Sample**  **Initial-Final** | **Condition** | **Biological sex**  **(male-female)** | **Age (SD)** | **Design**  **Type** | **Control Intervention/Group** |
| **Lee et al. (2015)(53)** | (15-15) | University Students (moderate anxiety) | (12-3) | 27 | Exp/BS | Relaxation and CON |
| **Lehrer et al. (2006)(54)** | (45-36) | Adults (asthma) | (10-26) | 27.81 / 48.97^4^ | Exp/BS | Simplified HRVB intervention |
| **Lehrer et al. (2004)(55)** | (94-76) | Adults (asthma) | (30-64) | 37.3 (10.2) | Exp/BS | Simplified HRVB, EEG bioF., Waiting List |
| **Lehrer et al. (2018)(56)** | (68-55) | Adults (asthma) | (22-46) | 34 | Exp/BS | EEG biofeedback / Short vs Long HRVB |
| **Lehrer et al. (2003)(57)** | (57-54) | Healthy Adults | (16-38) | 29.24 | Exp/BS | Waiting List |
| **Lehrer et al. (2010)(58)** | (11-11) | Healthy Adults | (6-5) | 24.5 | Exp/BS | Slow Breathing + LPS exposition or not |
| **Lewis et al. (2015)(59)** | (891-891) | Healthy Adults | (850-41) | 23.82 (4.39) | Exp/BS | Stress management program |
| **Li et al. (2015)(60)** | (24-24) | Patients (stroke) | (13-11) | 57.01 | Exp/BS | Relaxation |
| **Lin (2018)(61)** | (96-82) | Healthy Adults | (19-63) | 27.9 | Exp/BS | Relaxation / CON |
| **Lin et al. (2015)(62)** | (154-127) | Patients (CAD) | (137-17) | 60.81 | Exp/BS | TAU + Waiting List |
| **Lin et al. (2012)(63)** | (45-43) | University Students (prehypertension) | (38-7) | 22.3 | Exp/BS | Slow Breathing / CON |
| **Lin et al. (2014)(64)** | (48-47) | University Students | (10-37) | 20.98 (1.03) | Exp/WS | Different HRVB protocols |
| **Lin et al. (2016)(65)^1^** | (9-9) | Heroin Users (depression) | (9-0) | 40.33 (8.5) | Exp/WS | Emotional Induction |
| **Lin et al. (2019)(66)** | (48-48) | Patients (depression) | (20-28) | 38 | Q-Exp | TAU |
| **Lin et al. (2020)(67)** | (60-57) | University Students | (12-45) | 23 | Exp/BS | Autogenic training |
| **Mackinnon et al. (2013)(68)** | (30-27) | Healthy Adults | (9-21) | 23 to 35 | Exp/WS | 2 emotional conditions |
| **Mason et al. (2019)(69)** | (86-86) | Patients (mental disorders) | (30-56) | 16 | Exp/BS | Waiting List |
| **McAusland and Addington (2018)(70)** | (20-19) | Adolescents and Adults (risk of psychosis) | (6-14) | 16.7 (2.3) | Q-Exp | No control Group |
| **Meier and Welch (2016)(71)** | (37-32) | University Students | (11-21) | 21.7 (3.1) | Exp/WS | Exercise / Quiet study |
| **Meule and Kübler (2017)(72)** | (66-65) | University Students | (0-65) | 21.2 (2.91) | Exp/BS | Breathing at 9b/m |
| **Meule et al. (2012)(73)** | (56-56) | University Students (food craving) | (9-47) | 24.12 (3.79) | Exp/BS | CON |
| **Meyer et al. (2018)(74)** | (20-12) | Adults (obesity) | (5-11) | 34.7 (6.5) | Exp/BS | Waiting List |
| **Minen et al. (2021)(75)** | (52-50) | Patients (migraine) | (5-42) | 42 | Exp/BS | Waiting List |
| **Munafo et al. (2016)(76)** | (40-31) | Healthy Adults | (31-0) | 47.8 | Exp/BS | Stress Diary |
| **Narita et al. (2018)(77)** | (38-38) | Pregnant Women | (0-38) | 32.4 | Q-Exp | CON |
| **Nelson Ferguson and Hall (2020)(78)** | (27-27) | University Students (Athletes) | (7-20) | 21.04 (2.85) | Exp/BS | Three types of HRVB protocols |
|  |  |  |  |  |  |  |
| **References** | **Sample**  **Initial-Final** | **Condition** | **Biological sex**  **(male-female)** | **Age (SD)** | **Design**  **Type** | **Control Intervention/Group** |
| **Nolan et al. (2005)(79)** | (46-42) | Patients (CHD) | (40-6) | 54.59 | Exp/BS | Stress management program |
| **Nolan et al. (2010)(80)** | (75-65) | Patients (hypertension) | (28-37) | 55.45 | Exp/BS | Relaxation |
| **Ozier and Linden. (2018)(81)** | (9-5) | Adults (brain tumor survivors) | Not Informed | Not Informed | Q-Exp | No control Group |
| **Pagaduan et al. (2019)(82)** | (10-10) | Healthy Adults | (10-0) | 28.7 (4.8) | Exp/WS | HRVB + 1b/m, Deep breathing |
| **Pagaduan et al. (2021)(83)** | (12-12) | Healthy Adults | (12-0) | 24 (5.02) | Exp/WS | Normal breathing |
| **Park and Jung (2020)(84)** | (30-24) | Patients (depression) | (12-18) | 28 | Exp/BS | TAU |
| **Patron et al. (2013)(85)** | (33-26) | Patients (after cardiac surgery) | (22-4) | 59.8 | Exp/BS | TAU |
| **Patron et al. (2020)(86)** | (30-30) | Healthy Adults | (30-0) | 49 | Exp/BS | HRVB alone or competitive |
| **Paul and Garg (2012)(87)** | (30-30) | Basketball Players | (17-13) | 21.13 (2.82) | Exp/BS | Watching video / CON |
| **Paul et al (2012)(88)** | (30-30) | Basketball Players | (17-13) | 21.70 (2.71) | Exp/BS | Watching video / CON |
| **Penzlin et al (2015)(89)** | (48-45) | Patients (AUD) | (34-14) | 42 (7.8) | Exp/BS | TAU |
| **Pérez-Gaido et al. (2021)(90)** | (15-10) | University Students | (7-8) | 25.8 (4.18) | Q-Exp | Free breathing |
| **Prinsloo et al. (2011)(91)** | (19-18) | Healthy Adults | (18-0) | 33.5 | Exp/BS | Active Control (non funtional device) |
| **Pyne et al. (2019)(92)** | (426-342) | Soldiers | (314-28) | 28.7 (8.4) | Exp/BS | CBM-I / CON |
| **Raymond et al. (2005)(93)** | (24-18) | Dance Students | (12-12) | 21.6 | Exp/BS | Neurofeedback / CON |
| **Reid et al. (2013)(94)** | (40-40) | Athletes | (23-17) | 16 to 61 | Q-Exp | No control Group |
| **Reiner (2008)(95)** | (24-20) | Patients (SO-A) | (10-10) | 18 to 65 | Q-Exp | No control Group |
| **Reneau (2020)(96)** | (10-10) | Patients (veterans with fibromyalgia) | (3-7) | 47 | Q-Exp | No control Group |
| **Rose et al (2021)(97)** | (40-40) | University Students | (21-19) | 22.31 (2.77) | Exp/BS | HRVB + inner balance / Watching movie |
| **Rusciano et al. (2017)(98)** | (20-20) | Soccer Players | (20-0) | 30.35 | Exp/BS | Motivational Videos and Music |
| **Russell et al. (2017)(99)** | (40-40) | University Students | (8-32) | 18.9 (1) | Exp/WS | 2 breathing ratio orders |
| **Sakakibara et al. (2013)(100)** | (45-42) | University Students | (14-31) | 22.8 (4.4) | Exp/BS | Autogenic Training / CON |
| **Schlatter et al (2021)(101)** | (44-44) | University Students | (22-22) | 22.25 (3.73) | Exp/BS | HRVB in no-stress condition / Watching a video |
| **Schmidt et al. (2012)(102)** | (20-20) | Patients (fibromyalgia) | (0-20) | 40.8 (13.7) | Q-Exp | No control Group |
| **Schmidt et al. (2013)(103)** | (22-22) | Patients (MMP or fibromyalgia) | (0-22) | 40.4 (13.4) | Q-Exp | No control Group |
| **Schuman and Killian (2019)(104)** | (12-11) | Patients (veterans with PTSD) | (9-3) | 36.16 (10.45) | Exp/BS | Deep breathing |
| **Schuman et al (2019)(105)** | (38-24) | Healthy Adults | (12-12) | 18-55 | Exp/BS | Mobile games |
| **Shaw et al. (2012)(106)** | (11-11) | Gymnasts | (0-11) | 19.9 (0.39) | Q-Exp | No control Group |
| **References** | **Sample**  **Initial-Final** | **Condition** | **Biological sex**  **(male-female)** | **Age (SD)** | **Design**  **Type** | **Control Intervention/Group** |
| **Sherlin et al. (2010)(107)** | (46-43) | Healthy Adults | (22-21) | 33.2 (8.77) | Exp/BS | Control Device |
| **Siepmann et al. (2008)(108)** | (38-38) | University Students + Patients (depression) | (13-25) | 28 (7.3) | Q-Exp | No breathing instruction^5^ |
| **Siepmann et al. (2014)(109)** | (59-48) | Patients (preterm labor) | (0-48) | 28.5 | Exp/BS | No breathing instruction |
| **Solarikova et al. (2016)(110)** | (20-20) | Healthy Adults + Patients (allergy) | (8-12) | Not Informed | Q-Exp | Healthy Controls |
| **Song and Lehrer (2003)(111)** | (5-5) | Healthy Adults | (0-5) | 29 | Exp/WS | Different breathing rates |
| **Sowder et al. (2010)(112)** | (30-27) | Children (FAP) | (14-16) | 13.5 | Q-Exp | Healthy Controls |
| **Stanton et al (2019)(113)** | (78-69) | Patients (SAD) | (0-69) | 25.42 (7.96) | Exp/BS | Autogenic training / Waiting list |
| **Steffen et al. (2017)(114)** | (95-95) | University Students | (38-57) | 20 | Exp/BS | RF+1 / CON |
| **Steffen et al. (2021)(115)** | (96-96) | University Students | (29-67) | 21 | Exp/BS | Deep breathing / Watching a video |
| **Stern et al. (2014)(116)** | (27-24) | Children (FAP or IBS) | (10-14) | 11.4 | Q-Exp | No control Group |
| **Stromberg et al. (2015) (117)** | (43-43) | University Students | (8-35) | 18 to 27 | Exp/BS | Normal breathing (12b/m) |
| **Sutarto et al. (2012)(118)** | (40-36) | Healthy Adults | (0-36) | 36.3 (10.14) | Exp/BS | Normal breathing |
| **Sutarto et al. (2013)(119)** | (40-36) | Healthy Adults | (0-36) | 36.3 (10.14) | Exp/BS | Breathing without biofeedback information |
| **Swanson et al. (2009)(120)** | (35-29) | Patients (heart failure) | (23-6) | 55.2 | Exp/BS | EEG biofeedback |
| **Taghizadeh et al (2019)(121)** | (44-44) | Patients (asthma) | (0-44) | 27 | Exp/BS | Self-relaxation with music |
| **Tan et al. (2011)(122)** | (30-30) | Veterans (PTSD) | (27-3) | 37.5 | Exp/BS | Healthy Controls / TAU |
| **Tan et al. (2013)(123)** | (34-27) | Veterans (pain + PTSD or depression) | (0-27) | 49.5 (10) | Q-Exp | No control Group |
| **Tatschi et al (2020)(124)** | (92-68) | Patients (depression) | 24-44) | 48.5 | Exp/BS | TAU |
| **Tavares et al. (2017)(125)** | (21-21) | University Students | (21-0) | 21.5 (2.5) | Q-Exp | No control Group |
| **Teeravisutkul et al (2019)(126)** | (35-35) | Patients (AUD) | (32-3) | 45 | Exp/BS | TAU |
| **Tsai et al. (2015)(127)** | (28-23) | Healthy Adults (insomnia) | Not Informed | 22.8 | Exp/WS | 2 breathing ratios 6 and 12b/m |
| **Vagedes et al. (2019)(128)** | (60-60) | Patients (women suffering dysmenorrhea) | (0-60) | 29,7 (8.0) | Exp/BS | Massage / TAU |
| **van de Zwan et al. (2015)(129)** | (126-75) | Healthy Adults | (20-55) | 26.2 | Exp/BS | Exercise / Meditation |
| **van de Zwan et al. (2019)(130)** | (50-40) | Healthy Adults (pregnant suffering stress) | (0-40) | 31.6 (5.9) | Exp/BS | Waiting list |
| **van Diest et al. (2014)(131)** | (30-23) | University Students | (2-28) | ~20 | Exp/WS | 4 HRVB protocols |
| **Wang et al. (2015)(132)** | (67-67) | Healthy Adults | (52-15) | 20 to 45 | Q-Exp | Non-RF breathing / +2 emotional conditions |
| **Wang et al. (2010)(133)** | (26-22) | Postmenopausal Women (prehypertension) | (0-22) | 52.55 (3.81) | Exp/BS | HRVB with another biofeedback |
| **Weeks et al. (2015)(134)** | (20-11) | Patients (pain) | (6-5) | 58.45 | Exp/BS | HRVB (reduced protocol) |
| **References** | **Sample**  **Initial-Final** | **Condition** | **Biological sex**  **(male-female)** | **Age (SD)** | **Design**  **Type** | **Control Intervention/Group** |
| **Wells et al. (2012)(135)** | (46-37) | Healthy Adults | (20-21) | 30.4 (11.98) | Exp/BS | Slow breathing / Reading |
| **Windthorst et al. (2017)(136)** | (28-24) | Patients (CFS) | (0-24) | 50.7 (9.3) | Exp/BS | Graded Exercise Training |
| **Wu et al. (2012)(137)** | (67-67) | Healthy Adults | (52-15) | 20 to 45 | Q-Exp | Non-stressed (employed) adults |
| **You et al. (2021)(138)** | (28-24) | University Students (Athletes) | (7-17) | 22 | Exp/WS | Watch a TV documentary |
| **Yu et al. (2018)(139)** | (210-134) | Patients (CAD) | (119-15) | 60.78 | Exp/BS | TAU |
| **Yucha et al. (2005)(140)** | (59-54) | Healthy Adults (hypertension) | (17-37) | 47.5 (10.3) | Q-Exp | No control Group |
| **Zauszniewski et al. (2013)(141)** | (20-20) | Healthy Adults | (0-20) | 58 | Q-Exp | No control Group |
| **Zucker et al. (2009)(142)** | (53-38) | Substance abuse Users (PTSD) | (21-17) | 18 to 60 | Exp/BS | Progressive Muscle Relaxation |
| **Zunhammer et al. (2013)(143)** | (20-19) | University Students | (10-10) | 24.4 | Exp/WS | 3 HRVB protocols / Free breathing |

s=Study; CON=passive control; LPS=Lipopolysaccharide; SAB=Slow Abdominal Breathing; TAU=Treatment As Usual.
Agenesis in the corpus callosum (ACC); Acute Ischemic Stroke (AIS); Alcohol Use Disorder (AUD); Coronary Artery bypass graft (CABG); Chronic Fatigue Syndrome (CFS); Chronic Obstructive Pulmonary Disease (COPD); Complex Regional Pain Syndrome (CRPS); Coronary Artery Disease (CAD); Coronary Heart Disease (CHD); Cognitive bias modification for interpretation (CBM-I); Functional Abdominal Pain (FAP); Irritable Bowel Syndrome (IBS); Intellectual disability (ID); Masticatory and Myofascial Pain (MMP); Post-Traumatic Stress Disorder (PTSD); Recurrent Abdominal Pain (RAP); Sexual Arousal Disorder (SAD); Substance Use Disorder (SUD); Sympathetic Over-Arousal (SO-A); Traumatic Brain Injury (TBI).
 ^1^=in brackets study 2; ^2^=subclinical psychotic report; ^3^=SEM (standard error of the mean); ^4^=two groups of age; ^5^=only for the health participants.

**Supplementary Table 6:** HRVB protocol of the included studies (n=143, interventions n=145)

| References | Type of intervention | RF Range (mean) / Preset RF (actual) (b/m) | (Inhalation/Holding/ Exhalation) | Weeks^4^ | Sessions Lab | Min. Lab  (breathing) | Sessions Home  (days) | Min. Home (breathing)^8^ |
| --- | --- | --- | --- | --- | --- | --- | --- | --- |
| Allen and Friedman (2012)(1) | Preset-Pace | 6 (NI) | 4.5/x/5.5 | 1 | 1 | 3min | Only Lab. | NA |
| Amichai et al. (2019)(2) | Preset-Pace | 6 (NI) | 4/x/6 | 1 | 1 | 15min^6^ | Only Lab. | NA |
| Amjadian et al. (2020)(3) | Individual | Individualized (NI) | NI | 8 | 8 | 120min^6^ | 56 | NI |
| Bartur et al. (2014)(4) | Preset-Pace | 6 (8.25) | NI | 1 | 1 | 5min | Only Lab. | NA |
| Bates et al. (2019)(5) | Preset-Pace | 6 (controlled) | NI | 1 | 1 | 5min | Only Lab. | NA |
| Beckham et al. (2013)(6) | Individual | Individualized (NI) | NI | 1 | 1 | 30-60min^6^ | Yes, free | NI |
| Brinkmann et al. (2020)(7) | Optimal RF | 4.5 to 7.0 (NI) | NI | 6 | 6 | 5min | 42 | ~20min |
| Burch et al. (2020)(8) | Individual | Individualized (NI) | NI | 4 to 6 | 4 to 6 | 25+15min | Daily | 15min |
| Caldwell et al. (2018)(9) | Optimal RF | 6.5, 6.0, 5.5, 5.0, 4.5 (NI) | NI | 4 | 5 | 20min | 12 to 15 | 15-20min |
| Chalaye et al. (2009/1)(10) | Individual | Individualized (5.7±0.19) | NI | 1 | 1 | 60min^6^ | Only Lab. | NA |
| Chalaye et al. (2009/2)(10) | Pace-Preset | 6 (5.78±0.08) | NI | 1 | 1 | 60min^6^ | Only Lab. | NA |
| Chang et al. (2020)(11) | Pace-Preset | 6 (NI) | NI | 1 | 4 | 20min | 56 | 10min |
| Chelidoni et al. (2020)(12) | Pace-Preset | 6 (NI) | NI | 1 | 1 | 5min | Only Lab. | NA |
| Chen et al. (2016/1)(13) | Referenced | Referenced (NI) | NI | 8 | 15 | 30min | 56 | 2x30min |
| Chen et al. (2016/2)(13) | Pace-Preset | 6 (NI) | NI | 8 | 15 | 30min | 56 | 2x30min |
| Cheng et al. (2019)(14) | Pace-Preset | 6 (NI) | 5/x/5 | 1 | 1 | 5, 7 or 9min | Only Lab. | NA |
| Clamor et al. (2016)(15) | Preset-Pace +Individual | 6  (16 part.<7; 8 part. (7-9), 4 part.>10) | 4/x/6 | 1 | 1 | 10+10min | Only Lab. | NA |
| Climov et al (2014)(16) | Pace-Preset | 6 (NI) | 3/0.1/6.9 | 4 | 8 | 15min | 28 | NI |
| Cullins et al (2013)(17) | Individual | Individualized (NI) | NI | 2 | 1 | 45min^6^ | 14 | 20min |
| De Couck et al. (2019)(s2)(18) | Preset-Pace | 4.3 (NI) | 5/2/7 | 1 | 1 | 2min | Only Lab. | NA |
| de Zambotti et al. (2019)(19) | Preset-Pace | 6 (NI) | NI | 1 | 2 | 10min | Only Lab. | NA |
| del Paso et al. (2006)(20) | Preset-Pace | 6 (6,33) | 3/1/6 | 1 | 1 | 15-25min | Only Lab. | NA |
| del Pozo et al. (2004)(21) | Individual | Individualized (NI) | NI | 6 | 6 | 45min | 42 | 20min |
| Deschodt-Arsac et al. (2018)(22) | Preset-Pace | 6 (NI) | NI | 5 | Only Home | NA | 35 | 2x5min |
| Deschodt-Arsac et al. (2020)(23) | Preset-Pace | 6 (NI) | NI | 5 | 10 | 5min | Only Lab. | NA |
| Dessy et al. (2020)(24) | Preset-Pace | 6 (NI) | NI | 4 | 8 | 15min | Only Lab. | NA |
| Dziembowska et al. (2016)(25) | Individual | Individualized (NI) | NI | 3 | 10 | 5min | Only Lab. | NA |
| References | **Type of intervention** | **RF Range (mean) / Preset RF (actual) (b/m)** | **(Inhalation/Holding/ Exhalation)** | **Weeks^4^** | **Sessions Lab** | **Min. Lab  (breathing)** | **Sessions Home  (days)** | **Min. Home (breathing)^8^** |
| Ebben et al. (2009)(26) | Individual | Individualized (NI) | NI | 1 | 1 | 10+20min | Only Lab. | NA |
| Eddie et al. (2018)(27) | Optimal RF | 4.5, 5.0, 5.5, 6.0, 6.5 (NI) | NI | 12 | 8 | 15min | 42 | 2x15min |
| Eddie et al. (2014)(28) | Optimal RF | 4.5, 5, 5.5, 6, 6.5 (NI) | NI | 3 | 3 | 60-75^6^ | 21 | 2x20min |
| Francis et al. (2016)(29) | Preset-Pace | 6 (7,65±2,21) | NI | 1 | 1 | 5min | Only Lab. | NA |
| Francis et al. (2016)(30) | Preset-Pace | 6 (7.06±1.31) | NI | 1 | 1 | 5min | Only Lab. | NA |
| Giardino et al. (2004)(31) | Individual | Individualized (NI) | NI | 5 | 5 | 5-10min | 35 | 20min |
| Gray et al. (2019)(32) | Individual | Individualized (~7.5) | 4/x/4 | 4 | 8 | 10min | Only Lab. | NA |
| Gross et al. (2016)(33) | Referenced | Referenced (~5.5) | 33%/x/66%^3^ | NI | 4 | 5min | Daily | 5min |
| Gruzelier et al. (2014)(34) | Optimal RF | 0,04-0,15hz (NI) | NI | NI | 8 to 10 | 20min | Only Lab. | NA |
| Hallman et al. (2011)(35) | Optimal RF | 4.5, 5, 5.5, 6, 6.5 (random) (5.7) | NI | 10 | 10 | 4x5min | 50 | 15min |
| Hassett et al. (2007)(36) | Optimal RF | 6.5, 6, 5.5, 5, 4,5 (NI) | NI | 10 | 10 | 20min | 70 | 2x20min |
| Hasuo et al. (2020)(37) | Optimal RF | 5.0, 5.5, 6.0, 6.5, 7.0 (NI) | NI | 2 | 2 | 30min | 7 | 5-30min |
| Hasuo et al. (2019)(38) | Optimal RF | 5.0, 5.5, 6.0, 6.5, 7.0 (NI) | NI | 4 | 3 | 30min | 28 | 5-20min |
| Hasuo et al. (2020)(39) | Optimal RF | 5.0, 5.5, 6.0, 6.5, 7.0 (NI) | NI | 4 | 3 | 15min | 28 | 5-20min |
| Hasuo et al. (2018)(40) | Optimal RF | 5.0, 5.5, 6.0, 6.5, 7.0 (6 / 6.5) | NI | 4 | NI | 30min | 28 | 5min |
| Henriques et al. (2011 /s2)(41) | Individual | Individualized (NI) | NI | 4 | 1 | NI^7^ | 20 | 15min |
| Hsieh et al. (2020)(42) | Preset-Pace | 6 (NI) | NI | 6 | 6 | 60min^6^ | Only Lab. | NA |
| Huang et al. (2019)(43) | Preset-Pace | NI | NI | 12 | 3 | 15min^6^ | 84 | 15min |
| Hunter et al. (2019)(44) | Preset-Pace | 6 (NI) | 4/x/6 | 1 | 1 | 5min | Only Lab. | NA |
| Jester et al. (2019)(45) | Preset-Pace | NI | NI | 3 | 6 | 30min^6^ | 6 | NI |
| Karavidas et al. (2007)(46) | Optimal RF | 6.5, 6, 5.5, 5, 4,5 (NI) | NI | 10 | 10 | 30min | 70 | 2x20min |
| Kohlenberg et al. (2020)(47) | Preset-Pace | 6 (NI) | NI | 1 | 1 | 5min | Only Lab. | NA |
| Kennedy and Pretorious (2008)(48)^1^ | Individual | Individualized (NI) | NI | 9 | 1 | 30min^6^ | 45 | 4x5min |
| Kudo et al. (2014)(49) | Individual | Individualized (NI) | NI | 4 | NI | NI | 28 | NI |
| Laborde et al. (2017)(50) | Preset-Pace | 6 (NI) | 4.5/x/5.5 | 1 | 1 | 17min | Only Lab. | NA |
| Laborde et al. (2019)(51) | Preset-Pace | 6 (NI) | 4.5/x/5.5 | 1 | 1 | 17min | Only Lab. | NA |
| Lee and Finkelstein (2015)(52) | Individual | Individualized (NI) | NI | 1 | 1 | 10min | Only Lab. | NA |
|  |  |  |  |  |  |  |  |  |
| References | **Type of intervention** | **RF Range (mean) / Preset RF (actual) (b/m)** | **(Inhalation/Holding/ Exhalation)** | **Weeks^4^** | **Sessions Lab** | **Min. Lab  (breathing)** | **Sessions Home  (days)** | **Min. Home (breathing)^8^** |
| Lee et al. (2015)(53) | Individual | Individualized (NI) | NI | 8 | 4 | 45min^6^ | 56 | 3x5-10min |
| Lehrer et al. (2006)(54) | Referenced | Referenced (NI) | NI | NI | 10 | 30min | Only Lab. | NA |
| Lehrer et al. (2004)(55) | Referenced | Referenced (6) | NI | 10 | 10 | NI | 70 | 2x20min |
| Lehrer et al. (2018)(56) | Referenced | Referenced (NI) | NI | 10 | 6 or 10 | NI | 70 | 2x20min |
| Lehrer et al. (2003)(57) | Optimal RF | 4.5, 5, 5.5, 6, 6.5 (6) | NI | 10 | 10 | 30min | 49 | 2x20min |
| Lehrer et al. (2010)(58) | Optimal RF | 4.5, 5, 5.5, 6, 6.5, 7 (6) | NI | 1+1 | 4 (preexpo) / 6 (LPS) | 20(preexpo)/ 10(LPS) | 14 (preexpo) | 2x20min |
| Lewis et al. (2015)(59) | Preset-Pace | 6 (NI) | 5/x/5 | NI | NI | 15min | Only Lab. | NA |
| Li et al. (2015)(60) | Referenced | Referenced (NI) | NI | 4 to 6 | 12 to 28 | 30min | Only Lab. | NA |
| Lin (2018)(61) | Optimal RF | 6.5, 6.0, 5.5, 5.0, 4.5 (NI) | NI | 3 | 3 | 60min^6^ | 21 | 10min |
| Lin et al. (2015)(62) | Referenced | Referenced (NI) | NI | 6 (no follow-up) | 6 | 60min^6^ | 42 | 10min |
| Lin et al. (2012)(63) | Optimal RF | 6.5, 6, 5.5, 5, 4,5 (5,31±0,37) | NI | 5 | 10 | NI | 35 | 2x20min |
| Lin et al. (2014)(64) | Preset-Pace | 6 / 5,5 (NI) | 40-50%/x/50-60%^3^ | 1 | 1 | 12min | Only Lab. | NA |
| Lin et al. (2016)(65)^1^ | Referenced | Referenced (NI) | NI | 6 | 6 | NI | NI | NI |
| Lin et al. (2019)(66) | Referenced | Referenced (5.5 to 6.0) | NI | 6 | 6 | 60min | 42 | 10min |
| Lin et al. (2020)(67) | Preset-Pace | 12 to 8 to 6 | NI | 1 | 1 | 60min | Only Lab. | NA |
| Mackinnon et al. (2013)(68) | Preset-Pace | 6 (NI) | NI | 1 | 1 | 5min | Only Lab. | NA |
| Mason et al. (2019)(69) | Preset-Pace | 6 (NI) | 5/x/5 | 1 | 1 | 30min | Only Lab. | NA |
| McAusland and Addington (2018)(70) | Individual | Individualized (NI) | NI | 4 | Only Home | NA | Yes, free | 60min per week |
| Meier and Welch (2016)(71) | Preset-Pace | 6 (68% at 6b/m) | NI | 1 | 1 | 8min | Only Lab. | NA |
| Meule and Kübler (2017)(72) | Preset-Pace | 6 (NI) | NI | 1 | 1 | 10min | Only Lab. | NA |
| Meule et al. (2012)(73) | Referenced | Referenced (NI) | NI | 4 | 12 | 20min | Only Lab. | NA |
| Meyer et al. (2018)(74) | Referenced | Referenced (NI) | Shorter/x/Longer | 6 | 6 | NI | 42 | 2x10min |
| Minen et al. (2021)(75) | Individual | Individualized (NI) | NI | 8.5 | Only Home | NA | 60 | 10min |
| Munafo et al. (2016)(76) | Individual | Individualized (NI) | NI | 5 | 5 | 2x6min | Only Lab. | NA |
| Narita et al. (2018)(77) | Individual | Individualized (NI) | NI | 3 to 4 | Only Home | NA | 21 to 28 | free |
| Nelson Ferguson and Hall (2020)(78) | Optimal RF | 7.5, 7. 6.5, 6, 5.5 (between 6-7) | NI | 2.5 (up to 8) | 5 | 15min | Only Lab. | NA |
| Nolan et al. (2005)(79) | Preset-Pace | 6 (only in 8 part. <0,12Hz) | NI | 4 | 5 | 90min^6^ | Only Lab. | NA |
| Nolan et al. (2010)(80) | Preset-Pace | 6 (NI) | NI | 8 | 6 | 30min | 56 | 20min |
| References | **Type of intervention** | **RF Range (mean) / Preset RF (actual) (b/m)** | **(Inhalation/Holding/ Exhalation)** | **Weeks^4^** | **Sessions Lab** | **Min. Lab  (breathing)** | **Sessions Home  (days)** | **Min. Home (breathing)^8^** |
| Ozier and Linden. (2018)(81) | Referenced | Referenced (NI) | NI | 8 | 8 | 10min | 56 | 20min |
| Pagaduan et al. (2019)(82) | Optimal RF | 5.44 (0.88) | NI | 1 | 1 | 10+10min | Only Lab. | NA |
| Pagaduan et al. (2021)(83) | Optimal RF | 4.4 to 7.4 (0.09hz) | NI | 1 | 1 | NI | Only Lab. | NA |
| Park and Jung (2020)(84) | Referenced | Referenced (NI) | shorter/x/longer | 4 | 6 | 20min | 28 | 20min |
| Patron et al. (2013)(85) | Individual | Individualized (NI) | NI | 2 | 5 | 45min^6^ | 14 | 2x15min |
| Patron et al. (2020)(86) | Individual | Individualized (NI) | shorter/x/longer | 5 | 5 | 12min | Only Lab. | NA |
| Paul and Garg (2012)(87) | Optimal RF | 6.5, 6, 5.5, 5, 4.5 (6) | NI | 1,5 | 10 | 20min | Only Lab. | NA |
| Paul et al (2012)(88) | Optimal RF | 6.5, 6, 5.5, 5, 4.5 (6.25) | NI | 1,5 | 10 | 20min | Only Lab. | NA |
| Penzlin et al (2015)(89) | Preset-Pace | 6 (NI) | NI | 2 | 6 | 20min | Only Lab. | NA |
| Pérez-Gaido et al. (2021)(90) | Optimal RF | 7.0, 6.5, 6, 5.5, 5.0 (5.7) | equal | 3 | 3 | 2x10min+2min | Only Lab. | NA |
| Prinsloo et al. (2011)(91) | Individual | Individualized (6±1) | NI | 1 | 1 | 10min | Only Lab. | NA |
| Pyne et al. (2019)(92) | Individual | Individualized (NI) | NI | NI | 1 | NI | 3x week | Few minutes |
| Raymond et al. (2005)(93) | Referenced | Referenced (NI) | NI | NI | 10 | 20min | NI | NI |
| Reid et al. (2013)(94) | Optimal RF | Individualized (7,5 to 6) | 3-4/x/5-6 | NI | NI | 5min | Only Lab. | NA |
| Reiner (2008)(95) | Individual | Individualized (NI) | NI | 3 | 1 | NI | 21 | 20min |
| Reneau (2020)(96) | Individual | Individualized (NI) | NI | 8 | 8 | 20min | 56 | 2x20min |
| Rose et al (2021)(97) | Individual | Individualized (NI) | NI | 4 | 4 | 6min | Only Lab. | NA |
| Rusciano et al. (2017)(98) | Optimal RF | 4.5, 5, 5.5, 6, 6.5 (NI) | NI | 7 to 8 | 15 | 15min | 49 to 56 | 5 to 20min |
| Russell et al. (2017)(99) | Preset-Pace | 6 (NI) | 4-5/x/2-5 | 1 | 1 | 2x6min | Only Lab. | NA |
| Sakakibara et al. (2013)(100) | Individual | Individualized (NI) | NI | NI | NI | NI | 2 | NA |
| Schlatter et al (2021)(101) | Individual | Individualized (6 as reference) | NI | 1 | 1 | 15min | Only Lab. | NA |
| Schmidt et al. (2012)(102) | Preset-Pace | 6 (NI) | NI | 2 | 1 | 15min | 14 | 3x10min |
| Schmidt et al. (2013)(103) | Preset-Pace | 6 (NI) | 5/x/5 | 2 | NI | NI | 14 | 3x10min |
| Schuman and Killian (2019)(104) | Optimal RF | 6.5, 6.0, 5.5, 5.0, 4.5, 4.0 | NI | 4 | Only Home | NA | 28 | 2x10-15min |
| Schuman et al (2019)(105) | Optimal RF | 7.0, 6.0, 5.0, 4.5, 4.0 | NI | 8 | 8 | NI | 32 | NI |
| Shaw et al. (2012)(106) | Preset-Pace | 6 (NI) | NI | 5 | 10 | 3x1.5min | Only Lab. | NA |
| Sherlin et al. (2010)(107) | Individual | Individualized (NI) | NI | 1 | 1 | 15min | Only Lab. | NA |
| Siepmann et al. (2008)(108) | Individual | Individualized (NI) | NI | 2 | 6 | 25min | Only Lab. | NA |
| References | **Type of intervention** | **RF Range (mean) / Preset RF (actual) (b/m)** | **(Inhalation/Holding/ Exhalation)** | **Weeks^4^** | **Sessions Lab** | **Min. Lab  (breathing)** | **Sessions Home  (days)** | **Min. Home (breathing)^8^** |
| Siepmann et al. (2014)(109) | Individual | Individualized (NI) | NI | 2 | 6 | 25min | Only Lab. | NA |
| Solarikova et al. (2016)(110) | Individual | Individualized (NI) | NI | 4 to 5 | 18 | 20min | Only Lab. | NA |
| Song and Lehrer (2003)(111) | Preset-Pace | 3 / 4 / 6 / 8 / 10 / 12 / 14 (NI) | NI | NI | 21 to 24 | 5min x preset | Only Lab. | NA |
| Sowder et al. (2010)(112) | Referenced | Referenced (NI) | NI | 8 | 6 | NI | 56 | 10min |
| Stanton et al (2019)(113) | Individual | Individualized (6 as a reference) | NI | 4 | 3 | 50-90min^6^ | 10 | 20min |
| Steffen et al. (2017)(114) | Optimal RF | 7, 5, 6.5, 5.5, 6 (NI) | NI | 1 | 1 | 15+15min | Only Lab. | NA |
| Steffen et al. (2021)(115) | Preset-Pace | 6 (12.2 (4.3)) | NI | 1 | 1 | 10min | Only Lab. | NA |
| Stern et al. (2014)(116) | Individual | Individualized (NI) | NI | NI | 8 (3 to 19) | 30min^6^ | Daily | 20min |
| Stromberg et al. (2015) (117) | Preset-Pace | 6 (12 part.=7; 9 part. >7) | NI | 1 | 1 | 5min | Only Lab. | NA |
| Sutarto et al. (2012)(118) | Optimal RF | 6.5, 6.0, 5.0, 4.5 (6.3) | NI | 5 | 5 | 20min | 35 | 20min (5min x4) |
| Sutarto et al. (2013)(119) | Optimal RF | 6.5, 6.0, 5.0, 4.5  (higher at the beginning) | NI | 5 | 5 | NI | 35 | 20-30min |
| Swanson et al. (2009)(120) | Individual | Individualized (NI) | NI | 6 | 6 | 45min^6^ | 42+126 (follow-up) | 20min (minim) |
| Taghizadeh et al (2019)(121) | Individual | Individualized (6 as a reference) | NI | 1 | 1 | 20min | Only Lab. | NA |
| Tan et al. (2011)(122) | Optimal RF | 6.5, 6, 5.5, 5, 4.5 (NI) | NI | 8 | 8 | 20min | 56 | 20min (2xday) |
| Tan et al. (2013)(123) | Individual | Individualized (7 to 5,5) | NI | 6+6 | 4 | NI | 42 | NI |
| Tatschi et al (2020)(124) | Preset-Pace | 6 (5.5 (0.46)) | 5/x/5 | 5 | 5 | 35min | 35 | 2x10min |
| Tavares et al. (2017)(125) | Preset-Pace | 6 (NI) | NI | 1 | 1 | 10min | Only Lab. | NA |
| Teeravisutkul et al (2019)(126) | Individual | Individualized (NI) | NI | 4 | 16 | 30min | Only Lab. | NA |
| Tsai et al. (2015)(127) | Preset-Pace | 6 or 12 (NI) | 3-7/x/3-7 | 1+1 | 1+1 | 5min | 7+7 | 20min |
| Vagedes et al. (2019)(128) | Individual | Individualized (NI) | NI | 12 | Only Home | NA | 84 | 15min |
| van de Zwan et al. (2015)(129) | Individual | Individualized (NI) | NI | 5 | 1 | NI | 35 | 10-20min |
| van de Zwan et al. (2019)(130) | Optimal RF | 6.5, 6.0, 5.5, 5.0, 4.5 (NI) | NI | 5 | 5 | 60-90min^6^ | 35 | 10-2x20min |
| van Diest et al. (2014)(131) | Preset-Pace | 6 or 12 (~7.5 / ~12.3) | 3-7/x/3-7 and 1.5-3.5/x/1.5-3.5 | 1 | 1 | 4x5min | Only Lab. | NA |
| Wang et al. (2015)(132) | Optimal RF | 4 to 7 (NI) | NI | 3 | 3 | NI | 21 | 4x5min |
| Wang et al. (2010)(133) | Individual | 6 (NI) | NI | 3.5 | 10 | 20min | ~25 | 2x20min |
| Weeks et al. (2015)(134) | Individual | Individualized (NI) | NI | 3 | 9 | 2x10min | Only Lab. | NA |
| Wells et al. (2012)(135) | Referenced | Referenced (NI) | NI | 1 | 1 | 30min | Only Lab. | NA |
| References | **Type of intervention** | **RF Range (mean) / Preset RF (actual) (b/m)** | **(Inhalation/Holding/ Exhalation)** | **Weeks^4^** | **Sessions Lab** | **Min. Lab  (breathing)** | **Sessions Home  (days)** | **Min. Home (breathing)^8^** |
| Windthorst et al. (2017)(136) | Individual | Individualized (10 to 6) | NI | 8 | 8 | 20-30min | 56 | 2x 5 to 10min |
| Wu et al. (2012)(137) | Individual | Individualized (7 to 4) | NI | 3 | 3 | NI | Yes, free | 2x20min |
| You et al. (2021)(138) | Preset-Pace | 6 (researcher controlled it) | 4.5/x/5.5 | 1 | 1 | 15min | Only Lab. | NA |
| Yu et al. (2018)(139) | Referenced | Referenced (NI) | NI | 6 | 6 | 60min^6^ | Only Lab. | NA |
| Yucha et al. (2005)(140) | Individual | Individualized (7 to 4) | NI | 8 | 4 | NI | Only Lab. | NA |
| Zauszniewski et al. (2013)(141) | Individual | Individualized (NI) | NI | 4 | 1 | 2x10min | Only Lab. | NA |
| Zucker et al. (2009)(142) | Individual | Individualized (6) | NI | 4 | 1 | 10min | 28 | 20min |
| Zunhammer et al. (2013)(143) | Preset-Pace | 3.6 / 6 / 8.4 / Free (3.7 / 6 / 8.5) | half/x/double | 4 | 4 | 57min | Only Lab. | NA |

s=Study; LPS=Lipopolysaccharide; NI=Not Informed; NA=Not Applicable; part.=participant
^1^=In one intervention, they combined two types of breathing protocols; ^2^=In brackets study 2; ^3^=Percentage of time inhaling and exhaling; ^4^=When there are two added numbers “+” it includes the intervention and the follow-up phases; ^5^=Two experimental conditions: x=experimental, c=control; ^6^=Only the time of the whole intervention session, not specific breathing time, was indicated in the study; ^7^=Until feeling comfortable; ^8^=Number of times per day x minutes each time.

**Supplementary Table 7:** Conditions of the HRVB interventions of the included studies (n=143, interventions n=145)

| References | Conditions during intervention | Previous Recommendation: Avoid… |
| --- | --- | --- |
| Allen and Friedman (2012)(1) | Open Eyes | Caffeine, Alcohol, Exercise (12h3), Smoking (2h), Food (1h) |
| Amichai et al. (2019)(2) | Open Eyes, Sitting | NI |
| Amjadian et al. (2020)(3) | Open Eyes, home practice before going to sleep | NI |
| Bartur et al. (2014)(4) | Quiet Room, 21-26ºC, Sitting, Open Eyes | Food, Caffeine, Exercise (4h) |
| Bates et al. (2019)(5) | Open Eyes | Alcohol, drugs (24h) |
| Beckham et al. (2013)(6) | Open Eyes, 1 participant | NI |
| Brinkmann et al. (2020)(7) | Open Eyes | Caffeine, Alcohol, Tobacco (the day of assessment) |
| Burch et al. (2020)(8) | Open Eyes | NI |
| Caldwell et al. (2018)(9) | Open Eyes | Exercise, Coffee, Tobacco (3h) |
| Chalaye et al. (2009/1)(10) | Quiet Room, Sitting, Open Eyes | NI |
| Chalaye et al. (2009/2)(10) | Quiet Room, Sitting, Open Eyes | NI |
| Chang et al. (2020)(11) | Supine Position, Open Eyes, 1 participant | NI |
| Chelidoni et al. (2020)(12) | Open Eyes | NI |
| Chen et al. (2016/1)(13) | Light Attenuated, Quiet Room, 26±2ºC | Food and Drink 2h, and Caffeine each training day |
| Chen et al. (2016/2)(13) | Light Attenuated, Quiet Room, 26±2ºC | Food and Drink 2h, and Caffeine each training day |
| Cheng et al. (2019)(14) | Sitting, Controlled light, Open Eyes | NI |
| Clamor et al. (2016)(15) | Sitting, Open Eyes | Cigarettes (30min), Caffeine (30min), alcohol (24h) or drugs (1 week) And report the amount of sport, and meditation and relax techniques practice regularly. |
| Climov et al (2014)(16) | In the morning, 1 or 2 participants | NI |
| Cullins et al (2013)(17) | Open Eyes | NI |
| De Couck et al. (2019)(s2)(18) | Open Eyes | NI |
| de Zambotti et al. (2019)(19) | Supine Position, Light controlled by participant, Quiet Room, Temperature Controlled, Open Eyes, Before Sleeping | Caffeine and Alcohol (3h) |
| del Paso et al. (2006)(20) | Sitting, Open Eyes | NI |
| del Pozo et al. (2004)(21) | Open Eyes, At the same time of day | Caffeine, Alcohol and Vigorous Exercise (4h.) |
| Deschodt-Arsac et al. (2018)(22) | Quiet Room, Sitting, 8:00 to 10:00 and 19:00 to 22:00 | Daily Diary of Training, Diet, Sleep and Fatigue; and Food and Drink (2h) |
| Deschodt-Arsac et al. (2020)(23) | Quiet Room, Sitting, Open Eyes, Morning and Evening | Food and Drink (2h) |
| Dessy et al. (2020)(24) | Open Eyes, Morning | NI |
| Dziembowska et al. (2016)(25) | Open Eyes, 15:00 to 19:00 | NI |
| References | **Conditions during intervention** | **Previous Recommendation: Avoid…** |
| Ebben et al. (2009)(26) | Open Eyes, At night (from 20:00) | Caffeine after 2pm |
| Eddie et al. (2018)(27) | Open Eyes | NI |
| Eddie et al. (2014)(28) | Open Eyes | NI |
| Francis et al. (2016)(29) | Sitting, Open Eyes, 1 participant | NI |
| Francis et al. (2016)(30) | Sitting, Open Eyes, 1 participant | NI |
| Giardino et al. (2004)(31) | Open Eyes | NI |
| Gray et al. (2019)(32) | Open Eyes, 1 participant | NI |
| Gross et al. (2016)(33) | Open Eyes | Sleep, Food, and Caffeine were reported |
| Gruzelier et al. (2014)(34) | Sitting, Open Eyes, 8:45 to 10:15 | NI |
| Hallman et al. (2011)(35) | Light Attenuated, 23ºC, Sitting, Open Eyes, 8:00 to 10:00 or 13:00 or 15:00 | Smoking, Caffeine, Food and Drink (2h) |
| Hassett et al. (2007)(36) | 21-24ºC, Sitting, Open Eyes, At the same time of day | Caffeine and Alcohol (12h) |
| Hasuo et al. (2020)(37) | Open Eyes | NI |
| Hasuo et al. (2019)(38) | Open Eyes, Bedtime | NI |
| Hasuo et al. (2020)(39) | Open Eyes, Bedtime | NI |
| Hasuo et al. (2018)(40) | Open Eyes | NI |
| Henriques et al. (2011 /s2)(41) | Open Eyes, the time participants prefer, 1 participant | NI |
| Hsieh et al. (2020)(42) | NI | NI |
| Huang et al. (2019)(43) | Sitting, Quiet Room, Temperatures controlled, Open Eyes | NI |
| Hunter et al. (2019)(44) | Sitting, Open Eyes | NI |
| Jester et al. (2019)(45) | Open Eyes | NI |
| Karavidas et al. (2007)(46) | Light Attenuated, Temperature controlled, semi reclined, Open Eyes | NI |
| Kohlenberg et al. (2020)(47) | Open Eyes | NI |
| Kennedy and Pretorious (2008)(48)^1^ | Open Eyes | NI |
| Kudo et al. (2014)(49) | Open Eyes | NI |
| Laborde et al. (2017)(50) | Sitting, Closed Eyes, 3 to 6 participants | Sleep, Food and Drink (2h) and Exercise (24h) |
| Laborde et al. (2019)(51) | Open Eyes | Food and Drink (2h) and Exercise (24h) |
| Lee and Finkelstein (2015)(52) | Open Eyes, 1 participant | No medication, Sleep well (24h), No food (2h), Caffeine and Tobacco (6h) |
| Lee et al. (2015)(53) | Open Eyes, 1 participant | NI |
|  |  |  |
| References | **Conditions during intervention** | **Previous Recommendation: Avoid…** |
| Lehrer et al. (2006)(54) | Open Eyes | NI |
| Lehrer et al. (2004)(55) | NI | NI |
| Lehrer et al. (2018)(56) | NI | NI |
| Lehrer et al. (2003)(57) | Open Eyes | NI |
| Lehrer et al. (2010)(58) | Open Eyes, 9:00 to 18:00 | Caffeine until midnight, and Alcohol, Caffeine and Tobacco |
| Lewis et al. (2015)(59) | Sitting, Open Eyes, up to 20 participants | Chewing gum (during HRVB), Tobacco and Caffeine that day |
| Li et al. (2015)(60) | NI | NI |
| Lin (2018)(61) | 22-24ºC, Sitting, Open Eyes | Caffeine, Alcohol (3h) and wash their hair the day before |
| Lin et al. (2015)(62) | Light Attenuated, Temperature controlled, Sitting, Open Eyes, 9:00 to 17:00 | Excessive Exercise, Alcohol, Caffeine, Smoking (3h) |
| Lin et al. (2012)(63) | 21ºC, 60% of humidity, At sea level, Open Eyes, At the same time of day | NI |
| Lin et al. (2014)(64) | Light Attenuated, 24-26ºC, Sitting, Open Eyes, 9:00 to 17:00 | Excessive Exercise, Alcohol, Caffeine, Smoking (3h) |
| Lin et al. (2016)(65)^1^ | Light Attenuated, Temperature Controlled, Sitting, Open Eyes | Excessive Exercise, Alcohol, Caffeine, Smoking (3h) |
| Lin et al. (2019)(66) | NI | NI |
| Lin et al. (2020)(67) | Open Eyes | NI |
| Mackinnon et al. (2013)(68) | Eyes closed | NI |
| Mason et al. (2019)(69) | Open Eyes | NI |
| McAusland and Addington (2018)(70) | Open Eyes | NI |
| Meier and Welch (2016)(71) | Sitting, Open Eyes, At the same time of day | Alcohol (24h), Caffeine, Exercise (2h) |
| Meule and Kübler (2017)(72) | Open Eyes, 8:00 to 18:30, 1 participant | Food and Caffeine (1h) |
| Meule et al. (2012)(73) | Quiet Room, Open Eyes | Food, Smoking and Caffeine (1h) |
| Meyer et al. (2018)(74) | Open Eyes | NI |
| Minen et al. (2021)(75) | Open Eyes | NI |
| Munafo et al. (2016)(76) | Light Attenuated, Quiet Room, 21ºC, Sitting, Open Eyes | Alcohol, Caffeinated beverage and Smoking (3h) |
| Narita et al. (2018)(77) | Open Eyes, At night | NI |
| Nelson Ferguson and Hall (2020)(78) | Sitting, Open Eyes | NI |
| Nolan et al. (2005)(79) | Open Eyes | NI |
| Nolan et al. (2010)(80) | NI | NI |
| References | **Conditions during intervention** | **Previous Recommendation: Avoid…** |
| Ozier and Linden. (2018)(81) | Open Eyes | NI |
| Pagaduan et al. (2019)(82) | Open Eyes | Caffeine and food (2h) |
| Pagaduan et al. (2021)(83) | Semi-supine Position, Open Eyes | Food (2h), Physical Activity, Caffeine (24h) |
| Park and Jung (2020)(84) | NI | NI |
| Patron et al. (2013)(85) | Light Attenuated, 21ºC, semi reclined, Open Eyes | NI |
| Patron et al. (2020)(86) | Dim light, Quiet Room, 21^O^C, Sitting, Open Eyes, 2 participants | Alcohol, Caffeine, Smoking (3h) |
| Paul and Garg (2012)(87) | Quiet Room, Sitting, Open Eyes | NI |
| Paul et al (2012)(88) | Quiet Room, Sitting, Open Eyes | NI |
| Penzlin et al (2015)(89) | Sitting, Open Eyes | NI |
| Pérez-Gaido et al. (2021)(90) | Sitting, Open Eyes | Medication, Physical Activity, Disrupted sleep, Alcohol, Tobacco, Food |
| Prinsloo et al. (2011)(91) | Sitting, Open Eyes | Heavy meals, Caffeine, Alcohol, Exercise (bef. 4h) |
| Pyne et al. (2019)(92) | Open Eyes, 15 participants | NI |
| Raymond et al. (2005)(93) | Open Eyes | NI |
| Reid et al. (2013)(94) | Open Eyes | NI |
| Reiner (2008)(95) | Open Eyes | NI |
| Reneau (2020)(96) | Open Eyes | NI |
| Rose et al (2021)(97) | Open Eyes | NI |
| Rusciano et al. (2017)(98) | Quiet Room, Sitting, Open Eyes | NI |
| Russell et al. (2017)(99) | Sitting, Open Eyes | NI |
| Sakakibara et al. (2013)(100) | Sitting, Open Eyes, Bedtime (home) | Alcohol (24h), and Caffeine and Strenuous Exercise (12h) |
| Schlatter et al (2021)(101) | Quiet Room, Open Eyes | NI |
| Schmidt et al. (2012)(102) | Open Eyes | NI |
| Schmidt et al. (2013)(103) | Quiet Room, Sitting | NI |
| Schuman and Killian (2019)(104) | NI | NI\| |
| Schuman et al (2019)(105) | Dim Light, Quiet Room, 22 ^O^C, Supine position, Open Eyes | NI |
| Shaw et al. (2012)(106) | Half session standing near the balance beam, Open Eyes | NI |
| Sherlin et al. (2010)(107) | Open Eyes | NI |
| Siepmann et al. (2008)(108) | Sitting, Open Eyes | NI |
| Siepmann et al. (2014)(109) | Sitting, Open Eyes | NI |
| References | **Conditions during intervention** | **Previous Recommendation: Avoid…** |
| Solarikova et al. (2016)(110) | NI | NI |
| Song and Lehrer (2003)(111) | Sitting, Open Eyes | NI |
| Sowder et al. (2010)(112) | NI | NI |
| Stanton et al (2019)(113) | Open Eyes | Physical Activity (6h), Caffeine (5h) |
| Steffen et al. (2017)(114) | Sitting, Open Eyes | NI |
| Steffen et al. (2021)(115) | Sitting | NI |
| Stern et al. (2014)(116) | Quiet Room, Sitting, Open Eyes | NI |
| Stromberg et al. (2015) (117) | Open Eyes | Alcohol, Food and Tobacco (1h) |
| Sutarto et al. (2012)(118) | Temperature Controlled, Open Eyes | NI |
| Sutarto et al. (2013)(119) | Sitting, Open Eyes | NI |
| Swanson et al. (2009)(120) | Nature Sounds, Sitting, Open Eyes | Alcohol, Caffeine and Exercise (4h) |
| Taghizadeh et al (2019)(121) | Quiet Room, Sitting, Open Eyes | NI |
| Tan et al. (2011)(122) | NI | NI |
| Tan et al. (2013)(123) | Open Eyes | NI |
| Tatschi et al (2020)(124) | Open Eyes | NI |
| Tavares et al. (2017)(125) | NI | NI |
| Teeravisutkul et al (2019)(126) | Open Eyes | NI |
| Tsai et al. (2015)(127) | Quiet Room, Sitting, Open Eyes, 10:00 to 16:00 (lab) and Before sleep (home) | Alcohol, Caffeine, Tobacco (during the whole experiment) |
| Vagedes et al. (2019)(128) | Open Eyes | NI |
| van de Zwan et al. (2015)(129) | NI | NI |
| van de Zwan et al. (2019)(130) | 2 to 6 participants | NI |
| van Diest et al. (2014)(131) | With headphones, Sitting, Open, 1 participant | NI |
| Wang et al. (2015)(132) | Open Eyes | NI |
| Wang et al. (2010)(133) | Quiet Room, 24-26ºC, 60-70% of humidity, Lying Down, At the same time of day | Alcohol, Caffeine, Tea and Spicy food (24h) |
| Weeks et al. (2015)(134) | Sitting, Open Eyes | NI |
| Wells et al. (2012)(135) | Light Attenuated, Open Eyes | Food and Drink (2h) and Caffeine (the day of the study) |
| Windthorst et al. (2017)(136) | Open Eyes, 1 participant | NI |
| Wu et al. (2012)(137) | 22-26ºC, Sitting, Open Eyes, 13:00 to 17:00 | Smoking and Coffee (12h) |
| References | **Conditions during intervention** | **Previous Recommendation: Avoid…** |
| You et al. (2021)(138) | Sitting, Open Eyes, All participants at the same time of day | Disrupted sleep routines, alcohol, exercise (24h), drink, eat (2h) |
| Yu et al. (2018)(139) | Light Attenuated, Temperature controlled, Open Eyes | NI |
| Yucha et al. (2005)(140) | Open Eyes | NI |
| Zauszniewski et al. (2013)(141) | Sitting, Open Eyes | NI |
| Zucker et al. (2009)(142) | Open Eyes | NI |
| Zunhammer et al. (2013)(143) | Quiet Room, Sitting, Open Eyes, At the same time of day | NI |

s=study; NI=Not Informed; NA=Not Applicable.
^1^=In brackets study 2; ^2^=Number of participants at the same time in the same room during the breathing intervention; ^3^=Hours, minutes or weeks before the breathing intervention.

**References from supplementary tables 5-7 (all studies included)**

Allen, B., & Friedman, B. H. (2012). Positive emotion reduces dyspnea during slow paced breathing. *Psychophysiology, 49*(5), 690-696. doi:10.1111/j.1469-8986.2011.01344.x

Amichai, T., Eylon, S., Berger, I., & Katz-Leurer, M. (2019). The impact of breathing rate on the cardiac autonomic dynamics among children with cerebral palsy compared to typically developed controls. *Dev Neurorehabil, 22*(2), 98-103. doi:10.1080/17518423.2018.1434700

Amjadian, M., Bahrami Ehsan, H., Saboni, K., Vahedi, S., Rostami, R., & Roshani, D. (2020). A pilot randomized controlled trial to assess the effect of Islamic spiritual intervention and of breathing technique with heart rate variability feedback on anxiety, depression and psycho-physiologic coherence in patients after coronary artery bypass surgery. *Ann Gen Psychiatry, 19*, 46. doi:10.1186/s12991-020-00296-1

Bartur, G., Vatine, J. J., Raphaely-Beer, N., Peleg, S., & Katz-Leurer, M. (2014). Heart rate autonomic regulation system at rest and during paced breathing among patients with CRPS as compared to age-matched healthy controls. *Pain Med, 15*(9), 1569-1574. doi:10.1111/pme.12449

Bates, M. E., Lesnewich, L. M., Uhouse, S. G., Gohel, S., & Buckman, J. F. (2019). Resonance-Paced Breathing Alters Neural Response to Visual Cues: Proof-of-Concept for a Neuroscience-Informed Adjunct to Addiction Treatments. *Front Psychiatry, 10*, 624. doi:10.3389/fpsyt.2019.00624

Beckham, A. J., Greene, T. B., & Meltzer-Brody, S. (2013). A pilot study of heart rate variability biofeedback therapy in the treatment of perinatal depression on a specialized perinatal psychiatry inpatient unit. *Arch Womens Ment Health, 16*(1), 59-65. doi:10.1007/s00737-012-0318-7

Brinkmann, A. E., Press, S. A., Helmert, E., Hautzinger, M., Khazan, I., & Vagedes, J. (2020). Comparing Effectiveness of HRV-Biofeedback and Mindfulness for Workplace Stress Reduction: A Randomized Controlled Trial. *Appl Psychophysiol Biofeedback, 45*(4), 307-322. doi:10.1007/s10484-020-09477-w

Burch, J. B., Ginsberg, J. P., McLain, A. C., Franco, R., Stokes, S., Susko, K., . . . O'Rourke, M. A. (2020). Symptom Management Among Cancer Survivors: Randomized Pilot Intervention Trial of Heart Rate Variability Biofeedback. *Appl Psychophysiol Biofeedback, 45*(2), 99-108. doi:10.1007/s10484-020-09462-3

Caldwell, Y. T., & Steffen, P. R. (2018). Adding HRV biofeedback to psychotherapy increases heart rate variability and improves the treatment of major depressive disorder. *Int J Psychophysiol, 131*, 96-101. doi:10.1016/j.ijpsycho.2018.01.001

Chalaye, P., Goffaux, P., Lafrenaye, S., & Marchand, S. (2009). Respiratory effects on experimental heat pain and cardiac activity. *Pain Med, 10*(8), 1334-1340. doi:10.1111/j.1526-4637.2009.00681.x

Chang, W. L., Lee, J. T., Li, C. R., Davis, A. H. T., Yang, C. C., & Chen, Y. J. (2020). Effects of Heart Rate Variability Biofeedback in Patients With Acute Ischemic Stroke: A Randomized Controlled Trial. *Biol Res Nurs, 22*(1), 34-44. doi:10.1177/1099800419881210

Chelidoni, O., Plans, D., Ponzo, S., Morelli, D., & Cropley, M. (2020). Exploring the Effects of a Brief Biofeedback Breathing Session Delivered Through the BioBase App in Facilitating Employee Stress Recovery: Randomized Experimental Study. *JMIR Mhealth Uhealth, 8*(10), e19412. doi:10.2196/19412

Chen, S., Sun, P., Wang, S., Lin, G., & Wang, T. (2016). Effects of heart rate variability biofeedback on cardiovascular responses and autonomic sympathovagal modulation following stressor tasks in prehypertensives. *J Hum Hypertens, 30*(2), 105-111. doi:10.1038/jhh.2015.27

Cheng, K. S., Croarkin, P. E., & Lee, P. F. (2019). Heart Rate Variability of Various Video-Aided Mindful Deep Breathing Durations and Its Impact on Depression, Anxiety, and Stress Symptom Severity. *Mindfulness, 10*(10), 2082-2094. doi:10.1007/s12671-019-01178-8

Clamor, A., Koenig, J., Thayer, J. F., & Lincoln, T. M. (2016). A randomized-controlled trial of heart rate variability biofeedback for psychotic symptoms. *Behav Res Ther, 87*, 207-215. doi:10.1016/j.brat.2016.10.003

Climov, D., Lysy, C., Berteau, S., Dutrannois, J., Dereppe, H., Brohet, C., & Melin, J. (2014). Biofeedback on heart rate variability in cardiac rehabilitation: practical feasibility and psycho-physiological effects. *Acta Cardiol, 69*(3), 299-307. doi:10.1080/ac.69.3.3027833

Cullins, S. W., Gevirtz, R. N., Poeltler, D. M., Cousins, L. M., Edward Harpin, R., & Muench, F. (2013). An exploratory analysis of the utility of adding cardiorespiratory biofeedback in the standard care of pregnancy-induced hypertension. *Appl Psychophysiol Biofeedback, 38*(3), 161-170. doi:10.1007/s10484-013-9219-4

De Couck, M., Caers, R., Musch, L., Fliegauf, J., Giangreco, A., & Gidron, Y. (2019). How breathing can help you make better decisions: Two studies on the effects of breathing patterns on heart rate variability and decision-making in business cases. *Int J Psychophysiol, 139*, 1-9. doi:10.1016/j.ijpsycho.2019.02.011

de Zambotti, M., Sizintsev, M., Claudatos, S., Barresi, G., Colrain, I. M., & Baker, F. C. (2019). Reducing bedtime physiological arousal levels using immersive audio-visual respiratory bio-feedback: a pilot study in women with insomnia symptoms. *J Behav Med, 42*(5), 973-983. doi:10.1007/s10865-019-00020-9

Del Pozo, J. M., Gevirtz, R. N., Scher, B., & Guarneri, E. (2004). Biofeedback treatment increases heart rate variability in patients with known coronary artery disease. *Am Heart J, 147*(3), E11. doi:10.1016/j.ahj.2003.08.013

Deschodt-Arsac, V., Blons, E., Gilfriche, P., Spiluttini, B., & Arsac, L. M. (2020). Entropy in Heart Rate Dynamics Reflects How HRV-Biofeedback Training Improves Neurovisceral Complexity during Stress-Cognition Interactions. *Entropy (Basel), 22*(3). doi:10.3390/e22030317

Deschodt-Arsac, V., Lalanne, R., Spiluttini, B., Bertin, C., & Arsac, L. M. (2018). Effects of heart rate variability biofeedback training in athletes exposed to stress of university examinations. *PLoS One, 13*(7), e0201388. doi:10.1371/journal.pone.0201388

Dessy, E., Mairesse, O., van Puyvelde, M., Cortoos, A., Neyt, X., & Pattyn, N. (2020). Train Your Brain? Can We Really Selectively Train Specific EEG Frequencies With Neurofeedback Training. *Front Hum Neurosci, 14*, 22. doi:10.3389/fnhum.2020.00022

Dziembowska, I., Izdebski, P., Rasmus, A., Brudny, J., Grzelczak, M., & Cysewski, P. (2016). Effects of Heart Rate Variability Biofeedback on EEG Alpha Asymmetry and Anxiety Symptoms in Male Athletes: A Pilot Study. *Appl Psychophysiol Biofeedback, 41*(2), 141-150. doi:10.1007/s10484-015-9319-4

Ebben, M. R., Kurbatov, V., & Pollak, C. P. (2009). Moderating laboratory adaptation with the use of a heart-rate variability biofeedback device (StressEraser). *Appl Psychophysiol Biofeedback, 34*(4), 245-249. doi:10.1007/s10484-009-9086-1

Eddie, D., Conway, F. N., Alayan, N., Buckman, J., & Bates, M. E. (2018). Assessing heart rate variability biofeedback as an adjunct to college recovery housing programs. *J Subst Abuse Treat, 92*, 70-76. doi:10.1016/j.jsat.2018.06.014

Eddie, D., Kim, C., Lehrer, P., Deneke, E., & Bates, M. E. (2014). A pilot study of brief heart rate variability biofeedback to reduce craving in young adult men receiving inpatient treatment for substance use disorders. *Appl Psychophysiol Biofeedback, 39*(3-4), 181-192. doi:10.1007/s10484-014-9251-z

Francis, H. M., Fisher, A., Rushby, J. A., & McDonald, S. (2016). Reduced heart rate variability in chronic severe traumatic brain injury: Association with impaired emotional and social functioning, and potential for treatment using biofeedback. *Neuropsychol Rehabil, 26*(1), 103-125. doi:10.1080/09602011.2014.1003246

Francis, H. M., Penglis, K. M., & McDonald, S. (2016). Manipulation of heart rate variability can modify response to anger-inducing stimuli. *Soc Neurosci, 11*(5), 545-552. doi:10.1080/17470919.2015.1115777

Giardino, N. D., Chan, L., & Borson, S. (2004). Combined heart rate variability and pulse oximetry biofeedback for chronic obstructive pulmonary disease: preliminary findings. *Appl Psychophysiol Biofeedback, 29*(2), 121-133. doi:10.1023/b:apbi.0000026638.64386.89

Gray, E., Beech, A., & Rose, J. (2019). Using biofeedback to improve emotion regulation in sexual offenders with intellectual disability: a feasibility study. *Int J Dev Disabil, 65*(3), 195-204. doi:10.1080/20473869.2018.1565003

Gross, M. J., Shearer, D. A., Bringer, J. D., Hall, R., Cook, C. J., & Kilduff, L. P. (2016). Abbreviated Resonant Frequency Training to Augment Heart Rate Variability and Enhance On-Demand Emotional Regulation in Elite Sport Support Staff. *Appl Psychophysiol Biofeedback, 41*(3), 263-274. doi:10.1007/s10484-015-9330-9

Gruzelier, J. H., Thompson, T., Redding, E., Brandt, R., & Steffert, T. (2014). Application of alpha/theta neurofeedback and heart rate variability training to young contemporary dancers: state anxiety and creativity. *Int J Psychophysiol, 93*(1), 105-111. doi:10.1016/j.ijpsycho.2013.05.004

Hallman, D. M., Olsson, E. M., von Scheele, B., Melin, L., & Lyskov, E. (2011). Effects of heart rate variability biofeedback in subjects with stress-related chronic neck pain: a pilot study. *Appl Psychophysiol Biofeedback, 36*(2), 71-80. doi:10.1007/s10484-011-9147-0

Hassett, A. L., Radvanski, D. C., Vaschillo, E. G., Vaschillo, B., Sigal, L. H., Karavidas, M. K., . . . Lehrer, P. M. (2007). A pilot study of the efficacy of heart rate variability (HRV) biofeedback in patients with fibromyalgia. *Appl Psychophysiol Biofeedback, 32*(1), 1-10. doi:10.1007/s10484-006-9028-0

Hasuo, H., Kanbara, K., & Fukunaga, M. (2020). Effect of Heart Rate Variability Biofeedback Sessions With Resonant Frequency Breathing on Sleep: A Pilot Study Among Family Caregivers of Patients With Cancer. *Front Med (Lausanne), 7*, 61. doi:10.3389/fmed.2020.00061

Hasuo, H., Kanbara, K., Sakuma, H., & Fukunaga, M. (2018). Awareness of comfort immediately after a relaxation therapy session affects future quality of life and autonomic function: a prospective cohort study on the expectations of therapy. *Biopsychosoc Med, 12*, 16. doi:10.1186/s13030-018-0135-y

Hasuo, H., Kanbara, K., Sakuma, H., Yoshida, K., Uchitani, K., & Fukunaga, M. (2019). Self-Care System for Family Caregivers of Cancer Patients Using Resonant Breathing with a Portable Home Device: A Randomized Open-Label Study. *J Palliat Med, 22*(1), 18-24. doi:10.1089/jpm.2018.0230

Hasuo, H., Kanbara, K., Shizuma, H., Morita, Y., & Fukunaga, M. (2020). Short-term efficacy of home-based heart rate variability biofeedback on sleep disturbance in patients with incurable cancer: a randomised open-label study. *BMJ Support Palliat Care*. doi:10.1136/bmjspcare-2020-002324

Henriques, G., Keffer, S., Abrahamson, C., & Horst, S. J. (2011). Exploring the effectiveness of a computer-based heart rate variability biofeedback program in reducing anxiety in college students. *Appl Psychophysiol Biofeedback, 36*(2), 101-112. doi:10.1007/s10484-011-9151-4

Hsieh, H. F., Huang, I. C., Liu, Y., Chen, W. L., Lee, Y. W., & Hsu, H. T. (2020). The Effects of Biofeedback Training and Smartphone-Delivered Biofeedback Training on Resilience, Occupational Stress, and Depressive Symptoms among Abused Psychiatric Nurses. *Int J Environ Res Public Health, 17*(8). doi:10.3390/ijerph17082905

Huang, A. J., Grady, D., Mendes, W. B., Hernandez, C., Schembri, M., & Subak, L. L. (2019). A Randomized Controlled Trial of Device Guided, Slow-Paced Respiration in Women with Overactive Bladder Syndrome. *J Urol, 202*(4), 787-794. doi:10.1097/JU.0000000000000328

Hunter, J. F., Olah, M. S., Williams, A. L., Parks, A. C., & Pressman, S. D. (2019). Effect of Brief Biofeedback via a Smartphone App on Stress Recovery: Randomized Experimental Study. *JMIR Serious Games, 7*(4), e15974. doi:10.2196/15974

Jester, D. J., Rozek, E. K., & McKelley, R. A. (2019). Heart rate variability biofeedback: implications for cognitive and psychiatric effects in older adults. *Aging Ment Health, 23*(5), 574-580. doi:10.1080/13607863.2018.1432031

Karavidas, M. K., Lehrer, P. M., Vaschillo, E., Vaschillo, B., Marin, H., Buyske, S., . . . Hassett, A. (2007). Preliminary results of an open label study of heart rate variability biofeedback for the treatment of major depression. *Appl Psychophysiol Biofeedback, 32*(1), 19-30. doi:10.1007/s10484-006-9029-z

Kennedy, J. J., & Pretorius, M. (2008). Integrating a Portable Biofeedback Device into Call Centre Environments to Reduce Employee Stress: Results from Two Pilot Studies. *Journal of Workplace Behavioral Health, 23*(3), 295-307. doi:10.1080/15555240802243096

Kohlenberg, S. (2020). Pilot Study of Subject Education and Oximetry as They Affect Comfort During Slow-Paced Breathing. *Appl Psychophysiol Biofeedback, 45*(1), 17-22. doi:10.1007/s10484-019-09451-1

Kudo, N., Shinohara, H., & Kodama, H. (2014). Heart rate variability biofeedback intervention for reduction of psychological stress during the early postpartum period. *Appl Psychophysiol Biofeedback, 39*(3-4), 203-211. doi:10.1007/s10484-014-9259-4

Laborde, S., Allen, M. S., Gohring, N., & Dosseville, F. (2017). The effect of slow-paced breathing on stress management in adolescents with intellectual disability. *J Intellect Disabil Res, 61*(6), 560-567. doi:10.1111/jir.12350

Laborde, S., Lentes, T., Hosang, T. J., Borges, U., Mosley, E., & Dosseville, F. (2019). Influence of Slow-Paced Breathing on Inhibition After Physical Exertion. *Front Psychol, 10*, 1923. doi:10.3389/fpsyg.2019.01923

Lee, J., & Finkelstein, J. (2015). Evaluation of a portable stress management device. *Stud Health Technol Inform, 208*, 248-252.

Lee, J., Kim, J. K., & Wachholtz, A. (2015). The benefit of heart rate variability biofeedback and relaxation training in reducing trait anxiety. *Hanguk Simni Hakhoe Chi Kongang, 20*, 391-408.

Lehrer, P., Karavidas, M. K., Lu, S. E., Coyle, S. M., Oikawa, L. O., Macor, M., . . . Lowry, S. F. (2010). Voluntarily produced increases in heart rate variability modulate autonomic effects of endotoxin induced systemic inflammation: an exploratory study. *Appl Psychophysiol Biofeedback, 35*(4), 303-315. doi:10.1007/s10484-010-9139-5

Lehrer, P., Vaschillo, E., Lu, S. E., Eckberg, D., Vaschillo, B., Scardella, A., & Habib, R. (2006). Heart rate variability biofeedback: effects of age on heart rate variability, baroreflex gain, and asthma. *Chest, 129*(2), 278-284. doi:10.1378/chest.129.2.278

Lehrer, P. M., Irvin, C. G., Lu, S. E., Scardella, A., Roehmheld-Hamm, B., Aviles-Velez, M., . . . Wamboldt, F. S. (2018). Heart Rate Variability Biofeedback Does Not Substitute for Asthma Steroid Controller Medication. *Appl Psychophysiol Biofeedback, 43*(1), 57-73. doi:10.1007/s10484-017-9382-0

Lehrer, P. M., Vaschillo, E., Vaschillo, B., Lu, S. E., Eckberg, D. L., Edelberg, R., . . . Hamer, R. M. (2003). Heart rate variability biofeedback increases baroreflex gain and peak expiratory flow. *Psychosom Med, 65*(5), 796-805. doi:10.1097/01.psy.0000089200.81962.19

Lehrer, P. M., Vaschillo, E., Vaschillo, B., Lu, S. E., Scardella, A., Siddique, M., & Habib, R. H. (2004). Biofeedback treatment for asthma. *Chest, 126*(2), 352-361. doi:10.1378/chest.126.2.352

Lewis, G. F., Hourani, L., Tueller, S., Kizakevich, P., Bryant, S., Weimer, B., & Strange, L. (2015). Relaxation training assisted by heart rate variability biofeedback: Implication for a military predeployment stress inoculation protocol. *Psychophysiology, 52*(9), 1167-1174. doi:10.1111/psyp.12455

Li, X., Zhang, T., Song, L.-P., Zhang, G.-G., Xing, C.-X., & Chen, H. (2015). Effects of Heart Rate Variability Biofeedback Therapy on Patients with Poststroke Depression: A Case Study. *Chin Med J (Engl), 128*, 2542-2545. doi:10.4103/0366‑6999.164986

Lin, G., Xiang, Q., Fu, X., Wang, S., Wang, S., Chen, S., . . . Wang, T. (2012). Heart rate variability biofeedback decreases blood pressure in prehypertensive subjects by improving autonomic function and baroreflex. *J Altern Complement Med, 18*(2), 143-152. doi:10.1089/acm.2010.0607

Lin, I. M. (2018). Effects of a cardiorespiratory synchronization training mobile application on heart rate variability and electroencephalography in healthy adults. *Int J Psychophysiol, 134*, 168-177. doi:10.1016/j.ijpsycho.2018.09.005

Lin, I. M., Fan, S. Y., Lu, H. C., Lin, T. H., Chu, C. S., Kuo, H. F., . . . Lu, Y. H. (2015). Randomized controlled trial of heart rate variability biofeedback in cardiac autonomic and hostility among patients with coronary artery disease. *Behav Res Ther, 70*, 38-46. doi:10.1016/j.brat.2015.05.001

Lin, I. M., Fan, S. Y., Yen, C. F., Yeh, Y. C., Tang, T. C., Huang, M. F., . . . Tsai, Y. C. (2019). Heart Rate Variability Biofeedback Increased Autonomic Activation and Improved Symptoms of Depression and Insomnia among Patients with Major Depression Disorder. *Clin Psychopharmacol Neurosci, 17*(2), 222-232. doi:10.9758/cpn.2019.17.2.222

Lin, I. M., Ko, J. M., Fan, S. Y., & Yen, C. F. (2016). Heart Rate Variability and the Efficacy of Biofeedback in Heroin Users with Depressive Symptoms. *Clin Psychopharmacol Neurosci, 14*(2), 168-176. doi:10.9758/cpn.2016.14.2.168

Lin, I. M., Tai, L. Y., & Fan, S. Y. (2014). Breathing at a rate of 5.5 breaths per minute with equal inhalation-to-exhalation ratio increases heart rate variability. *Int J Psychophysiol, 91*(3), 206-211. doi:10.1016/j.ijpsycho.2013.12.006

Lin, I. M., Wang, S. Y., Fan, S. Y., Peper, E., Chen, S. P., & Huang, C. Y. (2020). A Single Session of Heart Rate Variability Biofeedback Produced Greater Increases in Heart Rate Variability Than Autogenic Training. *Appl Psychophysiol Biofeedback, 45*(4), 343-350. doi:10.1007/s10484-020-09483-y

MacKinnon, S., Gevirtz, R., McCraty, R., & Brown, M. (2013). Utilizing heartbeat evoked potentials to identify cardiac regulation of vagal afferents during emotion and resonant breathing. *Appl Psychophysiol Biofeedback, 38*(4), 241-255. doi:10.1007/s10484-013-9226-5

Mason, E. B., Burkhart, K., & Lazebnik, R. (2019). Adolescent Stress Management in a Primary Care Clinic. *J Pediatr Health Care, 33*(2), 178-185. doi:10.1016/j.pedhc.2018.08.001

McAusland, L., & Addington, J. (2018). Biofeedback to treat anxiety in young people at clinical high risk for developing psychosis. *Early Interv Psychiatry, 12*(4), 694-701. doi:10.1111/eip.12368

Meier, N. F., & Welch, A. S. (2016). Walking versus biofeedback: a comparison of acute interventions for stressed students. *Anxiety Stress Coping, 29*(5), 463-478. doi:10.1080/10615806.2015.1085514

Meule, A., Freund, R., Skirde, A. K., Vogele, C., & Kubler, A. (2012). Heart rate variability biofeedback reduces food cravings in high food cravers. *Appl Psychophysiol Biofeedback, 37*(4), 241-251. doi:10.1007/s10484-012-9197-y

Meule, A., & Kubler, A. (2017). A Pilot Study on the Effects of Slow Paced Breathing on Current Food Craving. *Appl Psychophysiol Biofeedback, 42*(1), 59-68. doi:10.1007/s10484-017-9351-7

Meyer, P.-W., Friederich, H.-C., & Zastrow, A. (2018). Breathe to ease - Respiratory biofeedback to improve heart rate variability and coping with stress in obese patients: A pilot study. *Mental Health & Prevention, 11*, 41-46. doi:10.1016/j.mhp.2018.06.001

Minen, M. T., Corner, S., Berk, T., Levitan, V., Friedman, S., Adhikari, S., & Seng, E. B. (2021). Heartrate variability biofeedback for migraine using a smartphone application and sensor: A randomized controlled trial. *Gen Hosp Psychiatry, 69*, 41-49. doi:10.1016/j.genhosppsych.2020.12.008

Munafo, M., Patron, E., & Palomba, D. (2016). Improving Managers' Psychophysical Well-Being: Effectiveness of Respiratory Sinus Arrhythmia Biofeedback. *Appl Psychophysiol Biofeedback, 41*(2), 129-139. doi:10.1007/s10484-015-9320-y

Narita, Y., Shinohara, H., & Kodama, H. (2018). Resting Heart Rate Variability and the Effects of Biofeedback Intervention in Women with Low-Risk Pregnancy and Prenatal Childbirth Fear. *Appl Psychophysiol Biofeedback, 43*(2), 113-121. doi:10.1007/s10484-018-9389-1

Nelson Ferguson, K., Hall, C., & Divine, A. (2020). Examining the Effects of an Interspersed Biofeedback Training Intervention on Physiological Indices. *The Sport Psychologist, 34*(4), 310-318. doi:10.1123/tsp.2019-0111

Nolan, R. P., Floras, J. S., Harvey, P. J., Kamath, M. V., Picton, P. E., Chessex, C., . . . Chen, M. H. (2010). Behavioral neurocardiac training in hypertension: a randomized, controlled trial. *Hypertension, 55*(4), 1033-1039. doi:10.1161/HYPERTENSIONAHA.109.146233

Nolan, R. P., Kamath, M. V., Floras, J. S., Stanley, J., Pang, C., Picton, P., & Young, Q. R. (2005). Heart rate variability biofeedback as a behavioral neurocardiac intervention to enhance vagal heart rate control. *Am Heart J, 149*(6), 1137. doi:10.1016/j.ahj.2005.03.015

Ozier, D., & Linden, W. (2018). Heart Variability Biofeedback as Supplementary Care for Brain Cancer: A Feasibility Study. *J Altern Complement Med, 24*(8), 852-853. doi:10.1089/acm.2017.0393

Pagaduan, J., Wu, S. S., Kameneva, T., & Lambert, E. (2019). Acute effects of resonance frequency breathing on cardiovascular regulation. *Physiol Rep, 7*(22), e14295. doi:10.14814/phy2.14295

Pagaduan, J. C., Wu, S. S. X., Fell, J. W., & Chen, Y. S. (2021). Effect of Acute Heart Rate Variability Biofeedback on H-reflex Modulation: A Pilot Study. *J Hum Kinet, 76*, 83-88. doi:10.2478/hukin-2021-0001

Park, S. M., & Jung, H. Y. (2020). Respiratory sinus arrhythmia biofeedback alters heart rate variability and default mode network connectivity in major depressive disorder: A preliminary study. *Int J Psychophysiol, 158*, 225-237. doi:10.1016/j.ijpsycho.2020.10.008

Patron, E., Messerotti Benvenuti, S., Favretto, G., Valfrè, C., Bonfà, C., Gasparotto, R., & Palomba, D. (2013). Biofeedback assisted control of respiratory sinus arrhythmia as a biobehavioral intervention for depressive symptoms in patients after cardiac surgery: a preliminary study. *Appl Psychophysiol Biofeedback, 38*(1), 1-9. doi:10.1007/s10484-012-9202-5

Patron, E., Munafo, M., Messerotti Benvenuti, S., Stegagno, L., & Palomba, D. (2020). Not All Competitions Come to Harm! Competitive Biofeedback to Increase Respiratory Sinus Arrhythmia in Managers. *Front Neurosci, 14*, 855. doi:10.3389/fnins.2020.00855

Paul, M., & Garg, K. (2012). The effect of heart rate variability biofeedback on performance psychology of basketball players. *Appl Psychophysiol Biofeedback, 37*(2), 131-144. doi:10.1007/s10484-012-9185-2

Paul, M., Garg, K., & Singh Sandhu, J. (2012). Role of biofeedback in optimizing psychomotor performance in sports. *Asian J Sports Med, 3*(1), 29-40. doi:10.5812/asjsm.34722

Penzlin, A. I., Siepmann, T., Illigens, B. M., Weidner, K., & Siepmann, M. (2015). Heart rate variability biofeedback in patients with alcohol dependence: a randomized controlled study. *Neuropsychiatr Dis Treat, 11*, 2619-2627. doi:10.2147/NDT.S84798

Perez-Gaido, M., Lalanza, J. F., Parrado, E., & Capdevila, L. (2021). Can HRV Biofeedback Improve Short-Term Effort Recovery? Implications for Intermittent Load Sports. *Appl Psychophysiol Biofeedback, 46*(2), 215-226. doi:10.1007/s10484-020-09495-8

Prinsloo, G. E., Rauch, H. G. L., Lambert, M. I., Muench, F., Noakes, T. D., & Derman, W. E. (2011). The effect of short duration heart rate variability (HRV) biofeedback on cognitive performance during laboratory induced cognitive stress. *Applied Cognitive Psychology, 25*(5), 792-801. doi:10.1002/acp.1750

Pyne, J. M., Constans, J. I., Nanney, J. T., Wiederhold, M. D., Gibson, D. P., Kimbrell, T., . . . McCune, T. R. (2019). Heart Rate Variability and Cognitive Bias Feedback Interventions to Prevent Post-deployment PTSD: Results from a Randomized Controlled Trial. *Mil Med, 184*(1-2), e124-e132. doi:10.1093/milmed/usy171

Raymond, J., Sajid, I., Parkinson, L. A., & Gruzelier, J. H. (2005). Biofeedback and dance performance: a preliminary investigation. *Appl Psychophysiol Biofeedback, 30*(1), 64-73. doi:10.1007/s10484-005-2175-x

Reid, A., Nihon, S., Thompson, L., & Thompson, M. (2013). The Effects of Heart Rate Variability Training on Sensorimotor Rhythm: A Pilot Study. *Journal of Neurotherapy, 17*(1), 43-48. doi:10.1080/10874208.2013.759020

Reiner, R. (2008). Integrating a portable biofeedback device into clinical practice for patients with anxiety disorders: results of a pilot study. *Appl Psychophysiol Biofeedback, 33*(1), 55-61. doi:10.1007/s10484-007-9046-6

Reneau, M. (2020). Feasibility and Acceptability of Heart Rate Variability Biofeedback in a Group of Veterans with Fibromyalgia. *J Altern Complement Med, 26*(11), 1025-1031. doi:10.1089/acm.2020.0071

Reyes del Paso, G. A., Cea, J. I., Gonzalez-Pinto, A., Cabo, O. M., Caso, R., Brazal, J., . . . Gonzalez, M. I. (2006). Short-term effects of a brief respiratory training on baroreceptor cardiac reflex function in normotensive and mild hypertensive subjects. *Appl Psychophysiol Biofeedback, 31*(1), 37-49. doi:10.1007/s10484-006-9003-9

Rose, S., Cacho, F., Wiersma, L., Magdaleno, A., Anderson, N., & Statler, T. (2021). Efficacy of a Brief Biofeedback Intervention on Mood, Arousal, Mental Workload, Movement Time, and Biofeedback Device Preference. *Appl Psychophysiol Biofeedback, 46*(2), 205-214. doi:10.1007/s10484-020-09500-0

Rusciano, A., Corradini, G., & Stoianov, I. (2017). Neuroplus biofeedback improves attention, resilience, and injury prevention in elite soccer players. *Psychophysiology, 54*(6), 916-926. doi:10.1111/psyp.12847

Russell, M. E., Scott, A. B., Boggero, I. A., & Carlson, C. R. (2017). Inclusion of a rest period in diaphragmatic breathing increases high frequency heart rate variability: Implications for behavioral therapy. *Psychophysiology, 54*(3), 358-365. doi:10.1111/psyp.12791

Sakakibara, M., Hayano, J., Oikawa, L. O., Katsamanis, M., & Lehrer, P. (2013). Heart rate variability biofeedback improves cardiorespiratory resting function during sleep. *Appl Psychophysiol Biofeedback, 38*(4), 265-271. doi:10.1007/s10484-013-9232-7

Schlatter, S., Schmidt, L., Lilot, M., Guillot, A., & Debarnot, U. (2021). Implementing biofeedback as a proactive coping strategy: Psychological and physiological effects on anticipatory stress. *Behav Res Ther, 140*, 103834. doi:10.1016/j.brat.2021.103834

Schmidt, J. E., Joyner, M. J., Carlson, C. R., & Hooten, W. M. (2013). Cardiac autonomic function associated with treatment adherence after a brief intervention in patients with chronic pain. *Appl Psychophysiol Biofeedback, 38*(3), 193-201. doi:10.1007/s10484-013-9222-9

Schmidt, J. E., Joyner, M. J., Tonyan, H. M., Reid, K. I., & Hooten, W. M. (2012). Psychological and Physiological Correlates of a Brief Intervention to Enhance Self-Regulation in Patients with Fibromyalgia. *Journal of Musculoskeletal Pain, 20*(3), 211-221. doi:10.3109/10582452.2012.704142

Schuman, D. L., & Killian, M. O. (2019). Pilot Study of a Single Session Heart Rate Variability Biofeedback Intervention on Veterans' Posttraumatic Stress Symptoms. *Appl Psychophysiol Biofeedback, 44*(1), 9-20. doi:10.1007/s10484-018-9415-3

Schumann, A., Kohler, S., Brotte, L., & Bar, K. J. (2019). Effect of an eight-week smartphone-guided HRV-biofeedback intervention on autonomic function and impulsivity in healthy controls. *Physiol Meas, 40*(6), 064001. doi:10.1088/1361-6579/ab2065

Shaw, L., & Zaichkowsky, L. (2012). Setting the Balance: Using Biofeedback and Neurofeedback With Gymnasts. *Journal of Clinical Sport Psychology, 6*, 47-66.

Sherlin, L., Muench, F., & Wyckoff, S. (2010). Respiratory sinus arrhythmia feedback in a stressed population exposed to a brief stressor demonstrated by quantitative EEG and sLORETA. *Appl Psychophysiol Biofeedback, 35*(3), 219-228. doi:10.1007/s10484-010-9132-z

Siepmann, M., Aykac, V., Unterdorfer, J., Petrowski, K., & Mueck-Weymann, M. (2008). A pilot study on the effects of heart rate variability biofeedback in patients with depression and in healthy subjects. *Appl Psychophysiol Biofeedback, 33*(4), 195-201. doi:10.1007/s10484-008-9064-z

Siepmann, M., Hennig, U. D., Siepmann, T., Nitzsche, K., Muck-Weymann, M., Petrowski, K., & Weidner, K. (2014). The effects of heart rate variability biofeedback in patients with preterm labour. *Appl Psychophysiol Biofeedback, 39*(1), 27-35. doi:10.1007/s10484-013-9238-1

Solarikova, P., Mlyncekova, S., & Turonova, J. R. (2016). HRV biofeedback training in allergic patients and anxious individuals: A pilot study. *Activitas Nervosa Superior Rediviva, 58*, 110-114.

Song, H. S., & Lehrer, P. M. (2003). The effects of specific respiratory rates on heart rate and heart rate variability. *Appl Psychophysiol Biofeedback, 28*(1), 13-23. doi:10.1023/a:1022312815649

Sowder, E., Gevirtz, R., Shapiro, W., & Ebert, C. (2010). Restoration of vagal tone: a possible mechanism for functional abdominal pain. *Appl Psychophysiol Biofeedback, 35*(3), 199-206. doi:10.1007/s10484-010-9128-8

Stanton, A. M., Boyd, R. L., Fogarty, J. J., & Meston, C. M. (2019). Heart rate variability biofeedback increases sexual arousal among women with female sexual arousal disorder: Results from a randomized-controlled trial. *Behav Res Ther, 115*, 90-102. doi:10.1016/j.brat.2018.10.016

Steffen, P. R., Austin, T., DeBarros, A., & Brown, T. (2017). The Impact of Resonance Frequency Breathing on Measures of Heart Rate Variability, Blood Pressure, and Mood. *Front Public Health, 5*, 222. doi:10.3389/fpubh.2017.00222

Steffen, P. R., Bartlett, D., Channell, R. M., Jackman, K., Cressman, M., Bills, J., & Pescatello, M. (2021). Integrating Breathing Techniques Into Psychotherapy to Improve HRV: Which Approach Is Best? *Front Psychol, 12*, 624254. doi:10.3389/fpsyg.2021.624254

Stern, M. J., Guiles, R. A., & Gevirtz, R. (2014). HRV biofeedback for pediatric irritable bowel syndrome and functional abdominal pain: a clinical replication series. *Appl Psychophysiol Biofeedback, 39*(3-4), 287-291. doi:10.1007/s10484-014-9261-x

Stromberg, S. E., Russell, M. E., & Carlson, C. R. (2015). Diaphragmatic breathing and its effectiveness for the management of motion sickness. *Aerosp Med Hum Perform, 86*(5), 452-457. doi:10.3357/AMHP.4152.2015

Sutarto, A. P., Wahab, M. N., & Zin, N. M. (2012). Resonant breathing biofeedback training for stress reduction among manufacturing operators. *Int J Occup Saf Ergon, 18*(4), 549-561. doi:10.1080/10803548.2012.11076959

Sutarto, A. P., Wahab, M. N., & Zin, N. M. (2013). Effect of biofeedback training on operator's cognitive performance. *Work, 44*(2), 231-243. doi:10.3233/WOR-121499

Swanson, K. S., Gevirtz, R. N., Brown, M., Spira, J., Guarneri, E., & Stoletniy, L. (2009). The effect of biofeedback on function in patients with heart failure. *Appl Psychophysiol Biofeedback, 34*(2), 71-91. doi:10.1007/s10484-009-9077-2

Taghizadeh, N., Eslaminejad, A., & Raoufy, M. R. (2019). Protective effect of heart rate variability biofeedback on stress-induced lung function impairment in asthma. *Respir Physiol Neurobiol, 262*, 49-56. doi:10.1016/j.resp.2019.01.011

Tan, G., Dao, T. K., Farmer, L., Sutherland, R. J., & Gevirtz, R. (2011). Heart rate variability (HRV) and posttraumatic stress disorder (PTSD): a pilot study. *Appl Psychophysiol Biofeedback, 36*(1), 27-35. doi:10.1007/s10484-010-9141-y

Tan, X. L., Wang, S., & Zhang, F. (2013). Optimization an optimal artificial diet for the predatory bug Orius sauteri (hemiptera: anthocoridae). *PLoS One, 8*(4), e61129. doi:10.1371/journal.pone.0061129

Tatschl, J. M., Hochfellner, S. M., & Schwerdtfeger, A. R. (2020). Implementing Mobile HRV Biofeedback as Adjunctive Therapy During Inpatient Psychiatric Rehabilitation Facilitates Recovery of Depressive Symptoms and Enhances Autonomic Functioning Short-Term: A 1-Year Pre-Post-intervention Follow-Up Pilot Study. *Front Neurosci, 14*, 738. doi:10.3389/fnins.2020.00738

Tavares, B. S., de Paula Vidigal, G., Garner, D. M., Raimundo, R. D., de Abreu, L. C., & Valenti, V. E. (2017). Effects of guided breath exercise on complex behaviour of heart rate dynamics. *Clin Physiol Funct Imaging, 37*(6), 622-629. doi:10.1111/cpf.12347

Teeravisutkul, P., Chumchua, V., Saengcharnchai, P., & Leelahanaj, T. (2019). Stress and craving reduction under treatment with heart rate variability biofeedback and the Phramongkutklao model among patients with alcohol use disorder. *Psychol Res Behav Manag, 12*, 619-627. doi:10.2147/PRBM.S199762

Tsai, H. J., Kuo, T. B., Lee, G. S., & Yang, C. C. (2015). Efficacy of paced breathing for insomnia: enhances vagal activity and improves sleep quality. *Psychophysiology, 52*(3), 388-396. doi:10.1111/psyp.12333

Vagedes, J., Fazeli, A., Boening, A., Helmert, E., Berger, B., & Martin, D. (2019). Efficacy of rhythmical massage in comparison to heart rate variability biofeedback in patients with dysmenorrhea-A randomized, controlled trial. *Complement Ther Med, 42*, 438-444. doi:10.1016/j.ctim.2018.11.009

van der Zwan, J. E., de Vente, W., Huizink, A. C., Bogels, S. M., & de Bruin, E. I. (2015). Physical activity, mindfulness meditation, or heart rate variability biofeedback for stress reduction: a randomized controlled trial. *Appl Psychophysiol Biofeedback, 40*(4), 257-268. doi:10.1007/s10484-015-9293-x

van der Zwan, J. E., Huizink, A. C., Lehrer, P. M., Koot, H. M., & de Vente, W. (2019). The Effect of Heart Rate Variability Biofeedback Training on Mental Health of Pregnant and Non-Pregnant Women: A Randomized Controlled Trial. *Int J Environ Res Public Health, 16*(6). doi:10.3390/ijerph16061051

Van Diest, I., Verstappen, K., Aubert, A. E., Widjaja, D., Vansteenwegen, D., & Vlemincx, E. (2014). Inhalation/Exhalation ratio modulates the effect of slow breathing on heart rate variability and relaxation. *Appl Psychophysiol Biofeedback, 39*(3-4), 171-180. doi:10.1007/s10484-014-9253-x

Wang, S. Z., Li, S., Xu, X. Y., Lin, G. P., Shao, L., Zhao, Y., & Wang, T. H. (2010). Effect of slow abdominal breathing combined with biofeedback on blood pressure and heart rate variability in prehypertension. *J Altern Complement Med, 16*(10), 1039-1045. doi:10.1089/acm.2009.0577

Wang, Y., Wu, W., Zhang, H., & Zhang, Y.-T. (2015). A Comparative Study of Heart Rate Variability Bio-Feedback Based on Unemployed and Normal Subject Groups. *Journal of Medical Imaging and Health Informatics, 5*(1), 158-164. doi:10.1166/jmihi.2015.1372

Weeks, D. L., Whitney, A. A., Tindall, A. G., & Carter, G. T. (2015). Pilot Randomized Trial Comparing Intersession Scheduling of Biofeedback Results to Individuals with Chronic Pain: Influence on Psychologic Function and Pain Intensity. *Am J Phys Med Rehabil, 94*(10 Suppl 1), 869-878. doi:10.1097/PHM.0000000000000285

Wells, R., Outhred, T., Heathers, J. A., Quintana, D. S., & Kemp, A. H. (2012). Matter over mind: a randomised-controlled trial of single-session biofeedback training on performance anxiety and heart rate variability in musicians. *PLoS One, 7*(10), e46597. doi:10.1371/journal.pone.0046597

Windthorst, P., Mazurak, N., Kuske, M., Hipp, A., Giel, K. E., Enck, P., . . . Teufel, M. (2017). Heart rate variability biofeedback therapy and graded exercise training in management of chronic fatigue syndrome: An exploratory pilot study. *J Psychosom Res, 93*, 6-13. doi:10.1016/j.jpsychores.2016.11.014

Wu, W., Gil, Y., & Lee, J. (2012). Combination of wearable multi-biosensor platform and resonance frequency training for stress management of the unemployed population. *Sensors (Basel), 12*(10), 13225-13248. doi:10.3390/s121013225

You, M., Laborde, S., Salvotti, C., Zammit, N., Mosley, E., & Dosseville, F. (2021). Influence of a Single Slow-Paced Breathing Session on Cardiac Vagal Activity in Athletes. *International Journal of Mental Health and Addiction*. doi:10.1007/s11469-020-00467-x

Yu, L. C., Lin, I. M., Fan, S. Y., Chien, C. L., & Lin, T. H. (2018). One-Year Cardiovascular Prognosis of the Randomized, Controlled, Short-Term Heart Rate Variability Biofeedback Among Patients with Coronary Artery Disease. *Int J Behav Med, 25*(3), 271-282. doi:10.1007/s12529-017-9707-7

Yucha, C. B., Tsai, P.-S., Calderon, K. S., & Tian, L. (2005). Biofeedback-assisted Relaxation Training for Essential Hypertension Who Is Most Likely to Benefit? *Journal of Cardiovascular Nursing, 20*, 198-205.

Zauszniewski, J. A., Au, T.-Y., & Musil, C. M. (2013). Heart Rate Variability Biofeedback in Grandmothers Raising Grandchildren: Effects on Stress, Emotions, and Cognitions. *Biofeedback, 41*(3), 144-149. doi:10.5298/1081-5937-41.3.06

Zucker, T. L., Samuelson, K. W., Muench, F., Greenberg, M. A., & Gevirtz, R. N. (2009). The effects of respiratory sinus arrhythmia biofeedback on heart rate variability and posttraumatic stress disorder symptoms: a pilot study. *Appl Psychophysiol Biofeedback, 34*(2), 135-143. doi:10.1007/s10484-009-9085-2

Zunhammer, M., Eichhammer, P., & Busch, V. (2013). Do cardiorespiratory variables predict the antinociceptive effects of deep and slow breathing? *Pain Medicine, 14*, 843-854.
